# Directed Evolution of Enzymes for Bioorthogonal Chemistry Using Acid Chloride Proximity Labeling

**DOI:** 10.1021/acscentsci.5c01746

**Published:** 2026-01-23

**Authors:** Ashley N. Ogorek, Shubhashree Pani, Eli J. Mertick-Sykes, Jelena Momirov, Yichong Lao, Fernando Banales Mejia, Rachel S. T. Chan, Xuhui Huang, Bryan C. Dickinson, Jeffrey D. Martell

**Affiliations:** † Department of Chemistry, 5228 University of Wisconsin−Madison, Madison, Wisconsin 53706, United States; ‡ Department of Chemistry, 2462 University of Chicago, 5735 S. Ellis Ave., Chicago, Illinois 60637, United States; § Chan Zuckerberg Biohub, Chicago, Illinois 60642, United States; ∥ Carbone Cancer Center, University of Wisconsin School of Medicine and Public Health, Madison, Wisconsin 53706, United States

## Abstract

Combining bioorthogonal protecting groups with localized catalysts that can unmask them is a powerful approach to spatially and temporally modulate molecular activity. Enzymes are appealing catalysts in this context because they are genetically targetable, but enzymes are not always available to unmask a protecting group of interest. Here, we report a platform for ultrahigh-throughput enzyme evolution by combining yeast surface display with masked acylating probes, which selectively label yeast cells based on target biocatalytic activity. We introduce the phenylcyclopropyl (pCP) ester protecting group, which has improved bioorthogonality compared to existing ester protecting groups, and use our platform to evolve BS2 esterase for enhanced pCP unmasking. Evolved BS2 mutants are up to 232-fold more active toward the pCP group. Taking advantage of the enhanced bioorthogonality of the pCP group, we applied a pCP probe together with evolved BS2 to perform spatially resolved RNA tagging with high spatial specificity, including in mammalian cell lines with high endogenous esterase activity. Overall, this work delivers a new bioorthogonal protecting group and engineered enzymes capable of unmasking it, and more broadly, it provides a platform to rapidly engineer enzymes for protecting group removal, opening opportunities in imaging, proximity tagging, induced cell signaling, and therapeutics.

## Graphical abstract

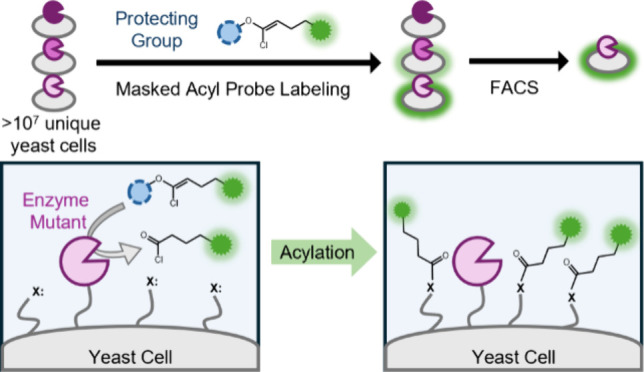

## Introduction

Protecting groups are powerful tools to modulate the activity of small molecules, peptides, and proteins. Some protecting groups cloak molecular cargoes to enhance delivery into cells, relying on the endogenous intracellular environment or the tumor microenvironment to unmask the molecular cargo and restore its activity.^1–3^ Other protecting groups are stable under biological conditions (i.e., bioorthogonal) and removable by an external factor, thus allowing molecular activity to be triggered at a specific time or location.^4–6^ Depending on the protecting group-masked molecule, its unmasking can initiate different effects, including luminescence for imaging, cell signaling activation, and therapeutic treatment.^7–9^

One strategy for triggering the removal of bioorthogonal protecting groups is to target a catalyst to a specific biological location, leading to site-selective unmasking of the molecular cargo.^10^ This strategy has several requirements: 1) a protecting group with high stability in physiological conditions, 2) a catalyst with high activity for unmasking the protecting group, ideally when installed on different molecules, and 3) a means to target the catalyst to precise locations within biological systems. For example, an organocatalyst was reported for nitro reduction leading to prodrug activation;^11^ abiotic transition metal catalysts have been developed for the removal of propargyl and allyl carbamate groups or the reduction of azides;^12–19^ artificial metalloenzymes have been created with metal catalysts bound to protein scaffolds;^20–22^ and nitroreductase enzymes have been deployed to reduce nitro groups to amines for a cascade deprotection that unveils molecular cargoes.^23^ Each of these catalyst/protecting group pairs has limitations, such as biocompatibility, difficulty with genetic expression, or the need for cofactors.

Esterase enzymes that operate without the need for any cofactors can also enable bioorthogonal protecting group removal. Although not all esters are bioorthogonal, certain bulky abiotic esters, most notably 1-methylcyclopropyl (mCP) esters, have been reported to resist hydrolysis within human cells.^24^ However, it was discovered that pig liver esterase (PLE) and *Bacillus subtilis* esterase (BS2) fortuitously are able to cleave the mCP ester and unmask small molecules protected with the mCP group even in living *E. coli* and mammalian cells. These enzymes and corresponding mCP-masked molecules have subsequently been used to develop technologies in which the catalysts are targeted to specific subcellular locations for site-selective unmasking of imaging agents or bioactive molecules (Figure 1a).^24–28^ However, despite the enhanced bioorthogonality of mCP ester relative to other previously reported esters, molecules protected by the mCP group are still susceptible to unmasking inside human cells outside the biological location of interest, resulting in background activity (*vide infra*). While larger esters could potentially be more bioorthogonal, it is unlikely that a natural enzyme could be identified that processes the larger esters while having other desirable features such as easy expression in diverse cell lines. BS2 esterase in particular is highly advantageous for bioorthogonal unmasking, as it is moderately sized (54 kDa), can be expressed in different compartments of mammalian cells, and is easily purifiable. While BS2 can process a wide array of diverse ester substrates, including mCP, it still has limitations on what esters can be efficiently cleaved. We therefore reasoned that if we could discover more bioorthogonal esters and develop new evolution-based methods to endow enzymes such as BS2 with the ability to process those esters, we could create improved bioorthogonal enzyme/substrate pairs.

**Figure 1:**
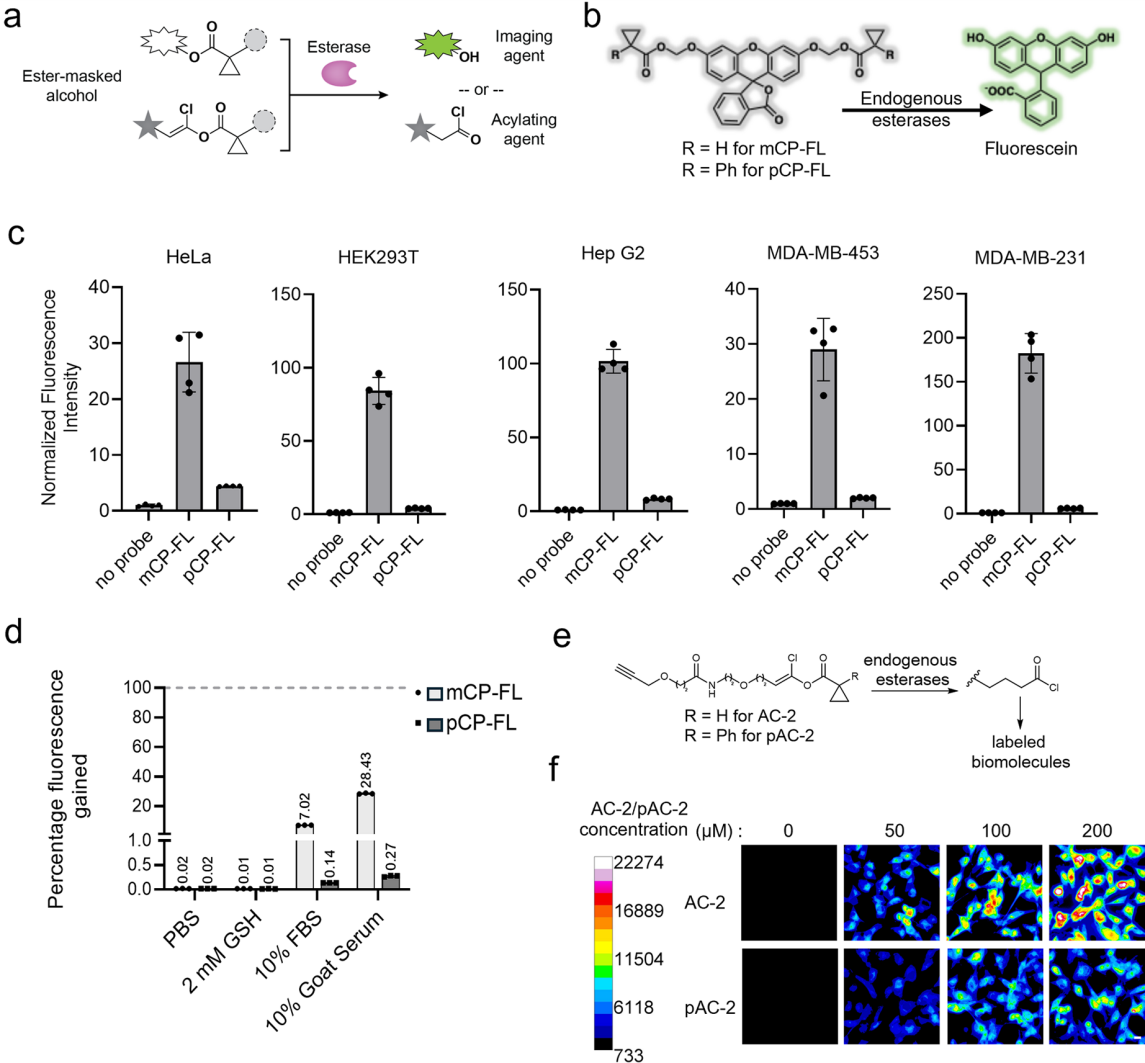
pCP is an improved bioorthogonal mask. (a) Schematic diagram representing cargo molecules protected by a bulky ester mask, which are converted by selective esterases to imaging agents or acylating probes. (b) Reaction scheme representing the unmasking of ester-masked fluorescein probes by endogenous esterases, leading to fluorescence turn-on. (c) *In cellulo* activity of masked-fluorescein probes (20 μM each) monitored by fluorescence microscopy across different cell lines. *n* = 4 different fields of view; error bars are the standard deviation. (d) Comparison of stability profiles of mCP and pCP-fluorescein in different contexts. The mCP- and pCP-fluorescein probes were incubated in respective buffers at 37 °C, and fluorescence was measured after 24 h. The percentage of fluorescence gained was assessed in comparison to the fluorescence from complete ester hydrolysis of respective probes with 0.33 N NaOH. *n* = 3 replicate measurements in the same plate. (e) Reaction scheme for labeling by acid chloride probes after endogenous esterase unmasking. (f) Background activity of masked acid chloride probes in MDA-MB-231 cells. Different concentrations of AC-2 and pAC-2 probes were incubated for 5 min, and background activity was measured by immunofluorescence imaging post click reaction with azide-fluorophore AF488. Fluorescence signal from the samples without the probe treatment was used for normalization. The calibration bar on the left shows fluorescence units corresponding to the respective colors. Scale bar on the bottom right image represents 10 μm. *n* = 5 fields of view.

Here, we report **D**irected **E**volution of **E**nzymes via **M**asked **A**cid **Ch**loride **P**robes (″DEEPMACh”), a platform that merges yeast surface display with proximity labeling chemistry. Specifically, DEEPMACh employs enol-ester masked acid chloride probes, which we previously reported in the development of the BAP-seq method for RNA proximity labeling.^29^ DEEPMACh screens millions of yeast cells that express enzyme variants and self-tag based on intended biocatalytic activity, followed by fluorescence activated cell sorting (FACS). We identified the phenyl-cyclopropyl (pCP) ester as an improved bioorthogonal protecting group in mammalian cells, but we found that BS2 esterase has very low activity on pCP. Evolution using DEEPMACh on BS2 esterase rapidly produced variants with >230-fold enhancement in enzymatic activity for unmasking the pCP ester protecting group. We demonstrate that the novel pCP protecting group can be used together with evolved BS2 for BAP-seq,^29^ which entails the spatially resolved release of acid chlorides in living cells for RNA proximity labeling and subcellular transcriptomics. Improved bioorthogonal masks with desired properties, combined with the ability to rapidly evolve corresponding enzymes, open new opportunities in a range of biotechnology applications, spanning imaging, systems biology, therapeutics, and more.

## Results and Discussion

### pCP is an Improved Bioorthogonal Ester

Before establishing our evolution platform, we first identified bulkier versions of the mCP protecting group as a promising target for enzymatic evolution. While mCP was originally identified as a “bioorthogonal ester”^24^ and has been used in a variety of technologies,^27,28^ including in our hands,^25,29^ we have noted some background activity in mammalian cells. Indeed, we observed significant unmasking of an mCP bis-caged AM ester fluorescein probe in a variety of common human cell lines (Figure 1b,c). Therefore, we synthesized a panel of bis-caged AM ester fluorescein probes and assessed their endogenous activation in HepG2 cells (Supplementary Figures 1 and 2). Several protecting groups were more stable than mCP, and the pCP-fluorescein (pCP-FL) probe gave the least endogenous unmasking, with almost undetectable fluorescence. We therefore identified the pCP protecting group as a promising candidate for further investigation of endogenous stability.

pCP-FL gave significantly lower endogenous unmasking (6–30-fold lower) than mCP-FL in HeLa, HEK293T, HepG2, MDA-MD-453, and MDA-MB-231 cell lines (Figure 1c and Supplementary Figures 3–7). Additionally, while both mCP-FL and pCP-FL were stable in PBS buffer and glutathione solution, pCP-FL gave 50–100-fold lower fluorescence than mCP-FL in 10% fetal bovine serum (FBS) (Figure 1d) and >10-fold higher stability in cell lysates at 37 °C (Supplementary Figure 8). We tested the stability of the mCP and pCP groups in a more labile system by masking 7-hydroxycoumarin, in which probe hydrolysis yields a fluorescent phenolate. mCP-coumarin was highly sensitive to glutathione, serum, and especially 10% FBS at 37 °C, which led to almost quantitative deprotection of the mCP ester group in 4 h. Meanwhile, pCP-coumarin showed substantially improved stability. To assess the stability of the pCP group in the context of masked acid chloride probes, we synthesized a pCP-bearing masked acid chloride probe (pAC-2) for comparison with AC-2, the previously reported masked acid chloride featuring the mCP protecting group (Figure 1e). We observed lower background for pAC-2 compared to AC-2 in HEK293T and MDA-MB-231 cells when treated for 5–15 min (Figure 1f and Supplementary Figure 9). Overall, the enhanced bioorthogonality of the pCP group may be due to steric factors as opposed to electronic factors, considering that the pCP group exhibited improved stability relative to the mCP group in probes featuring leaving groups with varying electronic structures: coumarin (phenolate leaving group), fluorescein AM ester (alkoxide leaving group), and masked acid chloride probes (enolate leaving group).

We next tested if BS2^WT^ could unmask pCP esters. BS2 exhibits dramatically reduced activity toward the pCP probe compared to the mCP probe, in both cellular assays and *in vitro* measurements of catalytic activity (*vide infra)*. This lack of activity motivated us to develop the DEEPMACh platform and apply it to evolve BS2 for unmasking the pCP protecting group.

### Development and Optimization of DEEPMACh

We aimed to develop a directed evolution approach for reprogramming BS2 to cleave bulkier esters. Ultrahigh-throughput directed evolution (evaluating >10^7^ mutants) is appealing because it enables deep exploration of enzyme sequence space compared to conventional screening methods.^30–32^ Previously, we used the phage-assisted continuous evolution (PACE) platform for ultrahigh-throughput evolution of enzymes for protecting group removal.^33^ Others have reported a similar approach with a cell sorting platform relying on a fluorogenic signal inside living bacteria.^34^ However, it was challenging in these previous platforms to control reaction time and substrate concentration, which limited the stringency of the selections and resulted in ∼ 5–15-fold activity enhancement, not >100-fold as may be needed for robust molecular activation to exert selective biological effects within living cells.

We selected yeast surface display as the context for enzyme expression in developing the DEEPMACh platform because it allows screening of millions of enzyme mutants from a library in a single test tube.^35^ Furthermore, in contrast to previous platforms that require intracellular reactions,^25^ yeast display enables extracellular reactions with stringent selection conditions, featuring precise control over substrate concentration and reaction time. Although yeast display directed evolution (DE) has been widely demonstrated for the evolution of proteins to bind specific targets,^36^ there are limited examples of its usage to evolve enzymatic catalysts, especially for bond-breaking enzymes. Existing yeast surface catalysis platforms have focused on evolving enzymes for bond-forming reactions, such as sortase peptide ligation or polyketide synthase module evolution,^37–39^ or on the generation of phenoxy radicals that covalently cross-link to endogenous tyrosine residues on the yeast surface.^40–42^ One recent example linked surface-displayed enzyme activity to a bond-breaking ester hydrolysis reaction,^43^ but this platform was tailored to select for esterases that act on polymer substrates. Therefore, we needed to develop a new platform for yeast display evolution of enzymes for bioorthogonal protecting group unmasking.

We investigated whether masked acyl chloride probe labeling could be used to deposit a fluorescent label onto individual yeast cells displaying active enzyme variants. We used the enzyme BS2 esterase along with the probe AC-2, a masked acid chloride featuring the mCP protecting group. BS2 esterase is highly active toward the mCP protecting group,^25^ and BS2 together with the AC-2 probe was previously employed for spatially resolved tagging of RNA in living cells.^29^ We hypothesized that supplying the AC-2 probe to a dilute yeast cell suspension would enable spatially restricted tagging of yeast cells displaying active BS2 esterase, through covalent tagging of nucleophiles on the yeast surface (Figure 2a). Because acid chlorides are quenched rapidly in water,^44^ we hypothesized that neighboring yeast in the dilute cell suspension would be tagged minimally. We anticipated that our proposed ultrahigh-throughput cell surface display approach could yield bigger improvements in activity relative to previous efforts^25,33,45–48^ by using stringent selection conditions (i.e., short reaction times and low substrates concentrations) while screening large libraries (>10^7^).

**Figure 2:**
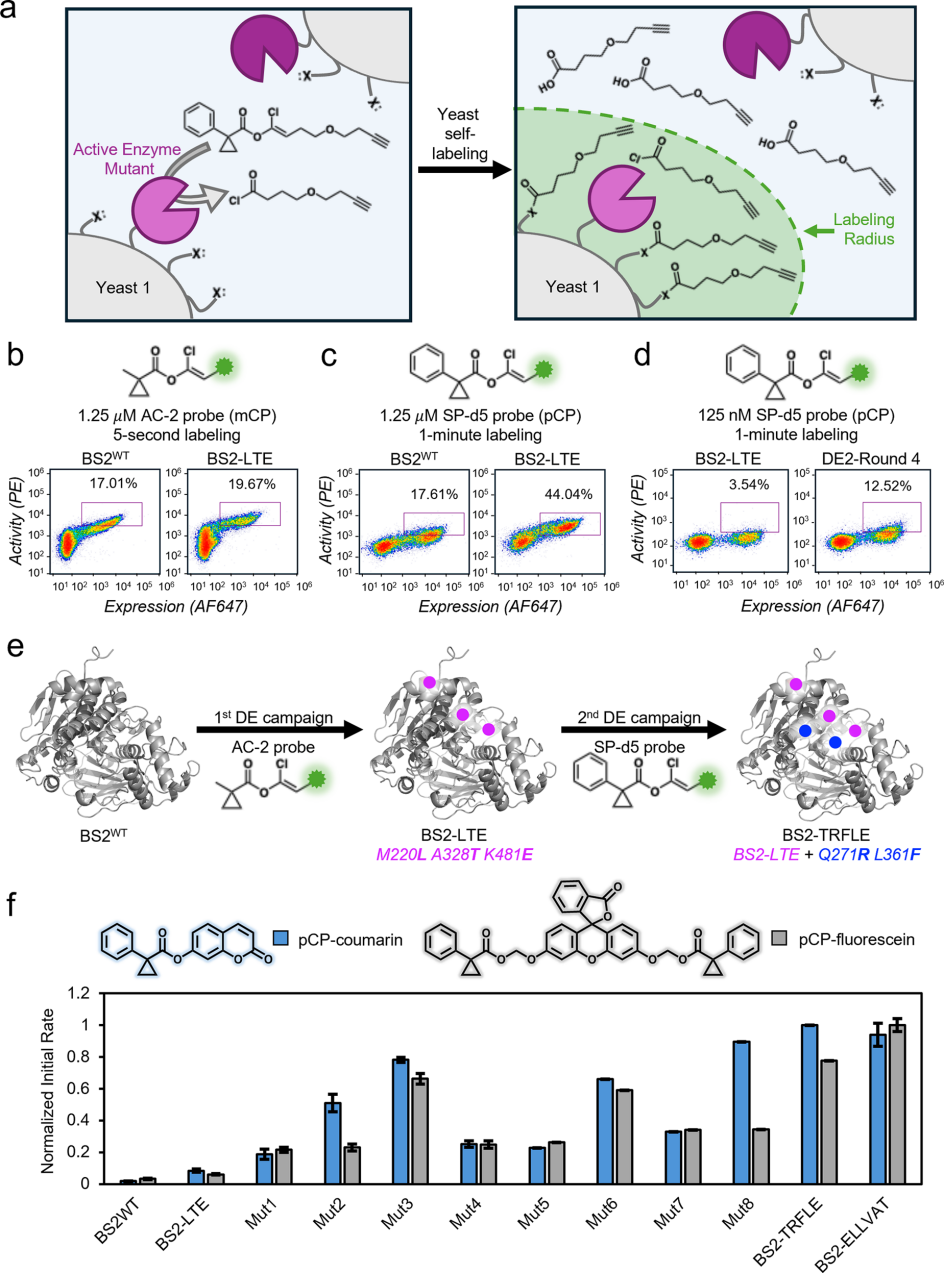
Platform development and directed evolution of BS2 esterase. (a) A library of yeast cells, consisting of >10^7^ yeast clones each displaying a distinct mutant, is labeled in a single tube to enable ultrahigh-throughput FACS. Active esterases cleave a masked acylating agent, which reacts with nearby nucleophiles on the cell surface. After a 5 s or 1 min labeling period with the indicated probe and concentration, esterases are quenched with PMSF. The alkyne moiety of the AC-2 or SP-d5 probe is then reacted with biotin-PEG_3_-azide via CuAAC and stained with sAv-PE (*y*-axis) while the myc tag is stained to detect expression (*x*-axis). (b) Flow cytometry comparison of activity of BS2^WT^ and BS2-LTE in monoclonal yeast populations labeled using 1.25 μM of AC-2, an mCP-containing probe. The purple box represents cells that expressed the enzyme and exhibited probe labeling higher than that seen in nonexpressing cells. In all flow cytometry plots, there is a substantial percentage of cells lacking myc expression, as is typical for the inducible expression system we used in this study.^40,43^ (c) Activity comparison of BS2^WT^ and BS2-LTE in monoclonal yeast using 1.25 μM SP-d5, a pCP-containing probe. (d) Flow cytometry analysis showing improvement in the second directed evolution (DE) campaign. Yeast samples consisted of monoclonal BS2-LTE or of an error-prone library of BS2-LTE mutants after 4 rounds of cell sorting. Cells were labeled using 125 nM SP-d5 probe. (e) Overview of the two DE campaigns. BS2^WT^ was evolved to create BS2-LTE using the mCP probe AC-2, followed by its evolution using the pCP probe SP-d5 to afford the final variant BS2-TRFLE. (f) Evaluation of activity of monoclonal yeast populations expressing BS2, BS2-LTE, and evolved variants of BS2-LTE against pCP-coumarin and pCP-fluorescein (AM ester). Esters were added at 20 μM to yeast suspensions, and the initial rate of fluorescence increase was quantified. Each experiment was performed in triplicate, and data are shown as the mean ± standard deviation. The data for each probe, pCP-coumarin and pCP-fluorescein, were normalized to the mutant with the highest activity for each probe, BS2-TRFLE and BS2-ELLVAT, respectively.

We cloned the BS2 gene into a yeast expression plasmid with C-terminal c-myc tag to enable detection of enzyme expression and subsequently transformed it into *S. cerevisiae*. The resultant yeast were incubated with AC-2, which is functionalized with an alkyne click handle. We then performed a copper-catalyzed Azide–Alkyne Coupling (CuAAC) reaction with biotin-PEG_3_-azide and stained with fluorescent streptavidin (sAv-PE) as well as anti-c-myc antibody to detect enzyme expression (Figure 2b). Using flow cytometry, we observed a strong correlation between labeling efficiency and expression, indicating that BS2 was expressed and active on the yeast cell surface (Figure 2b). The labeling solution volume and probe concentration were optimized to maximize the dynamic range (Supplementary Figure S10).

Having established the DEEPMACh platform, we tested whether it could be used to evolve BS2 for enhanced activity toward the mCP protecting group. We made an error-prone PCR library of the BS2 gene, and competent *S. cerevisiae* were transformed with the mutant genes to afford a 23-million member library with an average amino acid mutation rate of 4.1 per gene. This library was subjected to four rounds of labeling with AC-2 and sorting by FACS to isolate yeast with the highest ratio of activity to expression. We observed convergence upon variants with high expression and activity (Supplementary Figures 11–13 and Supplementary Spreadsheet 1). Sequencing of 49 post-round 4 clones revealed that the most prevalent mutations were T1A/P (14% of sequences), S153P (12% of sequences), and Y108H (10% of sequences). However, many of these mutations occurred together with myc tag mutations which caused the appearance of low expression and high activity (Supplementary Table 2). When considering only the mutants without myc tag mutations, four new mutations appeared in more than one sequence: K209R, M220L/K, F397S, and K481E. We evaluated the activity of these non-myc mutated variants in monoclonal yeast populations using a 5-s AC-2 labeling procedure. Several mutants exhibited a high ratio of activity to expression (Supplementary Figure 14), especially BS2 ^M220**L**/A328**T**/K481**E**^, which we call “BS2-LTE” (Figure 2b).

We next tested whether DEEPMACh could evolve BS2-LTE for activity toward the bulkier pCP protecting group, which shows superior bioorthogonality in mammalian cells. Replacing mCP with the pCP protecting group in masked acyl chloride probe labeling of BS2^WT^-expressing yeast greatly decreased yeast self-labeling, indicating that BS2^WT^ has much lower activity toward the pCP group (Figure 2c). Unexpectedly, BS2-LTE showed enhanced activity toward the pCP group compared to BS2^WT^ (Figure 2c), even though BS2-LTE was evolved on the mCP protecting group. We therefore used BS2-LTE as the starting point for evolution on the pCP protecting group. We created a mutagenic library of BS2-LTE using error-prone PCR and transformed it into *S. cerevisiae*. The resultant library had 27-million members with an average of 2.3 amino acid mutations per gene, in addition to the three mutations from BS2-LTE. Four rounds of sorting were completed using the SP-d5 probe, with each round revealing stronger sAv-PE signal relative to the unlabeled, nonexpressing population (Figure 2d and Supplementary Figures 15–17). Sequencing the round 4 yeast revealed several new mutations, including M212 V/I, P287L, and L361F, present in 32%, 24%, and 13% of the sequences, respectively (Figure 2e and Supplementary Spreadsheet 1). Additionally, all sequences retained the initial 3 BS2-LTE mutations with the exception of 16% of sequences adopting a M220P mutation in place of the original M220L mutation.

A panel of ten BS2-LTE mutants were selected for individual characterization in monoclonal yeast (Supplementary Table 3). All variants exhibited substantial improvement in activity toward the SP-d5 probe (Supplementary Figure 18). Excitingly, all variants also exhibited improved activity relative to BS2^WT^ when the pCP group was transferred to coumarin and fluorescein (Figure 2f). As a control, we tested all yeast suspensions on para-nitrophenyl butyrate (pnpB), an ester with a small *n*-propyl group. All variants including BS2^WT^ exhibited substantial activity toward pnpB (Supplementary Figure 19). Strikingly, all mutants exhibited a >20-fold increase in initial rate toward pCP-coumarin relative to BS2^WT^, with the most active variant exhibiting a 71-fold increase. Notably, the pCP coumarin and pCP fluorescein AM ester probes (Figure 2f) have very different structures from the SP-d5 probe, illustrating that the evolved variants exhibit enhanced activity toward pCP on diverse molecular structures. Two variants, “BS2-ELLVAT” (BS2^M220**L**/A328**T**/K481**E**/P287**L**/D319**A**/I470**V**^) and “BS2-TRFLE” (BS2^M220**L**/A328**T**/K481**E**/Q271**R**/L361**F**^), stood out based on their greatly improved initial rates, so we focused on BS2-TRFLE and BS2-ELLVAT for further characterization.

### Characterization of pCP-active BS2 Variants

To further investigate the activity of the top two variants, BS2-TRFLE and BS2-ELLVAT, we cloned, overexpressed, and purified them from *E. coli*. We tested the initial rates of hydrolysis for BS2^WT^, BS2-LTE, BS2-TRFLE, and BS2-ELLVAT, across a range of substrate concentrations for both mCP-coumarin and pCP-coumarin (Figure 3a–d and Supplementary Figures 20–35). BS2^WT^ was highly active toward mCP-coumarin, but far less active toward pCP-coumarin (140-fold lower *k*_cat_/K_M_). Consistent with yeast suspension assays, BS2-TRFLE and BS2-ELLVAT exhibited dramatically improved activity toward pCP-coumarin relative to BS2^WT^ (232- and 176-fold higher *k*_cat_/K_M_, respectively). The BS2-TRFLE mutant was also highly active toward mCP-coumarin (1.6-fold higher activity than BS2^WT^), while the BS2-ELLVAT mutant exhibited decreased activity toward mCP-coumarin (26% of the activity of BS2^WT^). BS2-LTE, which arose from the DE-1 campaign, exhibited 8.6-fold improvement toward pCP-coumarin relative to BS2^WT^, consistent with the yeast results showing improved activity toward the pCP protecting group, despite the fact that BS2-LTE arose from evolution on the mCP protecting group.

**Figure 3:**
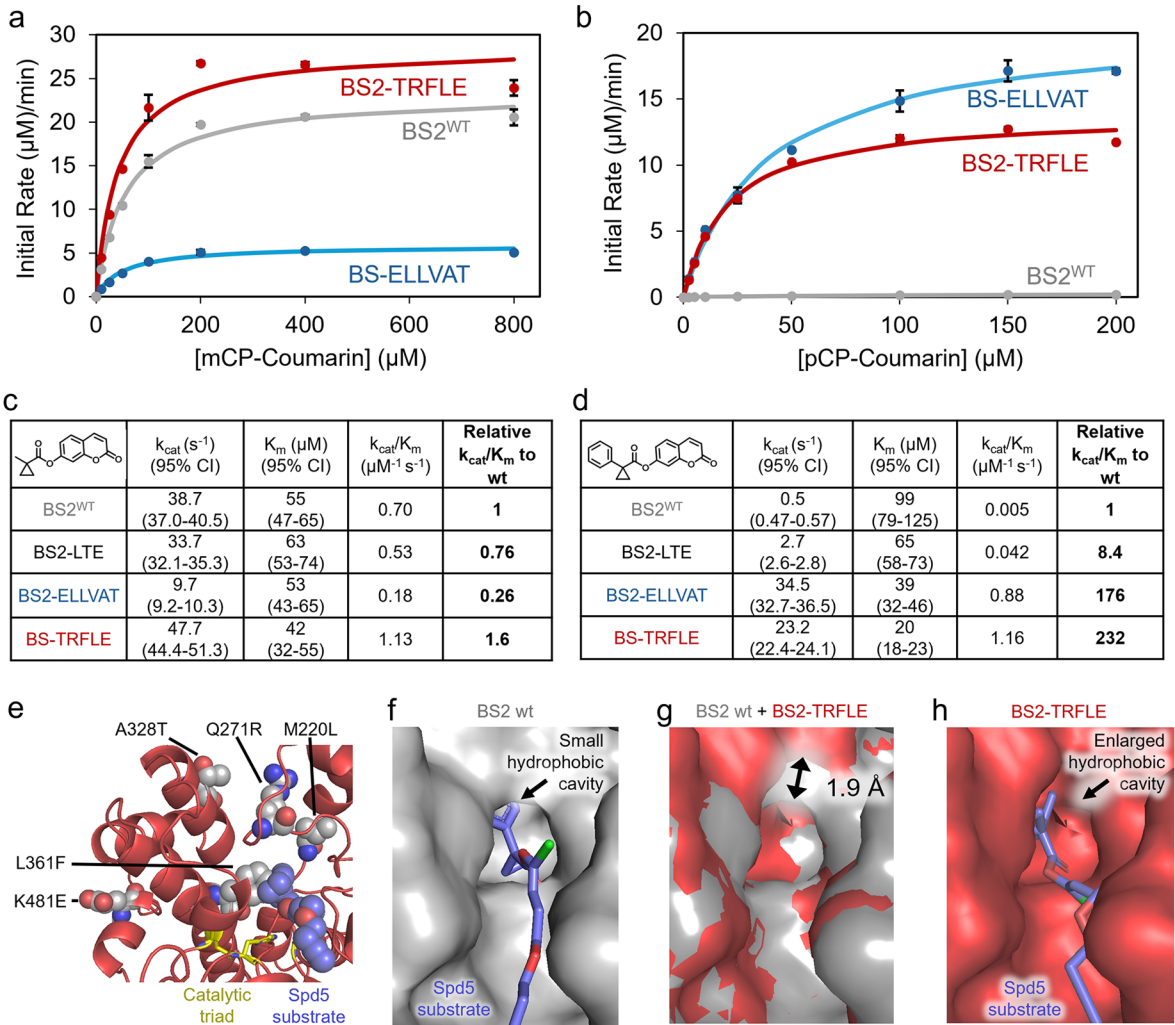
*In vitro* characterization of activity and structural modeling with probe docking for promising mutants. (a) Michaelis–Menten plot comparisons of BS2^WT^, BS2-ELLVAT, and BS2-TRFLE exhibiting the initial rate (μM/min) of mCP-coumarin hydrolysis with varying mCP-Coumarin concentrations (μM). Colored dots represent the average calculated initial rates of 3 independent *in vitro* reactions. Curves are the calculated best-fit Michaelis–Menten curves to the data. Black error bars represent the standard deviation from the average calculated initial rates from 3 independent *in vitro* reactions. Black error bars are present for all data points, but their visibility may be occluded by the data points. (b) Michaelis–Menten plot comparisons of BS2^WT^, BS2-ELLVAT, and BS2-TRFLE exhibiting the initial rate (μM/min) of pCP-coumarin hydrolysis as a function of pCP-coumarin concentration (μM). (c) Derived kinetic parameters for enzyme variants against mCP-coumarin. (d) Derived kinetic parameters for enzyme variants against pCP-coumarin. (e) AlphaFold3 structural prediction for BS2-TRFLE variant with docking of the SP-d5 substrate in the active site. The mutations in BS2-TRFLE relative to BS2^WT^ are depicted, along with the catalytic triad residues. (f) AlphaFold3 structural prediction for BS2^WT^ with docking of the SP-d5 substrate in the active site. (g) Overlay of AlphaFold3 structure predictions for BS2^WT^ and the BS2-TRFLE variant, illustrating that the opening to the hydrophobic cavity in the back of the active site has been enlarged by ∼1.9 Å in the BS2-TRFLE variant. (h) AlphaFold3 structural prediction for the BS2-TRFLE variant of BS2 with docking of the SP-d5 substrate in the active site.

To gain insights into why BS2-TRFLE and BS2-ELLVAT exhibit enhanced activity toward pCP substrates, we performed protein structure predictions and docking of the pCP-containing SP-d5 substrate (Figure 3e and Supplementary Figures 36–38). We used the AlphaFold3 web server^49^ to predict structures for BS2^WT^, BS2-TRFLE, and BS2-ELLVAT, and we applied Diffdock for substrate docking.^50^ The predicted BS2-TRFLE and BS2-ELLVAT structures featured an enlarged hydrophobic pocket at the back of the active site relative to BS2^WT^ (Figure 3f–h and Supplementary Figure 38). In BS2^WT^, key residues forming this pocket include M192, I269, L272, L361, and F362. Several of these residues, including M192 and F362, overlay closely with each other in all 3 calculated structures (BS2^WT^, BS2-TRFLE and BS2-ELLVAT), and the catalytic triad residues (S188, E309, and H398) also overlay well in all 3 structures. However, there is a striking difference in the positioning of I269, which forms the top of the hydrophobic cavity and which is shifted upward by ∼2 Å in both BS2-TRFLE and BS2-ELLVAT relative to BS2^WT^. We speculate that the wider opening of this hydrophobic cavity may contribute to the improved activity of BS2-TRFLE and BS2-ELLVAT toward the bulky pCP protecting group. Consistent with this possibility, we found that BS2-TRFLE and BS2-ELLVAT exhibit 8-fold and 4-fold higher activity, respectively, than wt BS2 toward a different bulky protecting group featuring two phenyl rings (Supplementary Figure 32), but they are similar to or less active than wt BS2 toward smaller protecting groups, including mCP esters, *n*-propyl esters, and acetyl substrates (Supplementary Figures 19 and 32). BS2-TRFLE and BS2-ELLVAT have the BS2-LTE mutations in common (M220L, A328T, K481E), which arose during the first evolution campaign, suggesting these three mutations may contribute to the expansion of the hydrophobic cavity. Additionally, BS2-TRFLE contains the L361F mutation in the hydrophobic cavity. Substrate docking suggests that L361F could potentially engage in a T-shaped π-stacking interaction with the phenyl ring of the pCP group (Figure 3e), which could contribute to the high activity of BS2-TRFLE toward pCP substrates. On the other hand, BS2-ELLVAT remarkably does not contain mutations at L361 or any residues expected to contact the substrate; all mutations are >10 Å from the substrate binding site. Thus, the dramatic improvement in BS2-ELLVAT toward the pCP protecting group, and its surprising 5-fold higher activity toward pCP relative to the smaller mCP group, can be attributed to distal mutations altering the shape of the active site pocket.

### pCP-Active BS2 Variants Are Active in Mammalian Cells

We next assessed the utility of the pCP protecting group and evolved BS2 mutants in living human cells. While the BS2 mutants were selected using DEEPMACh based on putative tagging of nucleophilic moieties on the surface of yeast cells, we reasoned that these variants would also be capable of proximity labeling inside living human cells, given the previous application of BS2^WT^ and the AC-2 probe for RNA proximity labeling in the BAP-seq method.^29^ We tested this hypothesis by labeling living mammalian cells using the pCP-masked acyl chloride probe, pAC-2, followed by microscopy to visualize the location of active enzyme and labeling events (Figure 4a). We began by expressing the best-performing pCP-active BS2 mutants in the nucleus of HEK293T cells, followed by pAC-2 labeling and quantitation of fluorescence intensity in the nucleus (Figure 4b and Supplementary Figure 39). While BS2^WT^ gave only a slight increase in signal compared to nontransfected cells, as expected based on the poor activity of BS2^WT^ toward pCP substrates, all of the evolved variants showed substantially higher fluorescence, with BS2-TRFLE and BS2-ELLVAT being the best (>6-fold increase relative to nontransfected).

**Figure 4:**
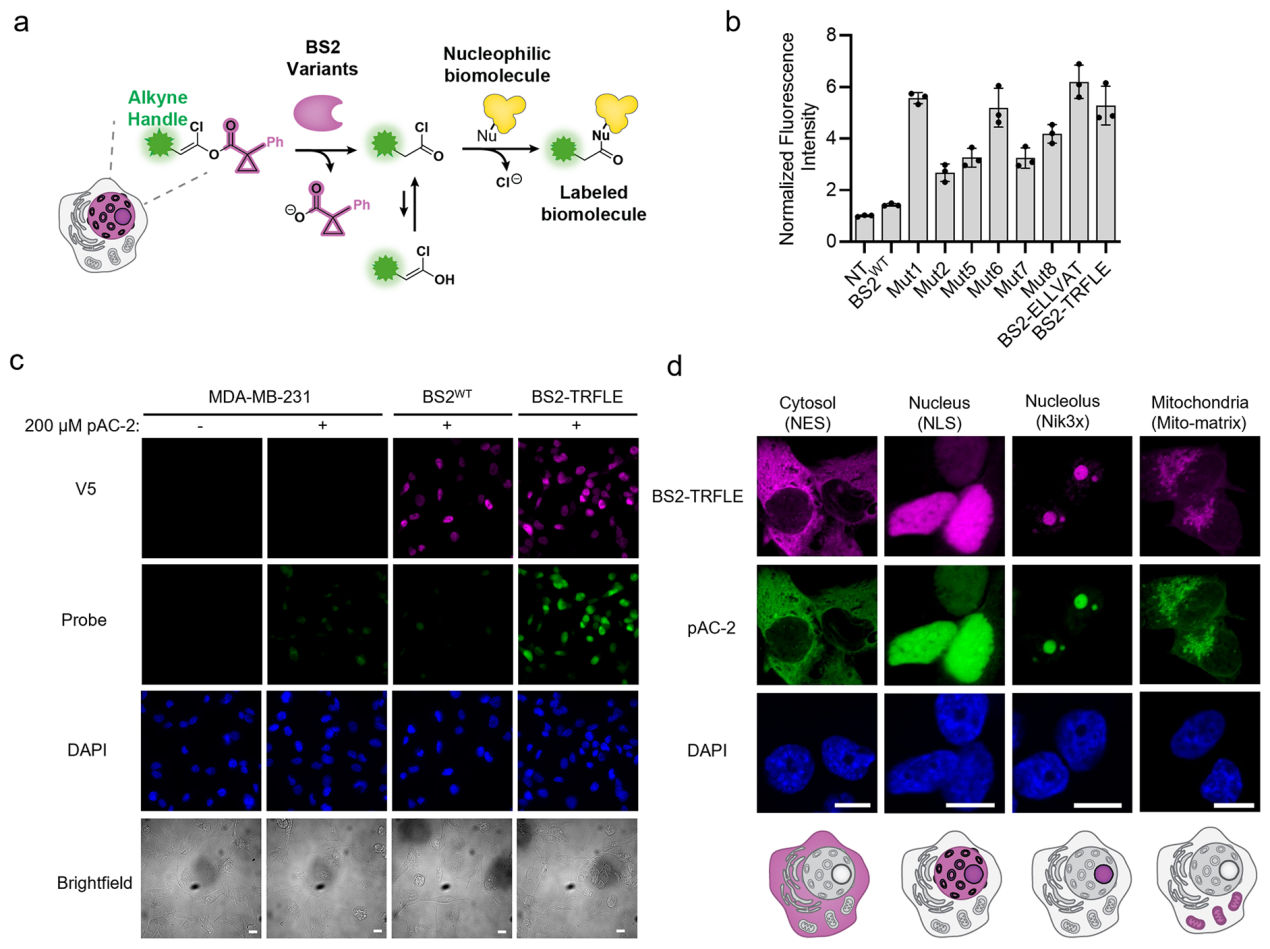
Unmasking of the pCP protecting group by evolved BS2 variants for acyl chloride labeling in living mammalian cells. (a) Schematic diagram showing pCP masked acylating probes processed by subcellularly localized BS2 variants, releasing acid chloride that covalently tags biomolecules in the vicinity. (b) pAC-2 labeling activity of top BS2 mutants in the nucleus of HEK293T cells. pAC-2 probe (100 μM) was added to the cells for 10 min at 37 °C. Labeling was measured by immunofluorescence imaging post click reaction with fluorophore AF488. Quantification of probe signal (AF488) in the nucleus is shown, gated on DAPI signal and normalized to the signal from nontransfected cells. *n* = 3 different fields of view. (c) Assessing pAC-2 labeling activity with BS2^WT^ and BS2-TRFLE. MDA-MB-231 cells stably expressing nuclear BS2^WT^ or BS2-TRFLE were treated with 200 μM pAC-2 probe for 5 min. Labeling was measured by widefield immunofluorescence imaging post click reaction with fluorophore AF488. DAPI is a nuclear marker. The scale bars on the brightfield images represent 10 μm. (d) MDA-MB-231 cells stably expressing BS2 in different cellular compartments were treated with 100 μM pAC-2 for 5 min. Cells were fixed, permeabilized, clicked with AF-488 for visualization. BS2-TRFLE was visualized through V5 antibody staining. The scale bars on the DAPI images represent 10 μm. The images are single z-slices obtained from confocal microscopy. The brightness of the images of each compartment is independently adjusted for optimum visualization.

We settled on BS2-TRFLE as the variant to use for subsequent experiments, as it showed better expression than BS2-ELLVAT in mammalian cells (Supplementary Figures 40 and 41). We compared the *in vitro* RNA labeling efficiencies of the pAC-2/BS2-TRFLE system with AC-2/BS2 ^29^ and APEX2-biotin-aniline.^51^ Under the conditions tested, the RNA labeling efficiency of the pAC-2/BS2-TRFLE system was similar to that of the AC-2/BS2^WT^ system, and higher than that of the APEX2/biotin-aniline system (Supplementary Figure 49). We further assessed the feasibility of proximity labeling in the nucleolus, a small membraneless compartment, using the new pAC-2/BS2-TRFLE system and compared it with the established APEX2-biotin phenol^52^ and APEX2-biotin-aniline.^51^ pAC-2 labeling in Hek293T cells expressing nucleolar BS2-TRFLE showed distinct nucleolar labeling compared to nucleoplasm, whereas both the APEX2 systems, under transient expression, showed poor distinction between nucleolus and nucleoplasm (Supplementary Figures 42–43 and 47). These results indicate that higher RNA labeling with masked acid chlorides is not due to loss of radial specificity, rather likely because of higher reactivity of acid-chlorides toward RNA compared to radicals under physiological conditions. We next generated BS2-TRFLE and BS2^WT^ stable cell lines in triple-negative breast cancer (TNBC) cells, which gave the highest background activity on mCP-fluorescein. Both enzymes were targeted to the nucleus via a nuclear localization signal (NLS) tag. We treated BS2-TRFLE -NLS stables, BS2^WT^-NLS stables, and noninfected MDA-MB-231 cells with 200 μM pAC-2 probe for 5 min and measured labeling intensity after a click reaction with AlexaFluor488. BS2^WT^ stables had negligible signal over background, similar to the results in HEK293T cells, reflecting poor enzymatic activity of BS2 on pCP substrates. Meanwhile, BS2-TRFLE stable cells showed significant labeling over background, highly colocalized with enzyme expression (Figure 4c and Supplementary Figure 44). To further assess the performance of the BS2-TRFLE/pAC-2 system, we generated cells stably expressing BS2-TRFLE in the cytosol, mitochondrial matrix, and nucleolus. Labeling with pAC-2 gave strong signal that colocalized well with enzyme expression in all the subcellular compartments, including the membraneless nucleolus compartment (Figure 4d and Supplementary Figures 45–48). These results establish that the evolved BS2 and pAC-2 pair can be applied in different subcellular compartments.

### Adapting the Evolved BS2/pAC-2 Pair to Map Subcellular Transcriptomes in Cell Lines with High Endogenous Background

After establishing that pAC-2 labeling was enzyme-dependent and colocalized with BS2-TRFLE within mammalian cells, we next tested whether this system with improved bioorthogonality could be applied for RNA proximity labeling (Figure 5a). We treated MDA-MB-231 cells stably expressing BS2-TRFLE with pAC-2 probe, followed by cell lysis, extraction of the total RNA, click attachment of AlexaFluor488, and visualization of tagged RNA via dot blot (Supplementary Figure 50). We saw significant RNA labeling in all compartments tested. We then proceeded to profile the labeled RNAs using high-throughput sequencing. We added the pAC-2 probe for 5 min to MDA-MB-231 cells expressing BS2-TRFLE in the cytosol, nucleus, or mitochondria. We extracted total RNA and enriched the labeled RNAs from ∼ 60 μg of input RNA per sample using streptavidin beads after performing a CuAAC reaction to attach biotin. We performed polyA capture on the enriched RNAs before proceeding to RNA-seq.

**Figure 5:**
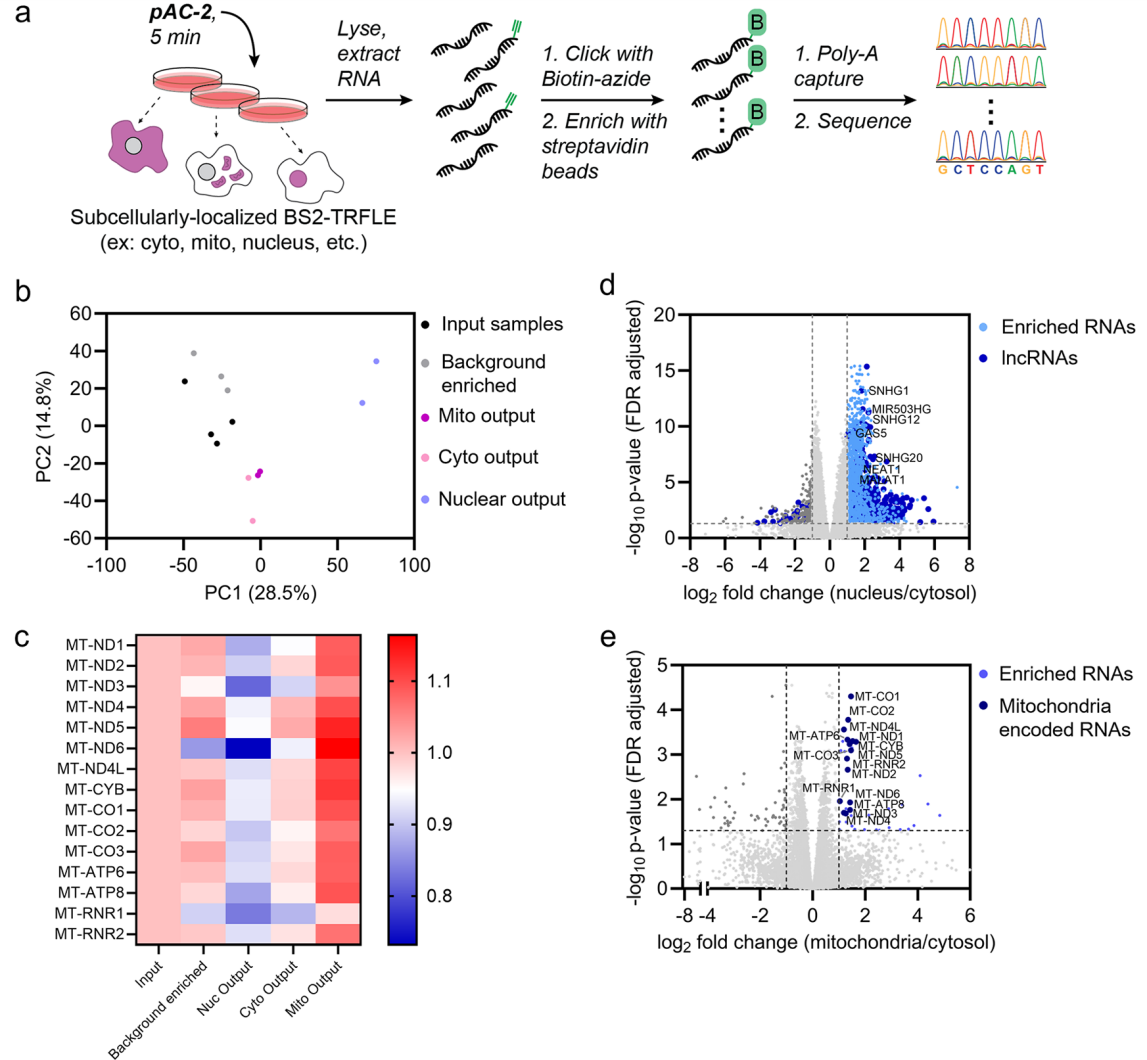
BS2-TRFLE-dependent proximity labeling of RNA using pAC-2 paired with quantitative sequencing provides a subcellular transcriptomic map in different compartments. (a) Schematic workflow of subcellular RNA mapping with pAC-2 and BS2-TRFLE. (b) Principal component analysis (PCA) of gene expression values for different samples with BS2-TRFLE/pAC-2 pair, *n* = 2–3 biological replicates. (c) Heatmap of average TMM normalized reads of mitochondria-encoded transcripts for different samples, *n* = 2–3 biological replicates. The reads of each transcript are normalized to that of input samples (input samples = 1). (d) Volcano plot depicting enrichment of nuclear transcripts from cells expressing nuclear BS2-TRFLE, compared with cells expressing BS2-TRFLE in the cytoplasm, with cut-offs of |log_2_(fold change)| > 1 and adjusted *p*-value <0.05. Common nuclear lncRNAs are highlighted. (e) Volcano plot depicting enrichment of mitochondrial transcripts from cells expressing mitochondrial BS2-TRFLE, compared with cells expressing BS2-TRFLE in the cytoplasm, with cut-offs of |log_2_(fold change)| > 1 and adjusted *p*-value <0.05. Known mitochondria-encoded RNAs are highlighted.

To assess the background labeling activity of the pAC-2 and AC-2 probes in MDA-MB-231 cells, we compared the background enriched transcripts to the input RNA samples. For AC-2 background enriched samples, we observed that 648 transcripts (3.6% of all observed transcripts) had higher than 2-fold normalized cpm (counts per million) reads compared to the corresponding input samples (Supplementary Figure 51). In comparison, pAC-2 background enriched samples had only 295 transcripts (1.7% of total) with higher than 2-fold higher cpm reads than their input counterparts. While some of the enriched transcripts in the background samples likely resulted from nonspecific binding to the streptavidin beads, the difference in the percentages of transcripts enriched over input for both the probes is likely due in part to lower background labeling for pAC-2, as observed in the imaging experiments (Figure 1f).

We investigated whether we had achieved subcompartment-specific RNA labeling using pAC-2 and BS2-TRFLE. Principal component analysis (PCA) showed distinct populations of enriched RNAs in different compartments, which widely differed from the input samples and background enriched RNAs (Figure 5b). We confirmed that stable BS2-TRFLE expression in different cellular compartments did not cause aberrant changes in the overall transcriptome profile (Supplementary Figure 54).

We next investigated what specific RNAs had been enriched in each compartment of TNBC cells. We looked for the cpm reads of mitochondria-encoded transcripts (mt-mRNAs, mt- rRNAs) across all compartments to assess the compartment-specificity of labeling. We were pleased to see that enriched samples from mitochondrial BS2-TRFLE, shown in purple, had the highest read counts for all mitochondrially encoded transcripts compared to other compartments (Figure 5c and Supplementary Spreadsheet 2), indicating good specificity for RNA labeling in the mitochondrial subcompartment.^52,53^ We then looked at the RNA population enriched in each compartment by comparing pairs of pAC-2 treated samples, each expressing BS2-TRFLE in a distinct compartment. The enriched RNA population was obtained after differentially expressed gene (DEG) analysis using Limma with cut-offs of log_2_fold change of 1 and *p*-value of 0.05. We observed significant enrichment of intronic species in the nucleus (∼20%) compared to other compartments (<10%), despite polyA capture that likely underrepresents the intronic species (Supplementary Figure 52). We found a total of 1664 RNAs enriched in the nucleus relative to the cytosol, out of which 516 were lncRNAs (long noncoding RNAs), including known nuclear-residing lncRNAs like Malat1, Neat1, Gas5, and SNHG12 (Figure 5d and Supplementary Spreadsheet 3). Of these 1664 nuclear transcripts identified in the TNBC cell line, 30% were common with the nuclear BAP-seq data set obtained in Hek293T cells using the AC-2/BS2 system,^29^ and 22% were common with the APEX2 nuclear data set^52^ obtained in Hek293T cells (Supplementary Figure 53). Meanwhile, the AC-2/BS2 Hek293T nuclear data set^29^ overlapped 5% with the APEX2 Hek293T nuclear data set.^52^ These findings indicate that each proximity labeling method likely detects a different subset of RNAs which is influenced by the inherent reactivity of the RNAs toward the probe and precise subcellular localization preferences of the enzymes.

We then compared the pAC-2 enriched transcripts in the mitochondria to that of cytosol and identified 39 transcripts enriched in the mitochondria, which included 13 mt-mRNAs and 2 mt-RNAs (Figure 5e). We also observed protein-coding transcripts IBA57, encoding for iron–sulfur cluster assembly factor, and PLA2G4B, encoding for phospholipase; both of their protein products are known to localize in mitochondria.^54^ The mitochondrial data set also contained several additional protein-coding transcripts: PLDB2, a senescence-associated transcript with a protein product that likely interacts with members of HIF-1 signaling pathway;^55^ FAM107A, whose protein product negatively regulates mitochondrial biogenesis;^56^ and FAM111B which is highly expressed in breast cancer, especially in TNBC, and is associated with poor prognosis.^57^ Taken together, these results show that the evolved BS2-pAC-2 protecting group can be applied for RNA proximity labeling and subcellular transcriptomics in biological systems, including ones that pose a high background challenge for mCP masked probes.

## Conclusion

Masking the functionality of molecular agents with bioorthogonal protecting groups and unmasking them with precise control is a powerful strategy to interrogate and manipulate biological systems. Achieving this goal requires protecting groups that are robust under biological conditions, yet readily unmasked under precisely controllable conditions. Bioorthogonality in this context often creates a conundrum: finding a molecule that is inert to the biological system of interest, while identifying an enzyme from another system that is capable of reacting with it. Mining natural sources for catalysts relies on a highly context-dependent view of bioorthogonality. Rather than mining natural biological sources for suitable catalysts, it would be preferable if we could identify the best bioorthogonal protecting groups, then evolve an enzyme specifically for that group.

To that end, here we have developed a new directed evolution platform, DEEPMACh, that allows the rapid discovery of new enzyme variants with enhanced activity for unmasking a bioorthogonal protecting group of interest. Our strategy combines yeast surface display with masked acylating probes, allowing a bond-breaking reaction (protecting group removal) to be linked to a bond-forming acylation reaction that deposits fluorescent labels on yeast displaying high-activity enzymes. Proximity labeling chemistry provides a powerful approach to connect genotype to phenotype in the context of enzyme evolution, enabling a pooled selection of high-activity enzyme variants from libraries of >10^7^.

In this study, we identified the pCP ester as a new protecting group with enhanced bioorthogonality in mammalian cells, relative to previously reported ester masks. However, BS2 esterase, the enzyme of choice for ester unmasking, is unable to efficiently process pCP-masked molecules. We used DEEPMACh to develop highly active BS2 enzymes with rapid kinetics for removing the pCP mask. In only two rounds of mutagenesis and selection, the DEEPMACh platform rapidly generated multiple enzyme variants with >230-fold enhancement in *k*_*cat*_/*K*_*M*_ for unmasking the pCP protecting group, including >70-fold enhancement in *k*_*cat*_. Thus, even if enzymes are not available from natural sources to remove a protecting group of interest, DEEPMACh can rapidly deliver enzymes with high activity. The ultrahigh-throughput afforded by the DEEPMACh platform allowed >40 million enzyme mutants to be screened in each of the two mutagenesis campaigns, resulting in multiple evolved variants, including ones in which all mutations were >10 Å from the substrate. DEEPMACh thus identifies mutations that conventional lower-throughput screening methods would be unlikely to discover. While the current study focuses on esterases, the DEEPMACh platform can in principle be expanded in future studies to diverse classes of hydrolase enzymes beyond esterases, and potentially even to nonhydrolase enzymes that have been previously applied for probe unmasking.^22,23^

The enhanced bioorthogonality of the pCP group allowed us to perform spatially resolved RNA tagging and sequencing in TNBC cells, representing a stringent test for the biological application of the system. These results will unlock wider application of the BAP-seq RNA proximity labeling method^29^ in biological systems requiring more biorthogonal probes. As in the original BAP-seq method, RNA proximity labeling using pCP-masked probes introduces the possibility of cellular perturbation arising from the tagging of nucleophilic biomolecules by acid chlorides, but the fast kinetics of the evolved BS2-TRFLE variant allows for short labeling times (3–5 min followed immediately by cell lysis), which mitigates the potential for physiological perturbation. The short labeling window also indicates that the pAC-2 masked acyl chloride probe diffuses rapidly into multiple subcellular compartments in the context of cultured cell monolayers. It is important to note that the evolved esterases BS2-TRFLE and BS2-ELLVAT exhibit substantial activity toward all the esters tested in this study, including mCP esters, *n*-propyl esterase, and acetyl substrates. The promiscuous activity of BS2-TRFLE could potentially perturb cellular physiology in some contexts, although we saw no evidence of toxicity in our experiments. In future studies, we anticipate the DEEPMACh platform will enable the development of BS2 variants with high selectivity toward pCP esters relative to other ester functional groups commonly found in biological metabolites. Overall, we anticipate that the DEEPMACh platform will further expand the chemical repertoire available for bioorthogonal protecting group unmasking, paving the way for applications in targeted cargo delivery, prodrug release, and selective biomolecular modification.

## Supplementary Material









## Data Availability

All BAP-seq data are available in the Sequence Read Archive through accession number GSE316735.

## Notes

First DE campaign mutants without silent mutations (XLSX)
Data (CSV)
Data for enriched transcripts (XLSX)
Additional experimental details, materials, methods, spectral data for characterization of all compounds, plasmid information, mammalian cell imaging data, fluorogenic probe activity assays, flow cytometry analysis from yeast labeling experiments, raw data for Michaelis–Menten kinetics, Michaelis–Menten fits, gel electrophoresis of purified enzymes, enzyme structural models with docked substrates, *in vitro* RNA labeling experiments, and transcriptomic analyses (PDF)
