# Supplementary material for: Directed Evolution of Enzymes for Bioorthogonal Chemistry Using Acid Chloride Proximity Labeling
Source: ACS Cent Sci. 2026 Jan 23;12(2):185–96. doi: 10.1021/acscentsci.5c01746 (PMC12947558; doi:10.1021/acscentsci.5c01746)
Supplement: Supplementary file 4 [file oc5c01746_si_004.pdf]

Supplementary Information for

Directed Evolution of Enzymes for Bioorthogonal Chemistry Using Acid Chloride Proximity Labeling

Ashley N. Ogorek,†[1] Shubhashree Pani,†[2] Eli J. Mertick-Sykes,[1] Jelena Momirov,[2] Yichong Lao,[1] Fernando Banales Mejia,[2] Rachel S.T. Chan,[2] Xuhui Huang,[1] Bryan C. Dickinson,*[2,3] and Jeffrey D. Martell*[1,4]

†These authors contributed equally to this work and are listed alphabetically

*To whom correspondence should be addressed: dickinson@uchicago.edu and jdmartell@wisc.edu

[1]*Department of Chemistry, University of Wisconsin-Madison, Madison, WI, 53706, USA*
[2]*Department of Chemistry, University of Chicago, 5735 S. Ellis Ave., Chicago, IL 60637*
[3]*Chan Zuckerberg Biohub, Chicago, IL 60642*
[4]*Carbone Cancer Center, University of Wisconsin School of Medicine and Public Health, Madison, Wisconsin*

Materials and Methods …………………………………………………….. S2
Supplementary Tables …………………………………………………….. S12
Supplementary Figures …………………………………………………….. S15
Synthesis Methods and Characterization ………………………………………….. S65

MATERIALS AND METHODS

Synthesis Methods
The detailed synthetic methods for all the probes are provided at the end of the Supporting Information.

Platereader Assays for Stability of Masked Fluorescein and Coumarin Probes
200 μM masked coumarin or 20 μM masked fluorescein probes were incubated at 37 °C in respective buffers (10% FBS, 10% Goat serum, 2 mM glutathione, or PBS) containing 10% DMSO by volume. For completely unmasked fluorophore controls, the masked probes (10× concentration) were treated with 0.33 N NaOH for 5 min, quenched with equimolar HCl, and diluted with PBS to get 1× concentration. For cell lysate treatment, 80% cells from a confluent 10 cm dish of MDA-MB-231 cells were lysed with 400 μL of RIPA lysis buffer with E64, pepstatin, and bestatin protease inhibitors, avoiding covalent serine hydrolase inhibitors like PMSF. 50 μL of this lysate was added to 130 μL of buffer and 20 μL of 10× probe in DMSO to get a final volume of 200 μL. The samples were incubated at 37 °C for 4 hr. All conditions were plated in triplicate in a 96-well plate, each well containing 100 μL of sample. Fluorescence measurements (λ_{ex} = 320/20 nm; λ_{em} = 450/20 nm for coumarin probes and λ_{ex} = 494/20 nm; λ_{em} = 520/20 nm for fluorescein probes) were carried out using a Synergy Neo2 Hybrid Multi-Mode Reader (BioTek Instruments, Inc.) with a gain of 60.

Cloning of Plasmids
See Table S4 for a summary of all plasmids, including links to their full sequences and feature annotations. The yeast expression plasmid pCTCON2-Aga2P-BS2 was prepared using Gibson Assembly.[1] The pCTCON2 constructs encoding Aga2P fused to BS2 was designed as described previously,[2] with BS2 attached to the C-terminus of Aga2P via a long and flexible linker. The construct design was as follows:

EcoRI-Aga2P-HA-TEVs-19 aa linker-NheI-**BS2 variant**-3 aa linker-BamHI-myc-stop-XhoI

A parent pCTCON2 plasmid was linearized by double digestion with the restriction enzymes NheI and BamHI, and the BS2 Gibson insert was prepared by PCR amplification using 466-d0-V5-BS2-NLS as the template.[3] pCTCON2 plasmids containing BS2 mutants were obtained from extraction of DNA from yeast libraries after multiple rounds of sorting (see below). The BS2 d0-NLS plasmids were also prepared using Gibson assembly, using PCR to amplify the full plasmid backbone (lacking the insert region) and to amplify the BS2 insert. All other mammalian expression and lentivirus production plasmids were constructed by Gibson Assembly with PCR products generated using Q5 or Phusion DNA polymerases (New England Biolabs). Bacterial expression plasmids for BS2 and its variants were cloned using Gibson Assembly. The genes for both the wild-type and mutant BS2 esterases were obtained via PCR amplification from the d0-V5-BS2-NLS mammalian expression plasmids and were purified using a PCR purification kit (Omega Bio-tek, Inc.) to create the Gibson insert sequences. The insert was joined with a linearized pET28a vector that had been digested using the restriction enzymes NdeI and HindIII using Gibson Assembly[1] to create the bacterial expression plasmid pET28a-BS2.

General Materials and Methods for Directed Evolution

All chemicals, solvents and other reagents were obtained from commercial suppliers (Sigma Aldrich, ThermoFisher, TCI, Santa Cruz, NEB, DOT Scientific, US Biological) and used as received unless otherwise indicated. Phosphate buffered saline (PBS) was prepared as follows: 137 mM NaCl, 2.7 mM KCl, 10 mM Na_2HPO_4, 1.8 mM KH_2PO_4 in ultrapure water. For cell washing steps of yeast labeling experiments, 1 mg/mL bovine serum albumin (BSA) was added to PBS, referred to as PBS-B. *S. cerevisiae* strain BJ5465 was obtained from ATCC (product no. 208289, batch no. 70003929). Yeast culture media (YPD or SDCAA) and yeast induction media (SGCAA) were prepared following a published procedure.[4]

Yeast Cell Culture and Preparation of Monoclonal Yeast Displaying Individual BS2 Variants

Saccharomyces cerevisiae strain BJ5465 was cultured according to previously published protocols.[2,4] Cells were grown at 30 °C in yeast extract peptone dextrose (YPD) complete medium. BJ5465 yeast were first transformed with a YIP plasmid containing native Aga1P protein sequence that had been linearized by digestion with the restriction enzyme BsiWI for genomic incorporation according to an established procedure.[5–7] Aga1P-expressing yeast were then made competent for transformation using the Frozen E-Z Yeast Transformation II Kit, followed by transformation with the appropriate pCTCON2 plasmid using the same kit. Transformed cells containing the Trp1 gene were selected on SDCAA plates. Yeast cell culture and induction of pCTCON2 construct expression were performed as described previously.[8]

Generation of Error-Prone Libraries for Yeast Display

Libraries of BS2 and BS2-LTE were generated using error-prone PCR (epPCR), following previously published protocols.[2,4,8] 130 ng of pCTCON2 vector containing the wild-type BS2 gene or the variant BS2-LTE was used as the template for the first round of epPCR. The parent gene was amplified for 20 rounds with: 0.4 µM Con.2 forward and reverse primers (F: 5′-CTAGTGGTGGAGGAGGCTCTGGTGGAGGCGGTAGCGGAGGCGGAGGGTCGGCTAGC-3′, R: 5′-TATCAGATCTCGAGCTATTACAAGTCCTCTTCAGAAATAAGCTTTTGTTCGGATCC-3′),[8] 2 mM $MgCl_2$, 10 units of Taq polymerase (NEB), and 2 µM each of the mutagenic nucleotide analogs 8-oxo-2'-deoxyguanosine-5'-triphosphate (8-oxo-dGTP) and 6H,8H-3,4-dihydro-pyrimido(4,5-c)(1,2)oxazin-7-one-8-β-D-2'-deoxy-ribofuranoside-5'-triphosphate (dPTP) (Jena Bioscience). The error-prone BS2 insert libraries were then gel purified (QIAquick Gel Extraction Kit, Qiagen) and subjected to amplification for another 30 cycles under normal PCR conditions using the HomR forward and reverse primers (F: 5′ CAAGGTCTGCAGGCTAGTGGTGGAGGAGGCTCTGGTG-3′, R: 5′ -CTACACTGTTGTTATCAGATCTCGAGCTATTACAAGTC-3′).[8] The mutated gene libraries were combined with BamHI-NheI-linearized pCTCON2 vector backbone (4.3 µg insert:1.1 µg vector), and concentrated via ethanol precipitation. The combined DNA in 10 µL total nuclease-free water was electroporated into competent *S. cerevisiae* BJ5465 yeast using an Eppendorf Eporator. The electroporated yeast were rescued using 125 mL of SDCAA media supplemented with 100 units/mL penicillin, 100 µg/mL streptomycin and 50 µg/mL kanamycin for 2 days at 30 °C. Maximum library diversity was found to be 2.3×10^7 for the original BS2 library and 2.7×10^7 for the BS2-LTE library, as determined by removing a small portion of the library shortly after electroporation and plating dilutions of the cell suspension onto SDCAA plates. Sanger sequencing

of representative clones showed an average of 4.1 or 2.3 mutated amino acids per gene for original BS2 library and BS2-LTE library, respectively; see Sequencing of Sorted Libraries section below for details on plasmid extraction and sequencing.

Fluorescence Activated Cell Sorting
Flow cytometry analysis was performed using an Attune NxT V6 Flow Cytometer (ThermoFisher Scientific) equipped with 561 and 633 nm lasers and appropriate emission filters (585/16 for phycoerythrin, and 670/14 for AlexaFluor647). Cell sorting was performed using a FACSAria Cell Sorter (BD Biosciences) with 561 and 633 nm lasers and corresponding filters (582/15 for phycoerythrin, 660/20 for AlexaFluor647).

Directed Evolution and Yeast Cell Labeling Protocol
Below is a general procedure describing cell labeling for monoclonal BS2 and mutant-displaying yeast for analytical experiments, as well as the initial error-prone library and subsequent sorted libraries (rounds 1–4) for sorting experiments. Table S1 specifies the designated volumes used for each step depending on the scale of the experiment. Experiments with 60 million or fewer yeast were performed in a 1.5 mL Eppendorf tube, while experiments with more than 60 million yeast were performed in 5 mL Eppendorf tubes, or 15- or 50-mL conical tubes.

S. cerevisiae cells containing wild-type BS2, the original mutated BS2 or BS2-LTE library, or subsequently sorted libraries were cultured in SDCAA at 30 °C for 48 hours to grow to saturation, and then a culture volume sufficient to maintain ten-fold library diversity coverage was diluted 1:9 in SGCAA media to induce surface expression (resulting in a 1:9 ratio of SDCAA:SGCAA). The culture was allowed to grow for 18 hours to saturation. The entire culture was spun down for 3 minutes at 4300 × g at 24 °C. The supernatant was discarded, and the cells were washed twice with PBS pH 7.4. The OD600 was measured to determine cell density. An aliquot of cells equal to at least ten-fold over coverage of library was removed and spun down. From this point forward, samples in 1.5 mL Eppendorf tubes were spun down for 1 minute at 10,000 × g at 24 °C and samples in 5-, 15-, or 50-mL vessels were spun down for 3 minutes at 4300 × g at 24 °C. The cell pellet was resuspended in 500 µL per 30 million yeast of an SDCAA solution containing 1.25 mM or 125 nM labeling probe (either AC-2 or SP-d5) and immediately vortexed thoroughly to suspend yeast in labeling solution. The yeast media SDCAA was used as the solvent in order to introduce diverse nucleophiles into the solution, which we hypothesized might quench the acid chlorides and restrict their diffusion radius. Incubation time was initiated upon vortexing, and yeast were incubated with acylating probe for 5 seconds or 1 minute on an orbital shaker with 180 rpm shaking. After labeling, the esterase was inactivated by addition of 5 µL of 100 mM PMSF in DMSO per 500 µL of labeling solution and the samples were vortexed again and incubated on the orbital shaker for an additional minute.

After quenching, the sample was spun down and the supernatant was removed. The cells were washed once with PBS-B pH 7.4 and once with PBS pH 7.4. After the second wash, the cell pellet was resuspended in a solution containing a CuAAC master mix with the following reagents: 5 mM sodium ascorbate, pre-complexed Cu:THPTA (prepared by combining 150 µL of 20 mM $CuSO_4$ in ultrapure water and 300 µL of 50 mM tris-hydroxypropyltriazolylmethylamine (THPTA) in DMSO) to final concentration of 0.150 mM Cu and 0.750 mM THPTA and 0.1 mM biotin-PEG_3-

azide.[9,10] The sample was vortexed to suspend yeast and placed horizontally on a rotating platform for 30 minutes at room temperature with 180 rpm rotation.

After the CuAAC reaction, the sample was spun down and the supernatant was removed. The cells were washed twice with PBS-B pH 7.4. Separately, a solution containing 1:100 µL anti-c-myc antibody (EMD Millipore Co., AB3253) in PBS-B pH 7.4 was prepared. Following the second wash the cell pellet was resuspended in the anti-c-myc antibody solution. The tube was set horizontally on a rotating platform to label for 30 minutes at room temperature. After 30 minutes, the sample was spun down and the supernatant was removed. The cells were washed twice with PBS-B pH 7.4. Separately, a solution containing 1:100 AlexaFluor™ 647 Goat anti-chicken IgY (H + L) (Invitrogen, A21449) and 1:100 streptavidin-phycoerythrin (sAv-PE, Invitrogen, S866) in PBS-B pH 7.4 was prepared and added to the washed pellet. The pellet was resuspended and placed horizontally on a rotating platform to label for 30 minutes at room temperature. The sample was spun down and the cells were washed twice with PBS-B pH 7.4.

After removing the last wash, the cells were then resuspended in PBS-B pH 7.4 to a density of approximately 60 million cell/mL and then passed through a 35 µm sterile filter. The solution was stored at 4 °C until it was analyzed on the FACS instrumentation. Gates were drawn to collect cells with positive AF647 and PE signal. Sorted cells were collected into a 15 mL culture tube coated with PBS-B and containing 1 mL of SDCAA media. Subsequently 9 mL of SDCAA was added as well as 1% penicillin-streptomycin, 50 µg/mL kanamycin and 10 µg/mL tetracycline. The cells were allowed to grow to saturation in SDCAA for 48 hours before being passaged for the next round of labeling and sorting.

Sequencing of Sorted Libraries

S. cerevisiae cells following the fourth round of sorting were grown in 5 mL of SDCAA media to saturation. 3.5 mL of culture was removed and centrifuged for 3 minutes at 4300 × g at room temperature, and the supernatant was discarded. Plasmids were extracted from the yeast using the Zymoprep Yeast Plasmid Miniprep I kit (Zymo Research) according to the manufacturer's instructions with the following modifications. The yeast pellet was suspended in 200 µL of solution 1 and 3 µL of Zymolase was added. The cells were incubated at 37 °C for 3 hours with 200 rpm shaking. After the 3 hour period, the samples were further diluted to 800 µL total by adding an additional 600 µL of solution 1. The sample was then split into four tubes, each containing 200 µL of sample, after which each of the four separate samples was processed following the manufacturer's instructions. The recovered plasmids were transformed into chemically competent DH5α *E. coli*. Monoclonal *E. coli* colonies from each plate were grown to saturation in 5 mL LB/amp media, then miniprepped using the Omega Bio-tek plasmid DNA purification kit (Omega Bio-tek Inc.) according to the manufacturer's instructions. Purified plasmids were submitted for Sanger sequencing. Sequencing data were analyzed using the program MUTATO.[11]

Fluorogenic and Colorimetric Activity Assays Using Suspensions of Yeast Displaying BS2 Variants

Yeast were induced via 1:10 dilution into SGCAA media at 30 °C for 18 hours. 5 mL cultures were spun down for 3 minutes at 4300 × g at room temperature and the supernatant was discarded. The yeast were washed twice with 2 mL of PBS. The OD600 was taken, and 30-million cell aliquots were removed, spun down for 1 minute at 10000 × g at room temperature, and suspended in 80 µL

of PBS. Separately, 100 µM solutions of pCP-coumarin (phenylcyclopropyl coumarin), pCP-FL (phenylcyclopropyl fluorescein), and pNPB (paranitrophenyl butyrate) were prepared in DMSO. 20 µL of 100 µM probe was then added to each well of a clear 96-well plate (Costar, 3370). Immediately preceding the fluorescence or absorbance measurements, 80 µL of yeast suspension was added to each well (final concentration of substrate was 20 µM), three wells at a time to measure hydrolysis of each substrate in triplicate, and the fluorescence was measured every 5 seconds for 85 seconds. Initial rate was calculated by determining the slope of fluorescence (RFU) or absorbance over time (seconds). Fluorescence measurements were recorded on a Tecan Spark fluorescence plate reader with the following conditions for coumarin derivatives: 350 nm excitation wavelength with a 7.5 nm bandwidth, 450 nm emission wavelength with a 7.5 nm emission bandwidth, 30 flashes with an integration time of 40 µs, and no delay time, gain of 100. The following conditions were used for fluorescein derivatives: 492 nm excitation wavelength with a 7.5 nm bandwidth, 520 nm emission wavelength with a 7.5 nm emission bandwidth, 30 flashes with an integration time of 40 µs, and no delay time with a gain of 75. Paranitrophenol was measured via absorbance at 350 nm with 10 flashes and a 50 ms settle time.

Overexpression and Purification of BS2 Mutants from *E. coli*

BL21 (DE3) *E. coli* cells were transformed with a pET28a bacterial overexpression plasmid containing either wild-type or a mutant BS2 gene. A single colony of transformed cells was inoculated in 5 mL of LB/Kan media at 37 °C overnight with shaking. All 5 mL of starter culture were inoculated into 500 mL of LB/Kan media in a 1000 mL baffled flask at 37 °C until an OD600 of 0.6–0.8 was reached. Expression of BS2 was induced with addition of IPTG to a final concentration of 0.2 mM, followed by incubation at 30 °C overnight (16–24 hours) with shaking. After the overexpression period, cultures were pelleted via centrifugation at 4000 × g at 4 °C for 30 minutes. Cell pellets were resuspended in BS2 lysis buffer (50 mM Tris-HCl, 100 mM NaCl, pH 7.5 (adjusted with HCl), 1 mg/mL Hen Egg White Lysozyme, 1 µL/mL 1 M $MgCl_2$, and 0.6 uL/mL DNase I (NEB)), placed on ice, and subsequently lysed via sonication (Fisher Scientific Fisherbrand Model 505 Sonic Dismembrator) with a ¼" probe at 30% amplitude for 10 min of on time (1 second on, 1 second off). Following sonication, the lysate was spun down to pellet the cell debris for 35 minutes at 4 °C at 22,000 × g in a fixed angle rotor centrifuge.

1–3 mL of Ni-NTA beads (GoldBio) were washed with 10 mL of BS2 purification buffer (50 mM Tris-HCl, 100 mM NaCl, adjusted to pH 7.5 with HCl) before the clarified lysate supernatant was transferred to the washed beads. The clarified lysate supernatant (mixed with resuspended Ni-NTA beads) was placed on ice for 30 minutes on an orbital shaker. After binding, clarified lysate supernatant was run through the column. The beads were then washed with at least 5 mL of BS2 purification buffer, followed by at least 15 mL of 30 mM imidazole BS2 purification buffer, and followed again by at least 15 mL of 60 mM imidazole BS2 purification buffer. The protein was eluted with 4 mL of 300 mM imidazole BS2 purification buffer. The elution buffer was exchanged into BS2 purification buffer by dialysis in 10,000 Da MW SnakeSkin Dialysis Tubing (ThermoFischer Scientific). For dialysis, the eluted protein solution was transferred to a dialysis tubing pouch and placed into 1 L of BS2 purification buffer for 1 hour then transferred to 1 L of fresh buffer for 2 hours before being transferred to an additional fresh 1 L of buffer and dialyzed overnight. Dialysis was performed at 4 °C. Enzyme solutions post-dialysis were then placed on ice and stored at 4 °C until further use. We determined that the specific activity of the purified wt BS2, BS2-LTE (A6 variant), BS2-ELLVAT (A3H variant), and BS2-TRFLE (C2L variant)

enzymes were unchanged within experimental error after 2 weeks of storage at 4 °C, at concentrations of 8 to 100 μM. All activity assays were performed within 2 weeks of purification.

Enzyme concentration was determined using the bicinchoninic acid (BCA) assay following the established literature protocol.[12] Protein purity was assessed using 12% SDS-PAGE. The gels were visualized via staining with Coomassie Brilliant Blue and destaining following the manufacturer's recommended protocol. Gels were imaged with an Azure C400 Gel Imaging System (Azure Biosystems) using the visible light setting.

***In Vitro* Kinetics**

The initial rates of mCP-coumarin hydrolysis by purified BS2 variants were determined by incubating varying concentrations of mCP-coumarin (50–800 μM) with 10 nM BS2 variant in Tris-HCl (pH 7.5; + 0.1 mg/mL BSA). The mCP-coumarin was delivered as a 10× stock solution in DMSO, leading to a concentration of 10% DMSO by volume in the reaction solution.

A varying concentration of pCP-coumarin (2.5–200 μM) was added to 10 nM BS2 in Tris-HCl (pH 7.5; + 0.1 mg/mL BSA) containing 10% DMSO by volume at room temperature. A standard curve of 0–50 μM 7-hydroxycoumarin was prepared by dissolving 10× concentrated stock solutions in DMSO (0.1–0.5 mM), followed by dilution with Tris-HCl (pH 7.5; + 0.1 mg/mL BSA) to the proper concentrations (10–50 μM) (Figure S20.). Reaction solutions were supplemented with BSA to improve the stability of BS2. Purified BS2 enzymes were stored at concentrations ranging from 8 to 100 μM without BSA supplementation, but when we diluted the enzymes to 10 nM concentration in the absence of BSA (for activity assays), we found that the activity was greatly reduced compared to when we performed the dilutions using buffer containing 0.1 mg/mL BSA (Figure S33). BSA supplementation has previously been used to decrease irreversible loss in enzyme activity for *in vitro* assays,[13] and it can prevent protein loss due to non-specific adsorption to plastic surfaces.[14] We confirmed that BSA supplementation did not lead to any detectable coumarin hydrolysis, and standard curves were prepared in the presence of BSA to account for any impact on fluorescence of the coumarin product (Figure S33). The accuracy of the mCP-coumarin substrate concentrations used in the assays were confirmed by plotting the 7-hydroxycoumarin standards alongside the equivalent concentrations of mCP-coumarin that were fully hydrolyzed by BS2 (Figure S21.).

All reactions were performed at 25 °C, and they were plated in triplicate in a 96-well plate. The kinetic measurement of BS2-catalyzed mCP-coumarin and pCP-coumarin hydrolysis were recorded using the Spark® multimode microplate reader (Tecan). The fluorescence measurements (λ_{ex} = 320/20 nm and λ_{em} = 450/20 nm) were recorded every 10 seconds for 20 minutes immediately upon addition of BS2 to mCP-coumarin or pCP-coumarin. Initial rates of mCP-coumarin and pCP-coumarin hydrolysis were obtained by converting the relative fluorescence units (RFU) of the first 5 data points to the concentration of 7-hydroxycoumarin in μM using the standard curve described above. Plotting of the initial rates in GraphPad Prism and fitting with a Michaelis-Menten curve allowed the derivation of the maximum velocity (V_{max}) and K_m. The kinetic parameter k_{cat} was obtained by dividing V_{max} by the total enzyme concentration (10 nM BS2 variant).

Structural Modeling and Substrate Docking

Structures for wt BS2, BS2-TRFLE, and BS2-ELLVAT were predicted using the AlphaFold3 web server,[15] with 20 models generated for each system. Figures 3e-h and S36-38 were prepared using the top-ranked AlphaFold3 structures, which revealed that BS2-TRFLE and BS2-ELLVAT were distinct from wt BS2 in their structures, in terms of the positioning of I269 (the top of the active site hydrophobic cavity). This difference was also observed when ensembles of predicted structures were considered, not just the top-ranked structures. Docking of the Spd5 substrate into the top-ranked AlphaFold3 structures was performed using DiffDock.[16] For each system, docking was conducted with 20 inference steps, 20 actual inference steps, and 20 samples. The binding pose with highest confidence score was selected for further analysis.

Mammalian Cell Culture, Plasmid Transfection, Lentivirus Preparation, and Stable Cell Line Generation

HEK293T, HeLa, MDA-MB-231 and MDA-MB-453 cells were obtained from ATCC. HepG2 cells were a gift from the Krishnan lab at UChicago. All cells were cultured in DMEM (GlutaMAX™, high glucose, sodium pyruvate, phenol red; Thermo Fisher) supplemented with 10% (vol/vol) fetal bovine serum (FBS, Gemini Benchmark) and 1% (vol/vol) penicillin/streptomycin (Gibco/Life Technologies). Cells were maintained in a water-saturated, 5% CO_2-containing 37°C incubator. Cells used for experiments never exceeded passage number 25. All cell lines were frozen at an early passage number (5 or less) in individual aliquots. There was no testing for Mycoplasma infection. Transient transfections were performed using Lipofectamine 3000 (Invitrogen; Thermo, L3000015) following the manufacturer's protocol. Lentivirus was prepared in Hek293T cells using the following protocol: Hek293T cells at 60% confluency in 10-cm dishes were transfected with 4 µg of 3rd generation lentiviral plasmid (cloned from lentiviral backbone-Addgene #52962 by replacing Cas9 with POI), containing corresponding genes of interest, along with 2 µg each of pMD2.G VSV-G envelope plasmid (Addgene #12259), pMDLg/pRRE 3rd generation packaging plasmid (Addgene #12251) and pRSV-Rev 3rd generation packaging plasmid (Addgene #12253). After 24 hr, the media was replaced with fresh media, and the old media containing viral particles was stored in 4 °C. Next day, the supernatant was collected and added to previously saved viral media, followed by a quick centrifugation and filtration through a 0.45 µm PES membrane to remove dead cells and cell debris. Then the viral solution was concentrated to ~0.5-1 mL volume using PEG-it Virus Precipitation Solution-5x (System Biosciences), aliquoted, and stored at -80 °C, avoiding multiple freeze-thaw cycles during future use. To generate MDA-MB-231 stable cell lines expressing BS2 in different compartments, 100 µL of concentrated lentiviral solution was added to each well of a 6-well plate containing MDA-MB-231 cells at 40-50% confluency. 48 hr later, the media was replaced with one containing 25 µg of blasticidin to start selection. Fresh media containing blasticidin was added every 48 hr for ~10 days until most cells died in the no lentivirus added control samples, and selection samples were ~90% confluent. Stable expression of BS2-mutant was confirmed via immunofluorescence imaging, and stable cells at early passage post-selection were frozen with 10% DMSO in liquid nitrogen for future use.

Fluorescence Microscopy

For epifluorescence microscopy, the samples were imaged on an inverted epifluorescence microscope (Leica DMi8) equipped with a Hamamatsu Orca-Flash 4.0 camera, a ×20 objective (for live-cell measurements) or ×63 oil objective (numerical aperture 1.4 for subcellular fixed-cell

imaging), and a 300 W Xenon light source (Sutter Lamda XL). Leica LASX software was used to obtain images for Alexa-488 (490/20x, ET Quad-S, ET 525/36m), Hoechst 33342 (ET 402/15×, Quad-S, ET 455/50 m), Alexa-555 (ET 555/25x, Quad-S, ET 605/52 m), and bright field. Acquisition time ranged from 30 to 800 ms. The acquisition time and Fluorescence Intensity Manager (FIM) were kept constant for a given channel across all samples that were compared in an experiment. For confocal microscopy, the slides were imaged on a Leica Stellaris 8 Laser Scanning Confocal microscope (DMI8-CS) equipped with near UV and white light laser (WLL). DAPI (HyD S1 detector, 405 nm laser excitation, 430/500 emission), Alexa-488 (HyD X2, 499 nm laser excitation, 510/560 nm emission), Alexa-555 (HyD S3 detector, WLL 80mHz, 558 nm laser excitation, 590/650 nm emission), BrightField (Trans PMT, WLL 80mHz) images were taken on 63x/1.4 UV oil objective with 3× magnification and either 4 or 8-line averaging on a single focal plane without z-stacking.

Live-Cell Imaging to Test Stability of Different Bulky Esters

8-chambered glass bottom dishes were coated with Poly-D-Lysine by incubating with 0.01 mg/mL of poly-D-Lysine in water for at least an hour at room temperature. Excess poly-D-lysine was washed off with Dulbecco's Phosphate Buffered Saline (DPBS). Respective cells (Hek293T, HEPG2, HeLa, MDA-MB-231, or MDA-MB-453) were plated on the coated 8-chambered plates in full media. The next day, the media was replaced with the corresponding ester masked fluorescein probe in PBS at 20 μM concentration and 1 μg/mL of Hoechst. The cells were incubated with the probes for 20 minutes at 37 °C, followed by a quick DPBS wash prior to imaging. At least 4 different fields of view were captured for each sample. Total fluorescence intensity was measured for the Alexa-488 channel and was normalized to the no-probe control for each cell line.

Immunofluorescence Imaging of Subcellular Compartments with Masked Acid Chloride Probes

Imaging experiments were performed using previously published protocols.[3] Briefly, MDA-MB-231 cells expressing BS2 or a BS2 mutant in different cellular compartments were plated on 24-well plates containing poly-D-lysine coated coverslips. After cells were ~80% confluent, the media was replaced with pAC-2 probe dissolved in pre-warmed DMEM. The cells were incubated with the probe solution for 5 min at 37 °C. Labeling was stopped with 1 mM PMSF solution. Cells were fixed with 4% paraformaldehyde solution, followed by permeabilization with ice-cold methanol. A click reaction was carried out for 1 hr at room temperature with 2 mM BTTAA (Click Chemistry Tools, 1236-100), 1 mM $CuSO_4$, 10 μM of AzDye 488 azide (CCT-1275, Vector Labs) and 10 mM sodium ascorbate (prepared fresh) in PBS. Samples were blocked with 3% BSA in PBS for 0.5-1 hr prior to overnight antibody incubation (Anti-V5, 1:2000; Invitrogen-R960-25). After 3 washes with PBS, secondary antibody (Anti-mouse AF555, 1:2000, abcam-ab150114) was added for 1 hr and washed thrice before mounting the coverslips on a glass slide. The samples were air-dried overnight in the dark and then imaged on the microscope.

Immunofluorescence Imaging of Subcellular Compartments with Biotin-Aniline and Biotin-Phenol

The imaging experiments were performed using published protocols.[17,18] Briefly, HEK293T cells were plated on a 24-well plate containing coverslips pretreated with 0.01 mg/mL of poly-D-Lysine for an hour at room temperature. Upon 70% confluency, cells were transfected with 500 ng of GFP-APEX2-Nik3x (Addgene #129274) using Lipofectamine 3000 reagent according to the

manufacturer's protocol. 24 hr post transfection, media was replaced with 500 µM of Biotin-Phenol or Biotin-Aniline and allowed to incubate at 37 °C for 30 min. Cells were washed once with DPBS, and then 1 mM H_2O_2 containing 500 µM of Biotin-Phenol or Biotin-Aniline was added to the cells for a minute with gentle shaking. Then the buffer was replaced with quencher solution (10 mM sodium ascorbate, 5 mM Trolox, and 10 mM sodium azide in DPBS) thrice. The cells were then fixed, permeabilized, stained with a Streptavidin-AF647 antibody and imaged with a widefield microscope.

RNA Dot-Blot to Assess RNA Labeling

An RNA dot-blot assay was carried out following a similar protocol as previously described[3] with the following modifications. MDA-MB-231 cells, stably expressing a BS2 mutant in a specific compartment, were labeled with 200 µM of pAC-2 for 5 min. After cell lysis and RNA extraction, a click reaction was performed with Alexafluor-488 at 25 °C for 30 min by adding 5× click mixture to get a final concentration of 0.4 mM $CuSO_4$, 0.5 mM AzDye 488 Azide (CCT-1275, Vector Labs), 1 mM THPTA (Click Chemistry Tools, 760952-88-3) and 10 mM sodium ascorbate along with 1 mM SUPERase•In RNase Inhibitor (Thermo Fisher, AM2694) in PBS. After column clean-up, ~5 µg RNA in ~ 5 µL water for each condition was dotted onto a Hybond membrane pre-soaked with 2× SSC buffer and air-dried. The blotted RNA on the membrane was allowed to dry at room temperature for 15 min before UV cross-linking at 2400 mJ/cm^2 (254 nm wavelength, UV Stratalinker 2400). The membrane was washed once with PBST (PBS buffer containing 0.05% Tween-20 v/v) and imaged on an Amersham Typhoon Laser Scanner in the Cy2 channel. For the loading control, the same membrane was stained with methylene blue, washed thrice with water, and imaged.

For *in vitro* RNA labeling, a solution was prepared in PBS containing 15 or 30 µg RNA (for 1 mg/mL and 2 mg/mL RNA concentration, respectively) along with 0.5 µM of BS2, BS2-TRFLE, or APEX2. Purified APEX2 enzyme was obtained and its concentration determined as described previously.[19] The respective probes (500 µM Biotin-Aniline or Biotin-phenol with 1 mM H_2O_2, or 100 µM AC-2 or pAC-2) were added last to get a final volume of 15 µL containing 10% DMSO by volume. All the samples were incubated on an orbital shaker at 37 °C at 900 rpm for 5 min. The reaction was then quenched with 1 mM PMSF for BAP-labeling samples and quencher solution (10 mM sodium ascorbate, 5 mM Trolox, and 10 mM sodium azide in DPBS) for APEX samples. RNAs were cleaned up with RNA Clean and Concentration (RCC) Kit and eluted in 8 µL water. The APEX samples were kept aside and BAP labelled samples were clicked with biotin with the following recipe: 0.4 mM $CuSO_4$, 2 mM biotin picolyl azide, 1 mM THPTA, and 10 mM sodium ascorbate along with 1mM SUPERase•In RNase Inhibitor in PBS for 30 min at room temperature (final volume 50 µL) and then cleaned up with the RCC kit. The concentrations of all the samples were checked with nanodrop. 4 µg RNA per sample was taken out and volume was adjusted to 5 µL. Next, PVDF membrane was cut to a desired size, and activated with methanol followed by 2x SSC buffer. The membrane was switched from Hybond to PVDF since Hybod showed a much higher non-specific streptavidin antibody background in these experiments. Once the SSC buffer dried out, 5 µL per sample was loaded on the membrane, air-dried and UV cross-linked at 2400 mJ/cm^2. The membrane was then washed once with PBST, followed by blocking with 3% BSA in TBST for 1 hr. The blocking buffer was then replaced with Streptavidin-HRP (#3999S, CST, 1:3000 in 3% BSA in TBST buffer), and the membrane was allowed to incubate overnight at 4 °C. The membrane was then washed with TBST thrice for 10 min per wash and imaged with

SuperSignal™ West Pico PLUS Chemiluminescent Substrate (#34577, Thermo Scientfic). The membrane was then stained with methylene blue, washed thrice with water, and imaged.

RNA-Seq Sample Preparation, Sequencing, and Data Analysis
Sample preparation for RNA-seq was carried out similarly to the BAP-seq protocol[3] with the following modifications. MDA-MB-231 cells and the respective stable cell lines expressing the BS2 variant in cytosol, nucleus, or mitochondria were plated in a 10 cm dish with 2-3 replicates per sample. After the cells reached 90% confluency, the samples were treated with 100 μM of pAC-2 in pre-warmed DMEM for 5 min at 37 °C. The labeling reaction was quenched with 1 mM PMSF solution in DPBS for a minute, followed by a quick DPBS wash. RNA was extracted with the Qiagen kit (Cat.# 74136), and a click reaction with biotin picolyl azide was carried out for ~60 μg RNA per replicate. The click reaction was carried out by incubating of the ~60 μg RNA with 0.4 mM $CuSO_4$, 2 mM biotin picolyl azide, 1 mM THPTA, and 10 mM sodium ascorbate along with 1mM SUPERase•In RNase Inhibitor in PBS for 30 min at room temperature (final volume 100 μL). 2 columns of RCC-25 kit (Zymo, R1018) were used to clean-up each sample of ~60 μg RNA, and the eluted RNA for each sample was later combined. After column purification, the enrichment of the biotinylated RNAs was carried out following the BAP-seq protocol[3] using 30 μL of Pierce Streptavidin Magnetic Beads. The enriched RNAs for all the samples (~1-2 ng, as measured by automated gel electrophoresis using an Agilent 2100 bioanalyzer instrument) were subjected to polyA capture. Subsequently, the DNA library with dual-index adapters was generated and amplified using the mRNA Hyper Prep Kit (Kapa Biosystems, KK8581) following the manufacturer's protocol. The amplification was carried out in 2 phases: the first amplification was a linear amplification with one primer - P7 Illumina Index primer (5'-CAA GCA GAA GAC GGC ATA CG*A-3'; * denotes phosphorothioate bond) at 2 μM final concentration for 16 cycles, followed by another 16 cycles after adding 2 μM of P5 primer (5'-AAT GAT ACG GCG ACC ACC G*A-3'). After amplification, the quality check of the library was performed with fragment analyzer. Libraries were sequenced on Illumina Novaseq X, 10B flowcell at ~20 million pair-end 300-cycle reads per library. The RNA-seq library generated was mapped to the *Homo sapiens* GRCh38 reference genome using STAR,[20] version 2.7.9a, and the aligned reads were quantified using Salmon[21] version 1.4.0. After read mapping with Salmon, the Bioconductor method[22] was used to read Salmon outputs into the R environment. Annotation data from Gencode version 38 were used to summarize data from the transcript level to the gene level. Filtering was performed to remove particular gene biotypes or genes with low expression. We first selected the gene biotype of interest: protein coding, lncRNA, Mt_rRNA, Mt_tRNA, rRNA, snRNA, scRNA and small nucleolar RNA and further filtered for low expression, keeping only genes with greater than 1 CPM. This reduced the number of genes from 60,708 to 16,777. To identify DEGs, precision weights were applied to trimmed mean of M-values-normalized gene counts based on within-group sample-level variance and gene-level mean-variance trends using VOOM.[23] The count data were fitted to a gene-wise linear model with group status as a coefficient, and the contrasts were specified for comparisons of interest. The Limma[24] empirical Bayes method was used to estimate the posterior odds of differential expression (|log(fold change)| >0) after adjusting for gene-level posterior residual standard deviations. Statistically significant DEGs were decided with |log2fold change| > 1 and a false discovery rate of 0.05. The results of differential expression testing are visualized by volcano plots comparing samples with BS2-TRFLE expression in two different locales (cytoplasm versus nucleus and cytoplasm versus mitochondria).

SUPPLEMENTARY TABLES

Table S1. Solution Volumes Used for Yeast Labeling Experiments

Sample:	General	Rd 1 Sort	Rd 1 BS2-LTE sort	Rd 2 sort*	Rd 3 and 4 sort*
Number of cells	3×10^7	6.21×10^8	1.38×10^9	1.2×10^8	3×10^7
PBS-B washes	200 µL	4 mL	9 mL	800 µL	200 µL
Acyl probe solution	500 µL	10.35 mL	23 mL	2 mL	500 µL
CuAAC solution	200 µL	4 mL	9 mL	800 µL	200 µL
Antibody and sAv-PE solution	50 µL	1 mL	2.3 mL	200 µL	50 µL
Final suspension volume	500 µL	12 mL	20 mL	2 mL	500 µL

*The number of cells and volumes of solutions for sorts 2–4 were the same for the original BS2 library (1st directed evolution campaign) and the BS2-LTE library (2nd directed evolution campaign).

Table S2. Selected Variants from Post-Round 4 of the First DE Campaign.

Mutant name	Mutations
A6 (BS2-LTE)	**M220L**, A328T, **K481E**
B5	N268S, I299L, **F397S**, I468V
C2	**Y108H**
C11	T1P**, Y108H**, E265A, **F397S**, I463T
C13	K30R, **K481E**
*C14**	N149D, **S153P**
C17	**M220K**, T228A, S445N

See Figure S11 for naming conventions. Bolded mutations are discussed in the main text and/or appeared in sequences lacking a myc tag mutation. *Mutant C14 had a mutation in the myc epitope tag.

Table S3. Selected Variants from Post-Round 4 of the Second DE Campaign

Main Text Mutant Name	SI Mutant Name	Mutations (in addition to A6 mutations: M220L, A328T, K481E)
Mut1	*A1H*	T1A, I59T, **M212V**
Mut2	*A2L*	T1A**, A196V**
BS2-ELLVAT	*A3H*	**P287L**, D319A, I470V
Mut3	*A5H*	**L361F**, E447G
Mut4	*B3H*	**M212V**
Mut5	*C1H*	W36C, Y108H, **M212I**, E286G
BS2-TRFLE	*C2L*	Q271R, **L361F**
Mut6	*C3H*	T1P, D49G, **M212I**, T219A
Mut7	*C5L*	T1P**, M212V,** E488G
Mut8	*D4H*	**L361F**, P403S

See Figure S15 for naming conventions. Bolded mutations were the most frequently occurring across all sequences.

Table S4. Plasmids Cloned in This Study

Plasmid Number	Benchling link	Description
BS2-01	https://benchling.com/shubhashree/f/lib_OeOlg9JE-bs2-variants/seq_NH8YKebk-copy-of-bs2-01-d0-v5-a3h-nls/edit	Mammalian expression vector with V5-A3H-NLS construct
BS2-02	https://benchling.com/shubhashree/f/lib_OeOlg9JE-bs2-variants/seq_4LIueZEe-copy-of-bs2-02-d0-v5-c1h-nls/edit	Mammalian expression vector with V5-C1H-NLS construct
BS2-03	https://benchling.com/shubhashree/f/lib_OeOlg9JE-bs2-variants/seq_F2qe3E6Y-copy-of-bs2-03d0-v5-d4h-nls/edit	Mammalian expression vector with V5-D4H-NLS construct
BS2-04	https://benchling.com/shubhashree/f/lib_OeOlg9JE-bs2-variants/seq_wLoTua3M-copy-of-bs2-04-d0-v5-a2l-nls/edit	Mammalian expression vector with V5-A2L-NLS construct
BS2-05	https://benchling.com/shubhashree/f/lib_OeOlg9JE-bs2-variants/seq_eLyBBH6n-copy-of-bs2-05-d0-v5-a1h-nls/edit	Mammalian expression vector with V5-A1H-NLS construct
BS2-06	https://benchling.com/shubhashree/f/lib_OeOlg9JE-bs2-variants/seq_TFFaYJ6a-copy-of-bs2-06-d0-v5-c3h-nls/edit	Mammalian expression vector with V5-C3H-NLS construct

BS2-07	https://benchling.com/shubhashree/f/lib_OeOlg9JE-bs2-variants/seq_7KwKsYEa-copy-of-bs2-07-d0-v5-c2l-nls/edit	Mammalian expression vector with V5-C2L-NLS construct
BS2-08	https://benchling.com/shubhashree/f/lib_OeOlg9JE-bs2-variants/seq_iATK4McG-copy-of-bs2-08d0-v5-c5l-nls/edit	Mammalian expression vector with V5-C5L-NLS construct
BS2-10	https://benchling.com/shubhashree/f/lib_OeOlg9JE-bs2-variants/seq_OgdshmJV-copy-of-bs2-10-d0-v5-a5h-nls/edit	Mammalian expression vector with V5-A5H-NLS construct
BS2-11	https://benchling.com/shubhashree/f/lib_OeOlg9JE-bs2-variants/seq_zEjTMrgi-copy-of-bs2-11-d0-v5-a6-nls/edit	Mammalian expression vector with V5-BS2-LTE-NLS construct
BS2-22	https://benchling.com/shubhashree/f/lib_OeOlg9JE-bs2-variants/seq_bimLwk3O-copy-of-bs2-22-lenti-v5-a3hbs2-nlsnucleoplasmin-p2a-blast/edit	Lentiviral vector expressing V5-A3H-NLS
BS2-23	https://benchling.com/shubhashree/f/lib_OeOlg9JE-bs2-variants/seq_Apy4OfuY-copy-of-bs2-23-lenti-v5-c2lbs2-nlsnucleoplasmin-p2a-blast/edit	Lentiviral vector expressing V5-C2L-NLS
BS2-31	https://benchling.com/shubhashree/f/lib_OeOlg9JE-bs2-variants/seq_iBlxj76a-copy-of-bs2-31-lenti-v5-c2lbs2-nes-p2a-blast/edit	Lentiviral vector expressing V5-C2L-NES
BS2-32	https://benchling.com/shubhashree/f/lib_OeOlg9JE-bs2-variants/seq_jQBSun81-copy-of-bs2-32-lenti-mito-v5-c2lbs2-p2a-blast/edit	Lentiviral vector expressing Mito-V5-C2L
BS2-33	https://benchling.com/shubhashree/f/lib_OeOlg9JE-bs2-variants/seq_bqiZd5RV-copy-of-bs2-33-lenti-v5-c2lbs2-nik3x-p2a-blast/edit	Lentiviral Vector expressing V5-C2L-Nik3x
BS2-34	https://benchling.com/s/seq-TfpHuSzachRMX7tptSZY?m=slm-kVl7i4Uflu19zt7wBRGN	Yeast Display Vector expressing aga2p-BS2-myc
BS2-35	https://benchling.com/s/seq-he2SvExjh5PjmafQTP9J?m=slm-myAoobroz0L5uar7YL7O	Bacterial Vector expressing wild-type His-tag-BS2
BS2-36	https://benchling.com/s/seq-vBPsXLYRC2sTqNDaSEz9?m=slm-QlRJFXvLFRSfk63rA8LY	Bacterial Vector expressing His-tag-BS2-LTE
BS2-37	https://benchling.com/s/seq-6i37HHNDKZoBTBJz7ngH?m=slm-G2ZQAsVfSxDHuRK01G6B	Bacterial Vector expressing His-tag-BS2-ELLVAT
BS2-38	https://benchling.com/s/seq-cps2L0n05mxJnEsdbRb4?m=slm-m0hdpFQXyFi7jJrQhLY5	Bacterial Vector expressing His-tag-BS2-TRFLE

SUPPLEMENTARY FIGURES

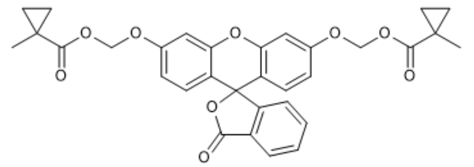

mCP-FL

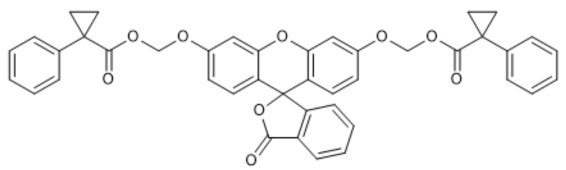

pCP-FL

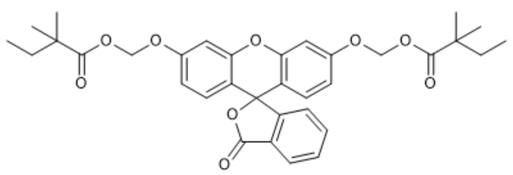

FBM108

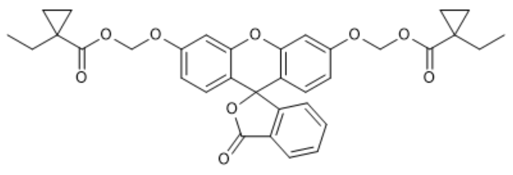

FBM112

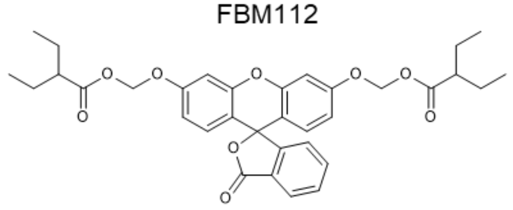

FBM114

Figure S1. Structures of all masked fluorescein esters tested.

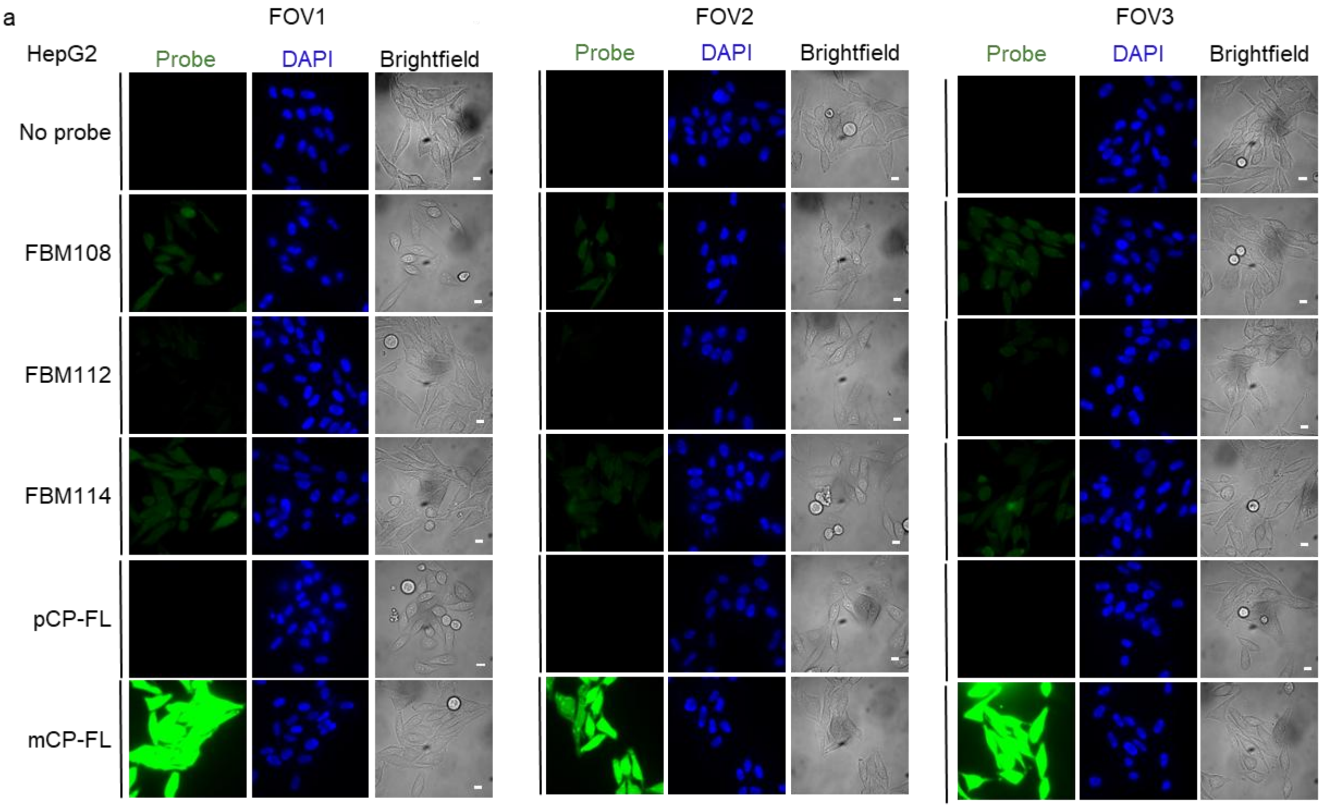

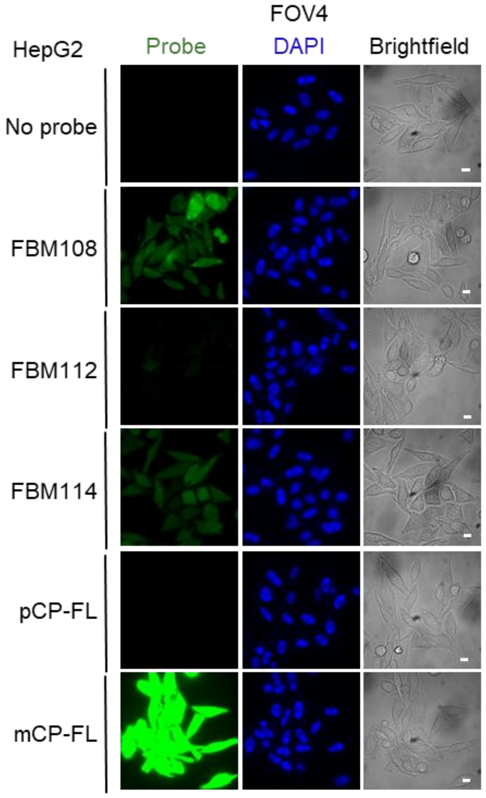

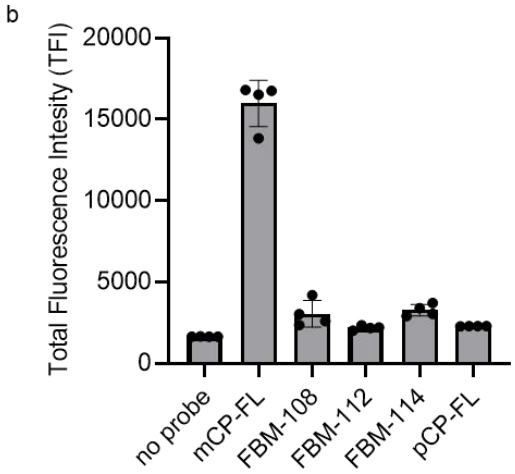

Figure S2. In cellulo activity of fluorescein probes monitored by fluorescence microscopy. a, Fluorescence imaging of five different fluorescein probes (20 μM) in HepG2 cells. Fluorescence activity was visualized after probe and DAPI incubation for 20 minutes at 37 °C. DAPI is a nuclear marker. The scale bar on brightfield images is 10 μm. b, Quantification of images shown in a. Total fluorescence intensity value of all replicates plotted, with error bars showing standard deviation, n=4 different fields of view (FOV).

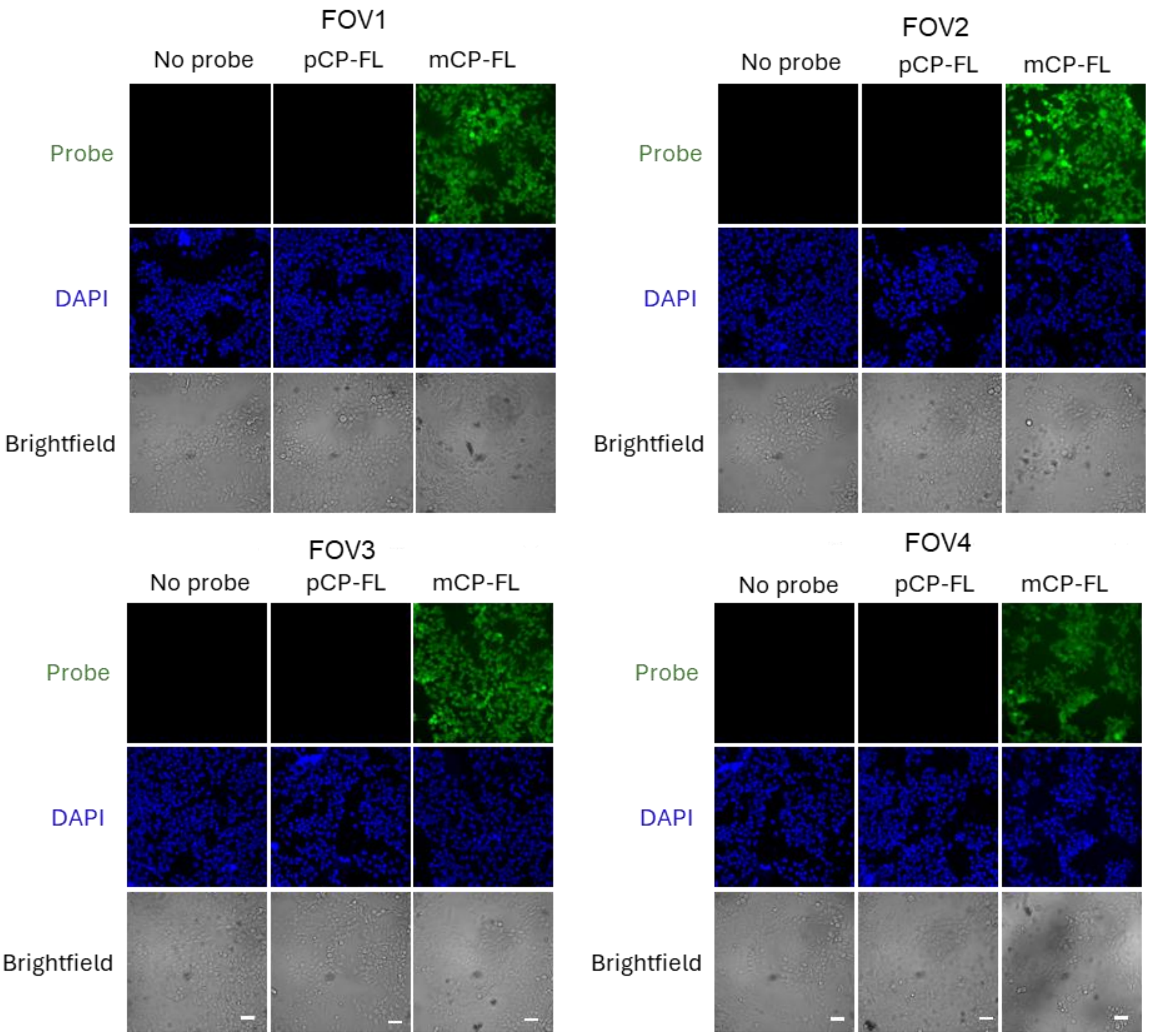

Figure S3. In cellulo activity of fluorescein probes monitored by fluorescence microscopy. a, Fluorescence imaging of mCP and pCP-fluorescein activity (20 μM) in HEK293T cells. Fluorescence activity was visualized after probe and DAPI incubation for 20 minutes at 37 °C. DAPI is a nuclear marker. The scale bar on brightfield images is 50 μm.

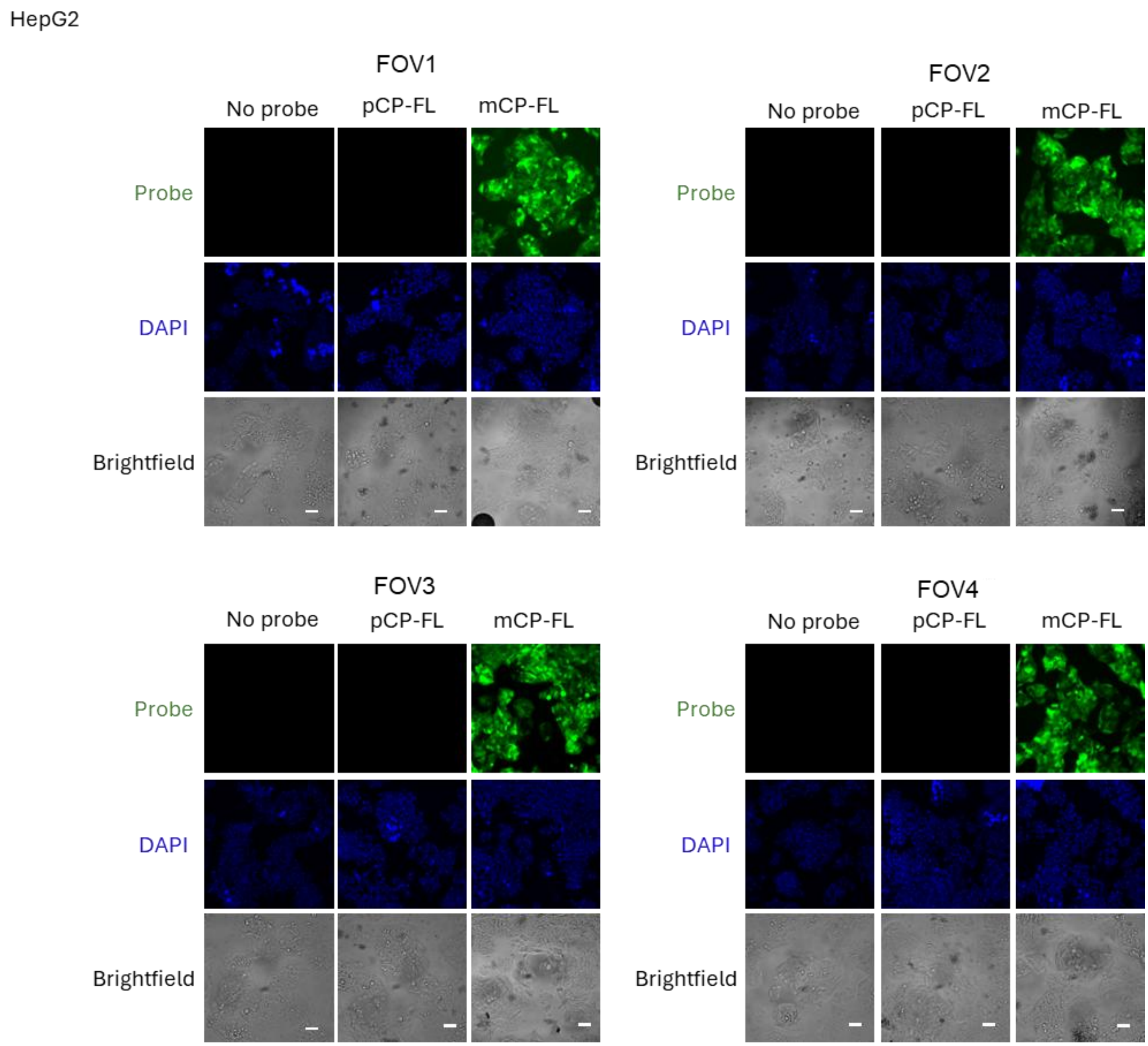

Figure S4. In cellulo activity of fluorescein probes monitored by fluorescence microscopy. a, Fluorescence imaging of mCP and pCP-fluorescein activity (20 μM) in HEPG2 cells. Fluorescence activity was visualized after probe and DAPI incubation for 20 minutes at 37 °C. DAPI is a nuclear marker. The scale bar on brightfield images is 50 μm.

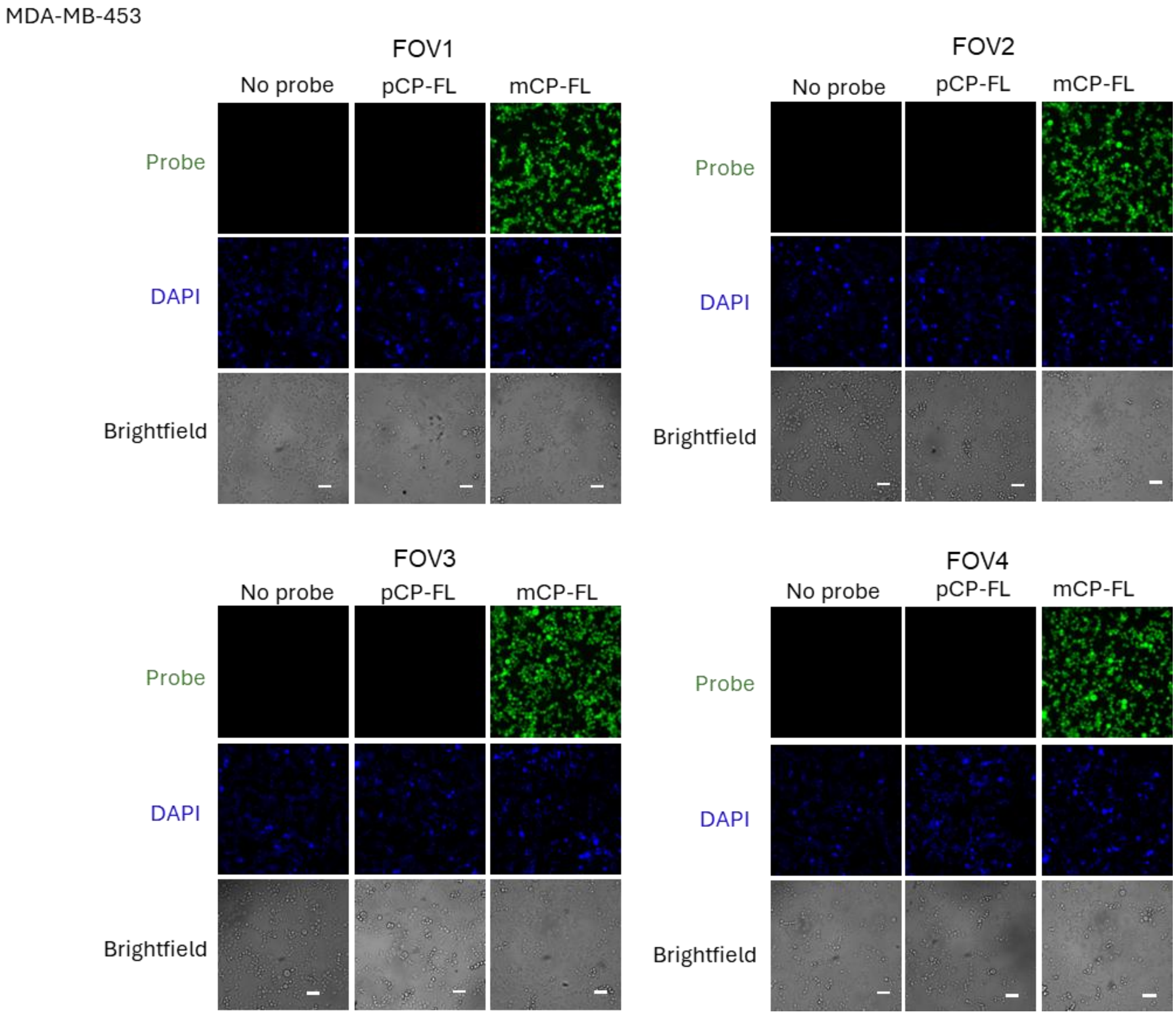

Figure S5. In cellulo activity of fluorescein probes monitored by fluorescence microscopy. a, Fluorescence imaging of mCP and pCP-fluorescein activity (20 μM) in MDA-MB-453 cells. Fluorescence activity was visualized after probe and DAPI incubation for 20 minutes at 37 °C. DAPI is a nuclear marker. The scale bar on brightfield images is 50 μm.

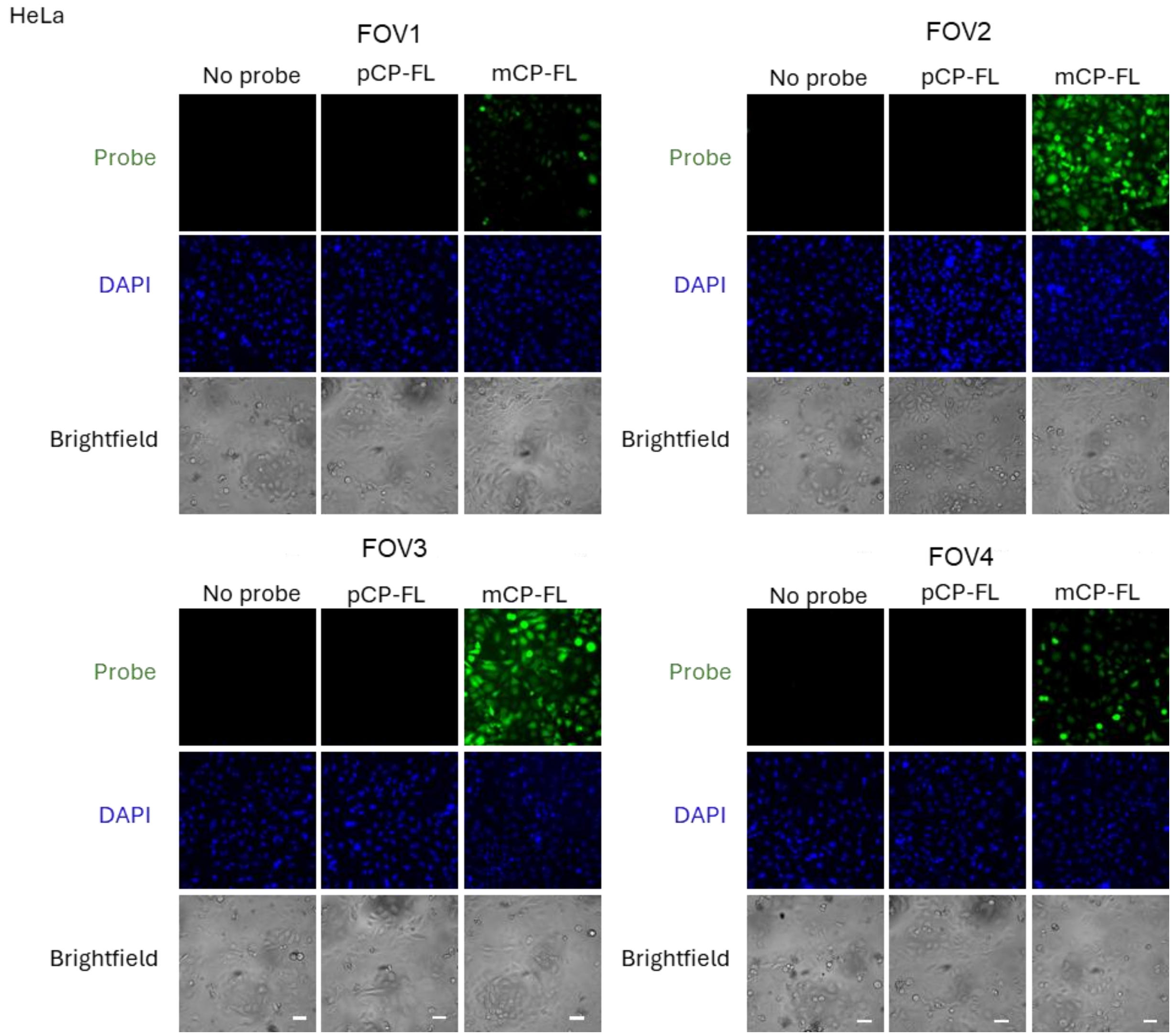

Figure S6. In cellulo activity of fluorescein probes monitored by fluorescence microscopy. a, Fluorescence imaging of mCP and pCP-fluorescein activity (20 μM) in HeLa cells. Fluorescence activity was visualized after probe and DAPI incubation for 20 minutes at 37 °C. DAPI is a nuclear marker. The scale bar on brightfield images is 50 μm.

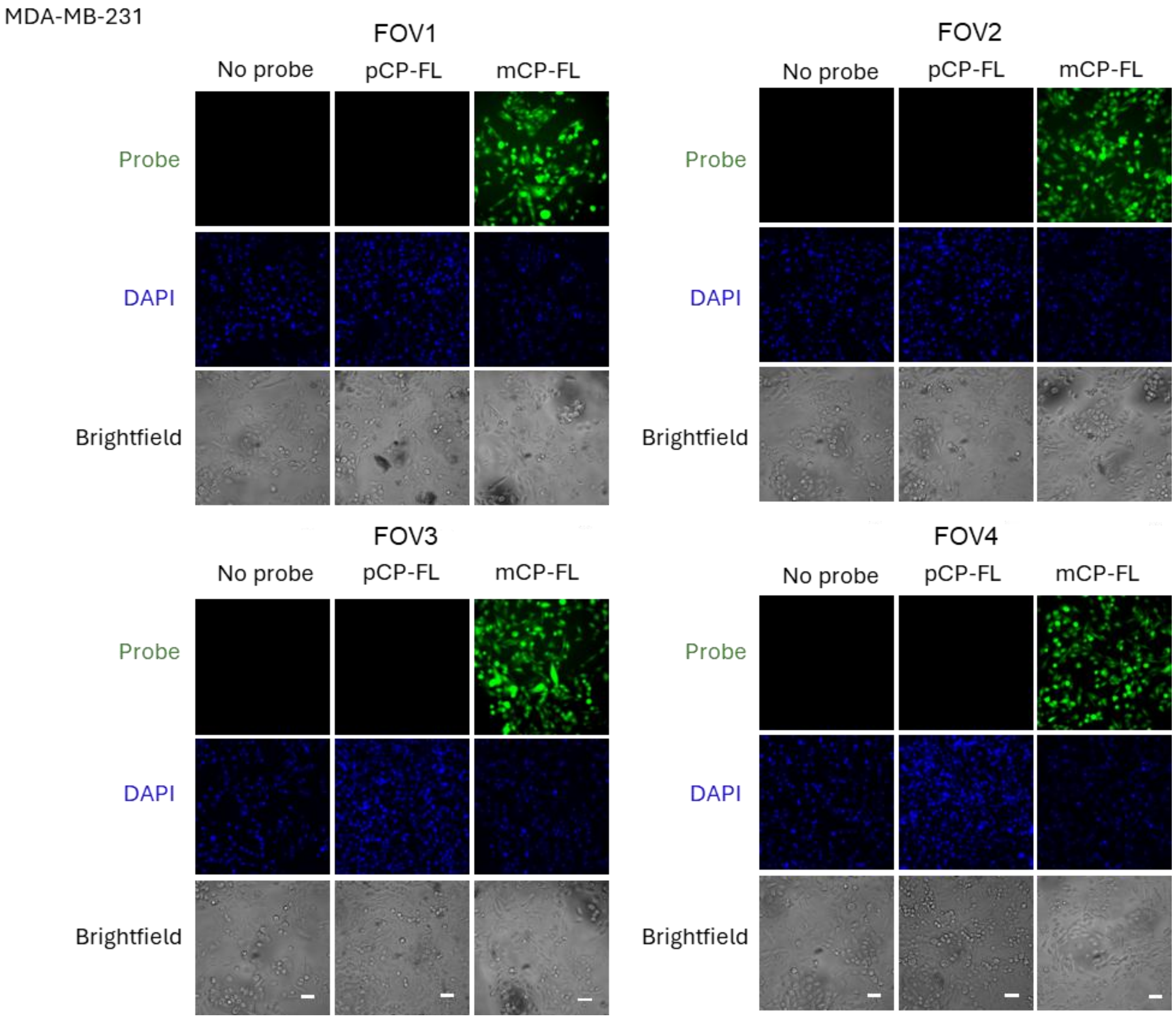

Figure S7. In cellulo activity of fluorescein probes monitored by fluorescence microscopy. a, Fluorescence imaging of mCP and pCP-fluorescein activity (20 µM) in MDA-MB-231 cells. Fluorescence activity was visualized after probe and DAPI incubation for 20 minutes at 37 °C. DAPI is a nuclear marker. The scale bar on brightfield images is 50 µm.

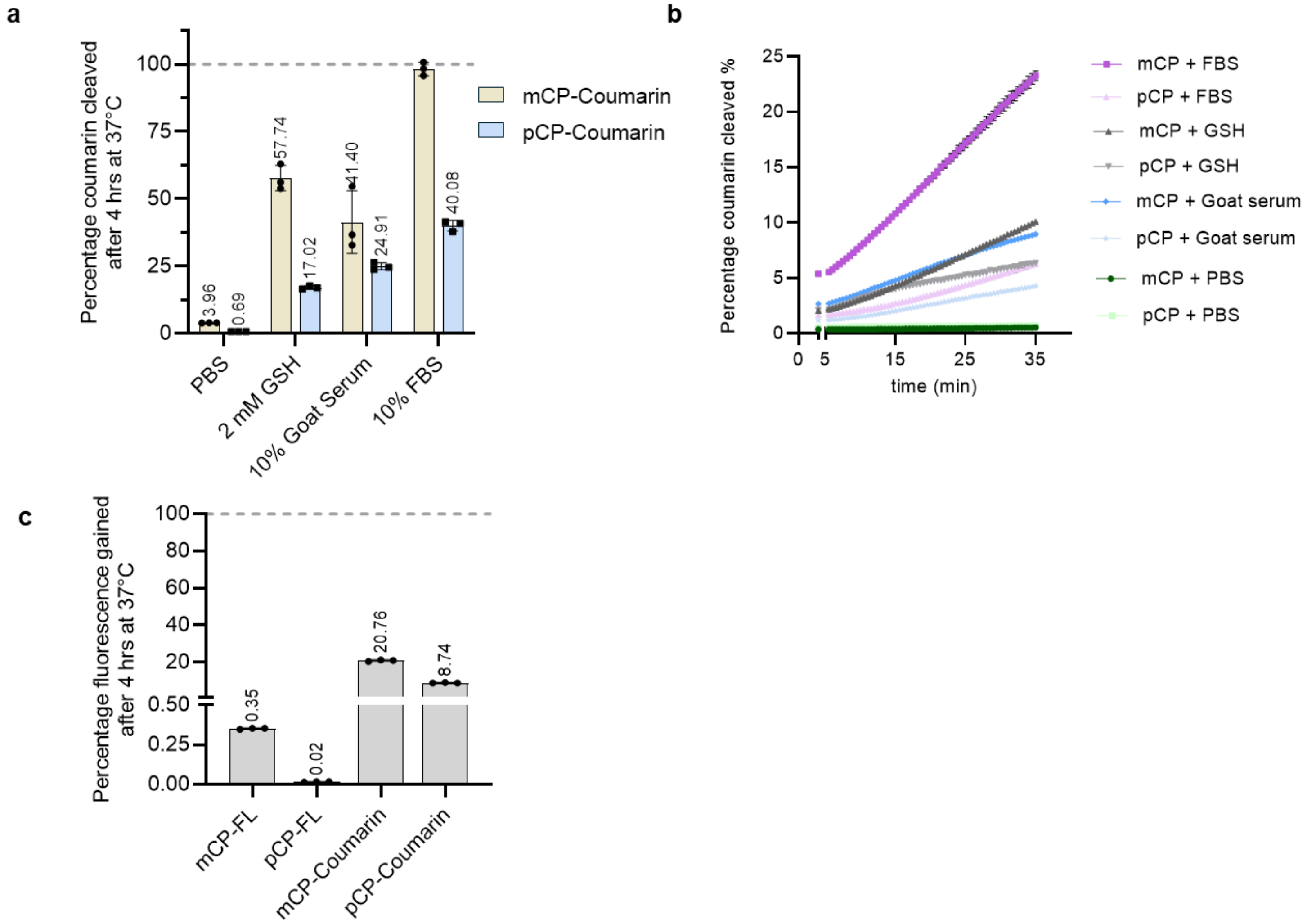

Figure S8. Comparison of stability profiles of mCP and pCP-coumarin in different biological contexts. **a)** The mCP and pCP coumarin probes were incubated in respective buffers at 37 °C, and fluorescence was measured after 4 hrs to assess the stability of the ester masks. The percentage of coumarin cleaved was assessed in comparison to the fluorescence from complete ester hydrolysis of respective probes with 0.33 N NaOH. **b)** mCP and pCP coumarin probes were incubated in the respective buffers for 5 min, and the ester unmasking was measured every 30 s for the next 30 minutes. The percentage of coumarin cleaved was assessed in comparison to the fluorescence from complete ester hydrolysis of respective probes with 0.5 N NaOH. **c)** mCP and pCP-coumarin (200 μM) and fluorescein probes (20 μM) were incubated with 25% (by volume) of cell lysate obtained from MDA-MB-231 cells in PBS for 4 hr at 37 °C. Fluorescence was measured separately for fluorescein and coumarin probes, and the percentage of fluorescence gained was assessed in comparison to fluorescence from complete ester hydrolysis of respective probes in respective concentrations with 0.33 N NaOH.

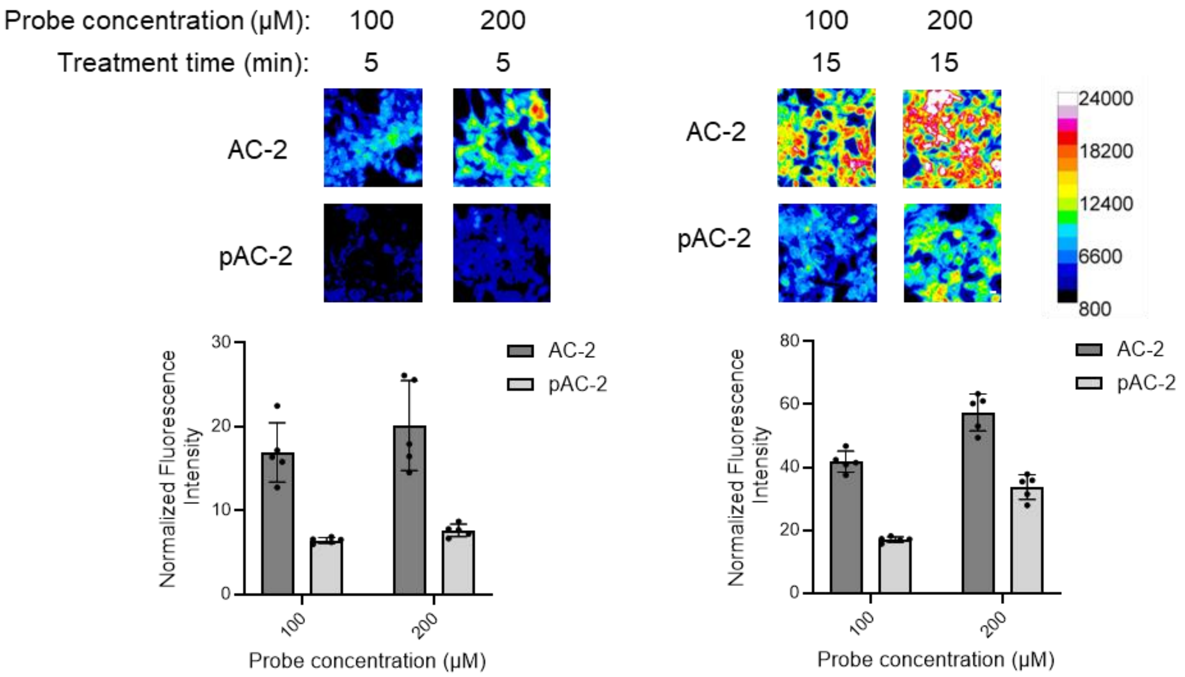

Figure S9. Background activity of masked acid chloride probes in HEK293T cells. Different concentrations of AC-2 and pAC-2 probes were incubated in HeK293T cells for 5 min, and background activity was measured by immunofluorescence imaging post click reaction with fluorophore AF488. Fluorescence signal from the samples without the probe treatment was used for normalization. The calibration bar on the right shows fluorescence units corresponding to the respective colors. Scale bar on the bottom right image represents 10 µm.

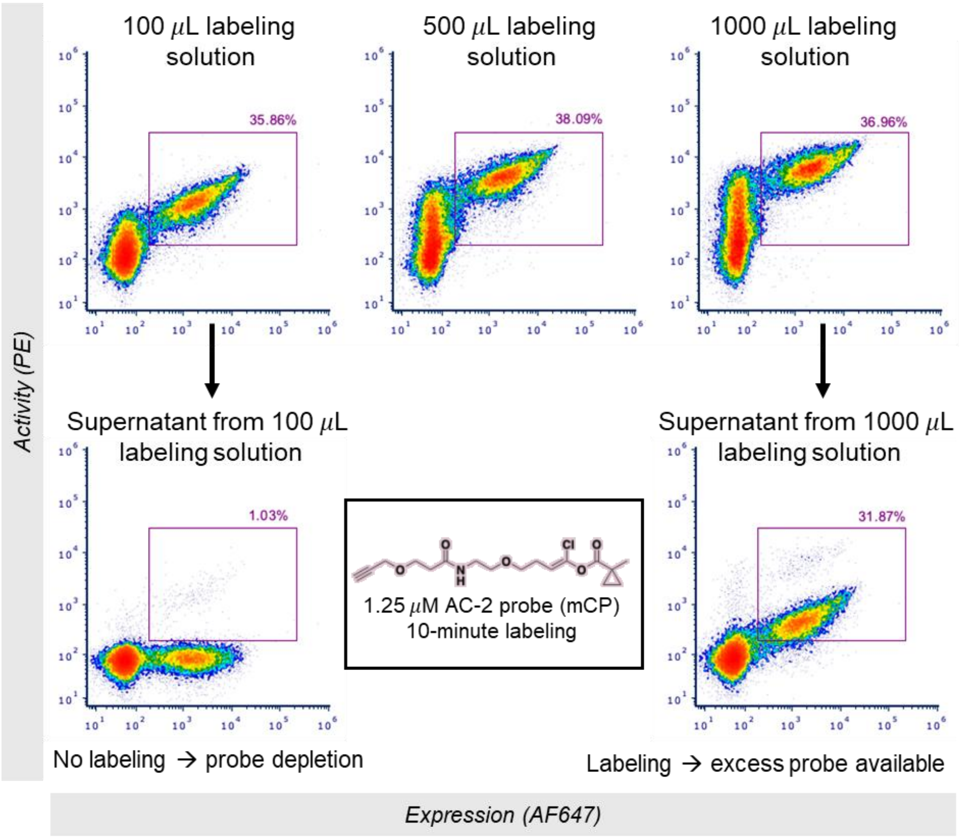

Figure S10. Optimization of BS2^{WT}-expressing yeast labeling with AC-2 probe. Induced yeast (30 million cells per sample) were exposed to 100, 500, or 1000 µL of 1.25 µM AC-2 labeling solution for 10 minutes before spinning the samples down and removing the supernatant (top row). The supernatant from the 100 and 1000 µL samples were then transferred to a fresh, unlabeled 30 million cell aliquot and incubated for 10 minutes (bottom row). AC-2 probe was reacted with biotin-PEG$_3$-Azide via CuAAC then stained with sAv-PE on the *y*-axis while the myc tag was stained with antibodies on the *x*-axis. The 500 µL sample afforded high signal while still retaining a steep slope correlating activity with expression and was chosen for subsequent experiments. Yeast cells not expressing the enzyme showed substantial *y*-axis signal under these conditions, indicating intercellular labeling, but the *y*-axis signal was higher for cells expression the enzyme. Additionally, when only 100 µL of labeling solution was used, we observed substrate depletion, which limits the dynamic range observable on the *y*-axis. However, when 1000 µL of labeling solution was used, the supernatant still had sufficient unreacted probe remaining to label a new sample of yeast in a different tube.

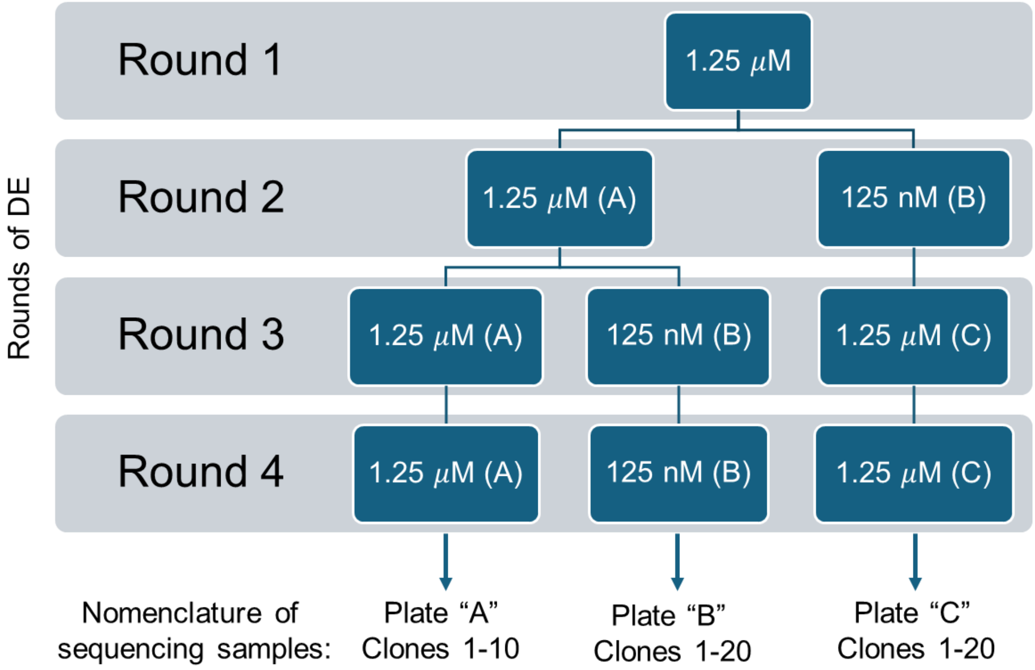

Figure S11. Flow chart demonstrating the parent populations and concentrations of AC-2 probe used for the first DE campaign. 125 nM probe was used as an alternative to 1.25 μM probe to test whether highly active variants in low probe concentrations could be identified. The nomenclature of the sequenced variants from DE 1 are also shown ("A/B/C" refer to which round 4 sort the clone came from).

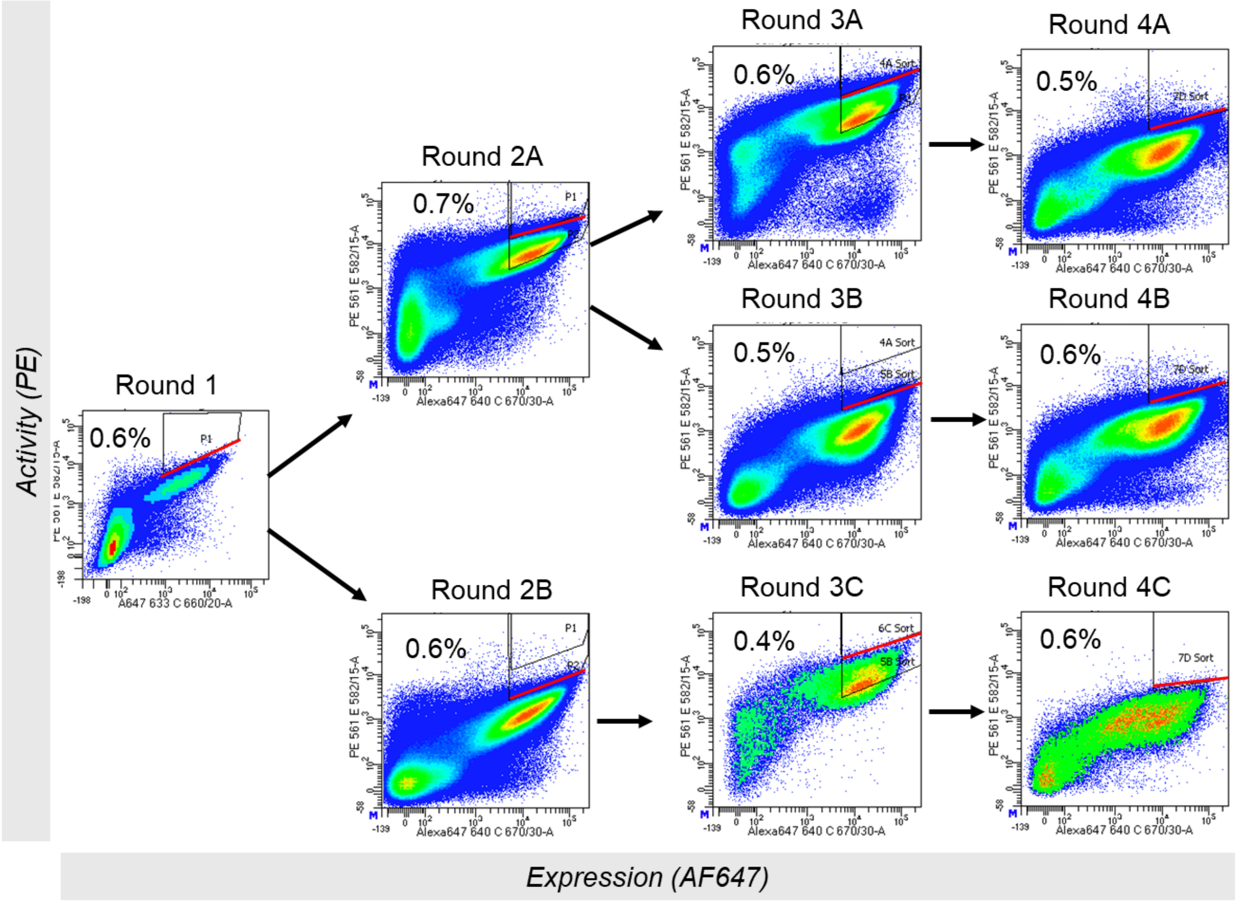

Figure S12. Representative DE campaign 1 FACS data and collection gates. The red line delineates the bottom boundary of the collection gate, and the percentage refers to the percent of total single cells kept. The *y*-axis represents sAv-PE fluorescence corresponding to the enzyme activity while the *x*-axis represents AlexaFluor 647 fluorescence which corresponds to the myc tag and therefore enzyme expression level. Instrument voltage settings varied between sorts 1, 2, 3, and 4, so absolute values on y- and x- axes cannot be compared between sorts.

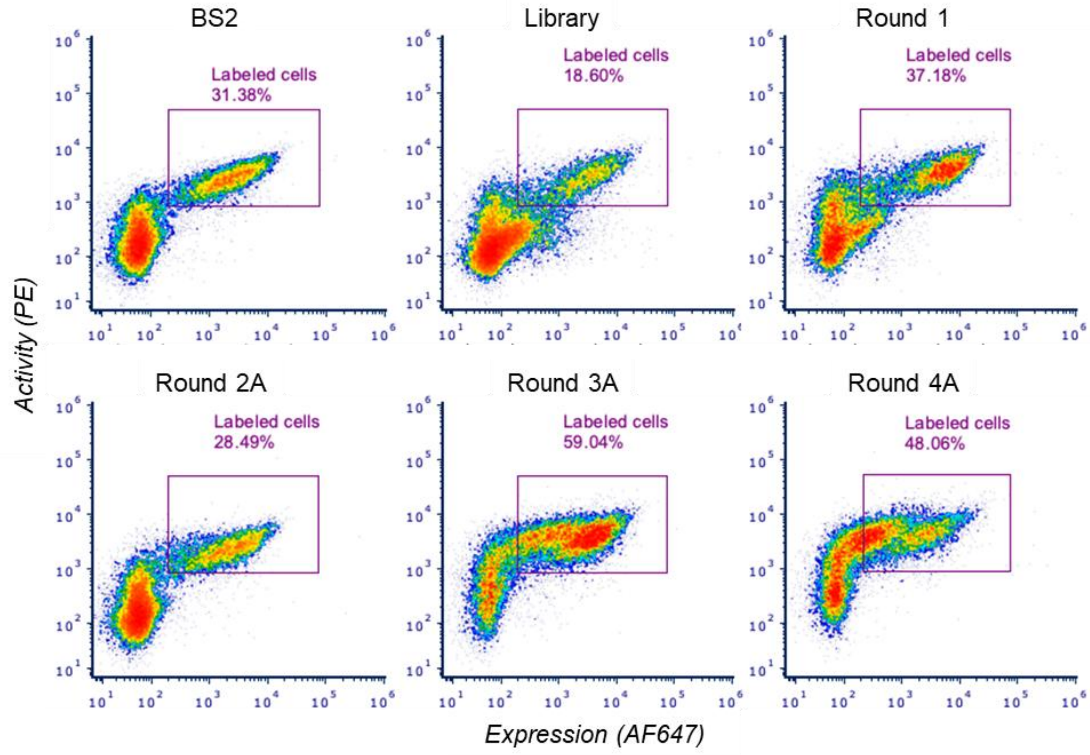

Figure S13. Flow cytometry data showing the parent enzyme, initial library, post round 1, 2, 3, and 4 sorted yeast. Data was collected using 1.25 μM AC-2 for 1 minute of labeling which was then reacted with biotin-PEG$_3$-Azide via CuAAC and stained with sAv-PE (*y*-axis) while the myc tag was stained with antibodies on the *x*-axis.

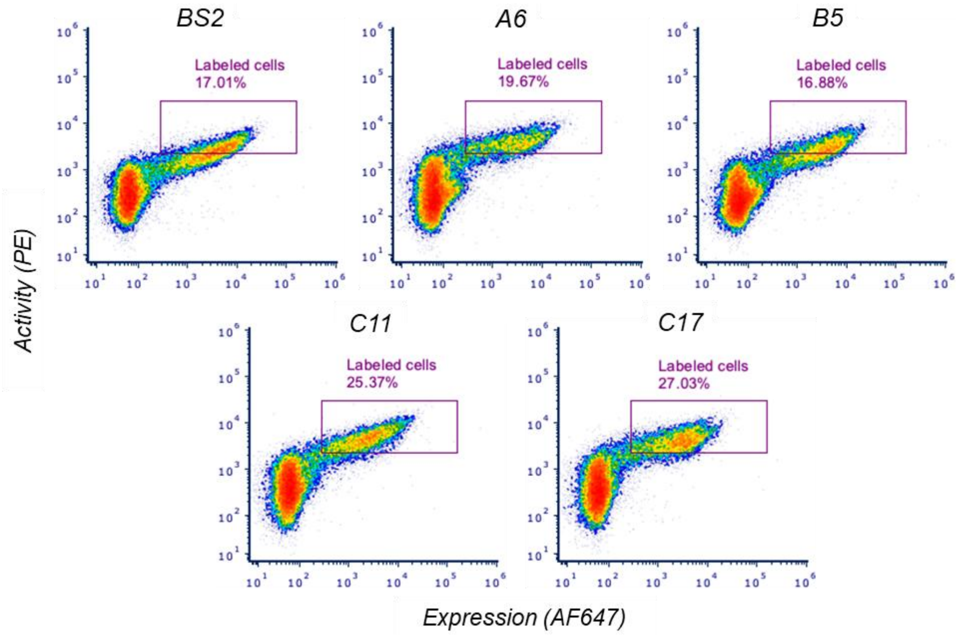

Figure S14. Activity of individual BS2 mutants on the cell surface towards AC-2 probe. The parent enzyme (BS2) and mutants A6 (BS2-LTE), B5, C11, C13, and C17 were evaluated for their activity against 1.25 µM AC-2 probe in a 5-second labeling period prior to enzyme quenching. AC-2 probe was reacted with biotin-PEG$_3$-Azide via CuAAC then stained with sAv-PE on the *y*-axis while the myc tag was stained with antibodies on the *x*-axis.

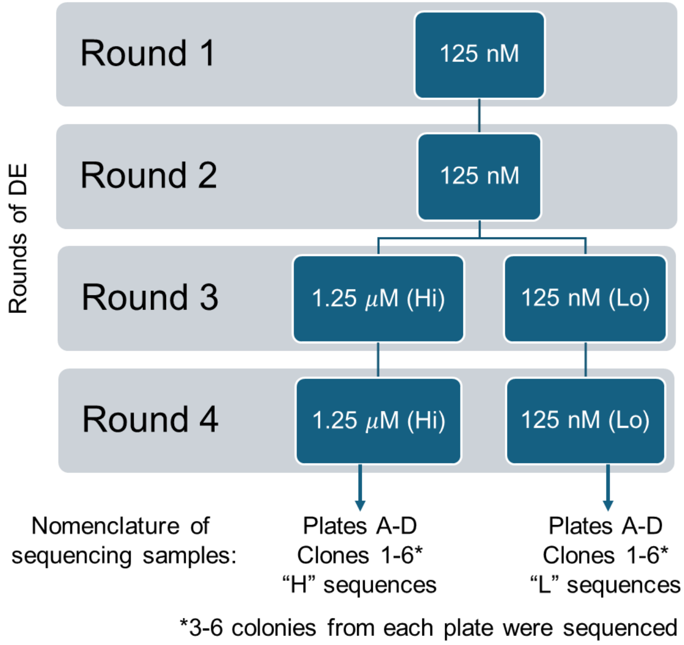

Figure S15. Flow chart demonstrating the parent populations and concentrations of SP-d5 probe used for the second DE campaign. 125 nM probe was used as an alternative to 1.25 µM probe to see if highly active variants in low probe concentrations could be identified. The nomenclature of the sequenced variants from DE 2 are also shown. "H/L" refer to the round 4 sort the sequence came from and "A/B/C/D" refer to the plate of bacterial transformants from which the clone was picked.

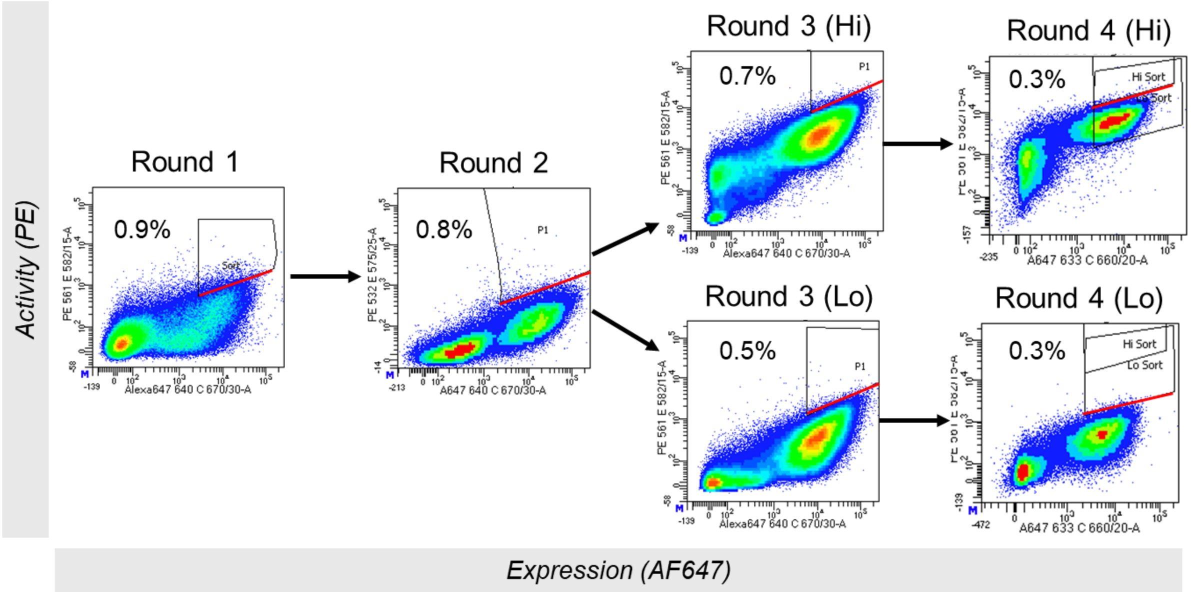

Figure S16. Representative DE campaign 2 FACS data and gate design. The red line delineates the bottom boundary of the collection gate, and the percentage refers to the percentage of total single cells kept. The *y*-axis represents sAv-PE fluorescence corresponding to the enzyme activity while the *x*-axis represents AlexaFluor 647 fluorescence which corresponds to the myc tag and therefore enzyme expression level. For each round of sorting 125 nM (rounds 1, 2, 3 Lo, and 4 Lo) or 1.25 μM (rounds 3 Hi and 4 Hi) SP-d5 probe was used to label the cells for 1 minute.

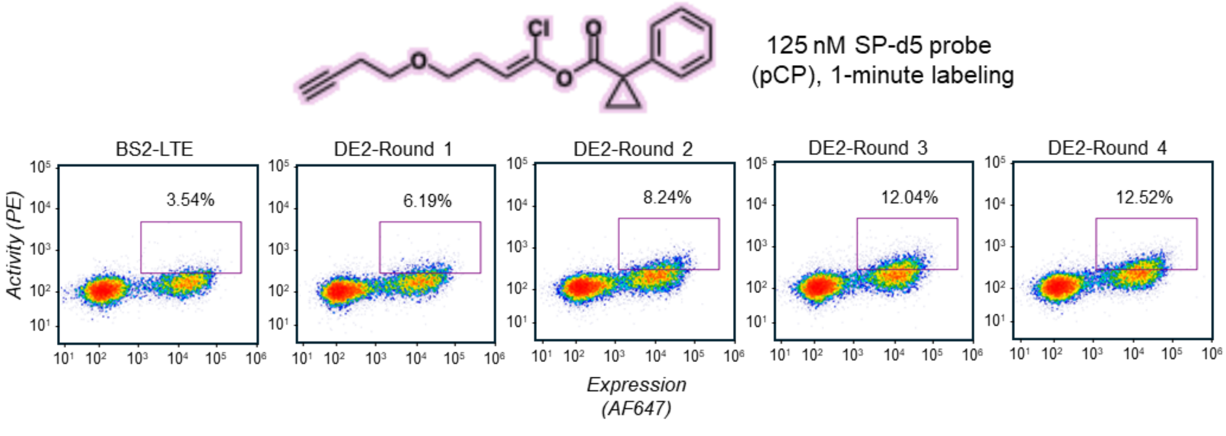

Figure S17. Flow cytometry analysis showing improvement in the second directed evolution (DE) campaign. Yeast samples consisted of monoclonal BS2-LTE or of an error-prone library of BS2-LTE mutants after 1–4 rounds of cell sorting. Cells were labeled using 125 nM SP-d5 probe. These are the same data as in Figure 2d of the main text, except this figure shows labeling of the intermediate rounds of sorted yeast samples.

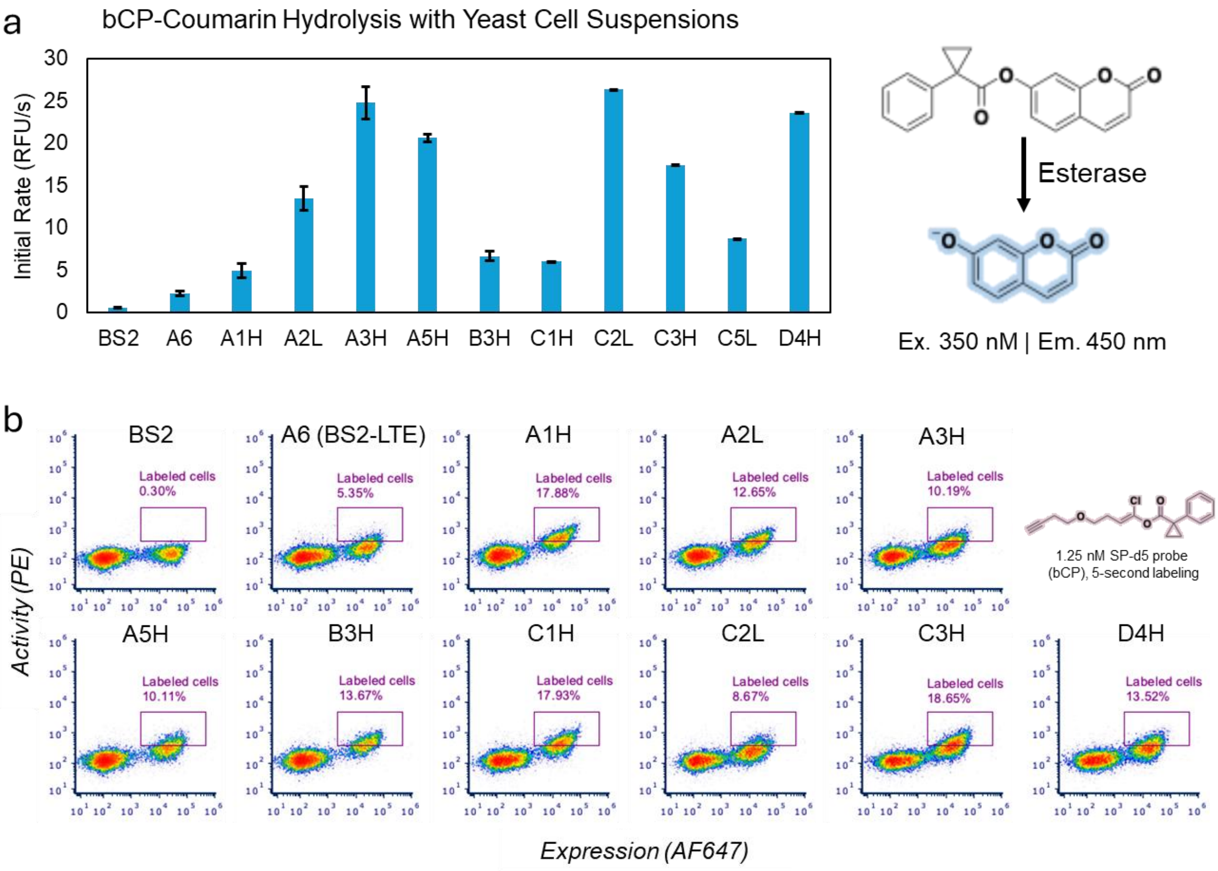

Figure S18. Activity of monoclonal yeast populations expressing BS2, BS2-LTE, and variants of BS2-LTE from the second DE campaign. See Table S3 for a list of mutations associated with each variant. A. Evaluation of BS2 and mutant kinetics using pCP-masked coumarin. Masked coumarin is hydrolyzed in the presence of esterase to reveal unmasked, fluorescent coumarin. Initial rate was measured by quantifying the rate of fluorescence increase over 95 seconds using 20 μM pCP-coumarin. Each experiment was performed in triplicate with the average shown in the bar graphs. Error bars represent ± standard deviation. B. Activity of individual BS2 mutants on the cell surface towards SP-d5 probe. The parent enzyme (BS2) and mutants were evaluated for their activity against 1.25 nM SP-d5 probe in a 5-second labeling period prior to enzyme quenching. AC-2 probe was reacted with biotin-PEG$_3$-Azide via CuAAC then stained with sAv-PE on the *y*-axis while the myc tag was stained with antibodies on the *x*-axis.

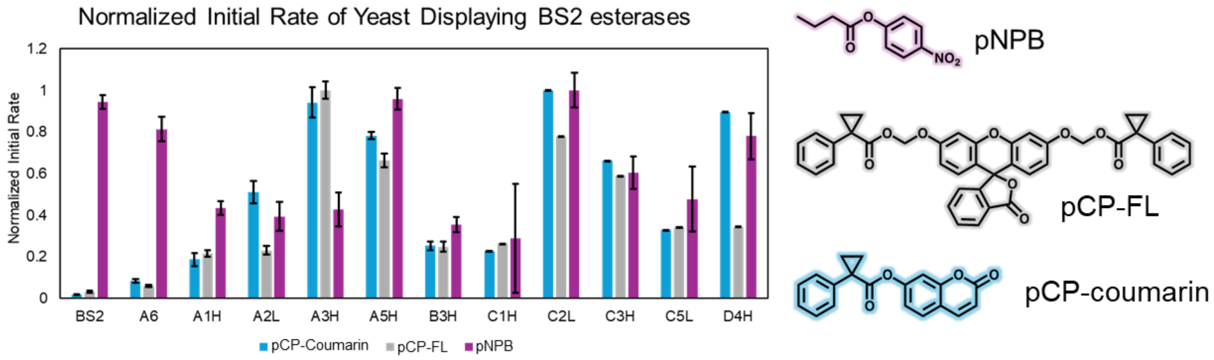

Figure S19. Activity of monoclonal yeast populations expressing BS2, BS2-LTE (A6 variant), and variants of BS2-LTE from the second DE campaign against various esters. See Table S3 for a list of mutations associated with each variant. Evaluation of BS2 and mutant kinetics using pCP-masked coumarin and fluorescein as well as pNPB (paranitrophenyl butyrate). Esters were hydrolyzed in the presence of esterase displayed on yeast cell surface and resulting chromogen or fluorophore was subsequently measured. Initial rate was measured by quantifying the rate of fluorescence increase over 85 seconds using 20 μM probe. Data was normalized such that the initial rate for the fastest mutant within each data set for a particular substrate was set to 1. For normalizing the following enzymes had the fastest rate: BS2-TRFLE ("C2L") for pNPB and pCP-coumarin, and BS2-ELLVAT ("A3H") for pCP-FL. Each experiment was performed in triplicate with the average shown in the bar graph. Error bars represent ± standard deviation.

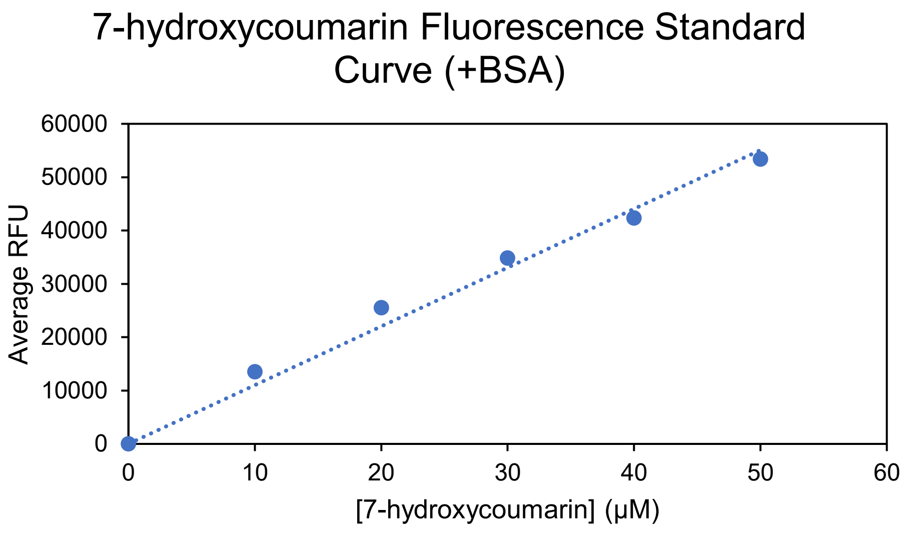

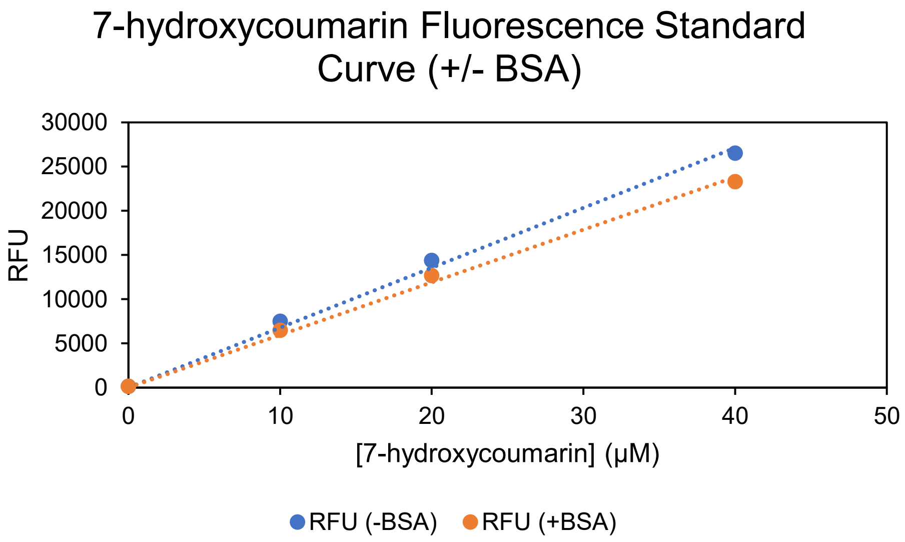

Figure S20. Representative standard curve used during coumarin substrate hydrolysis (described in Methods) demonstrating the relationship of 0–50 μM 7-hydroxycoumarin and the relative fluorescence values (RFU) in the presence of Bovine Serum Albumin. Values were recorded in triplicate. Black Error bars demonstrate the standard deviation of the average concentration of 7-hydroxycoumarin from 3 independent measurements. Black error bars are present for all data points, but their visibility may be occluded by the dots. (Bottom): Comparison standard curve of 7-hydroxycoumarin in the presence and absence of Bovine Serum Albumin.

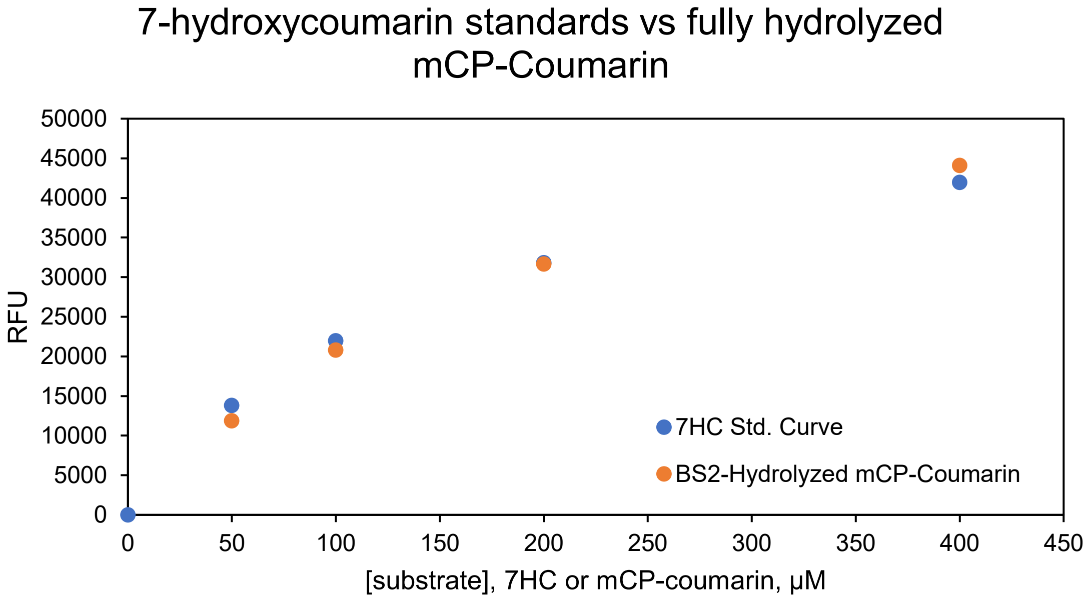

Figure S21. Plots demonstrating the relative fluorescence units (RFU) values obtained when comparing commercial 7-hydroxycoumarin standards to BS2-hydrolyzed mCP-coumarin samples (as described in Methods).

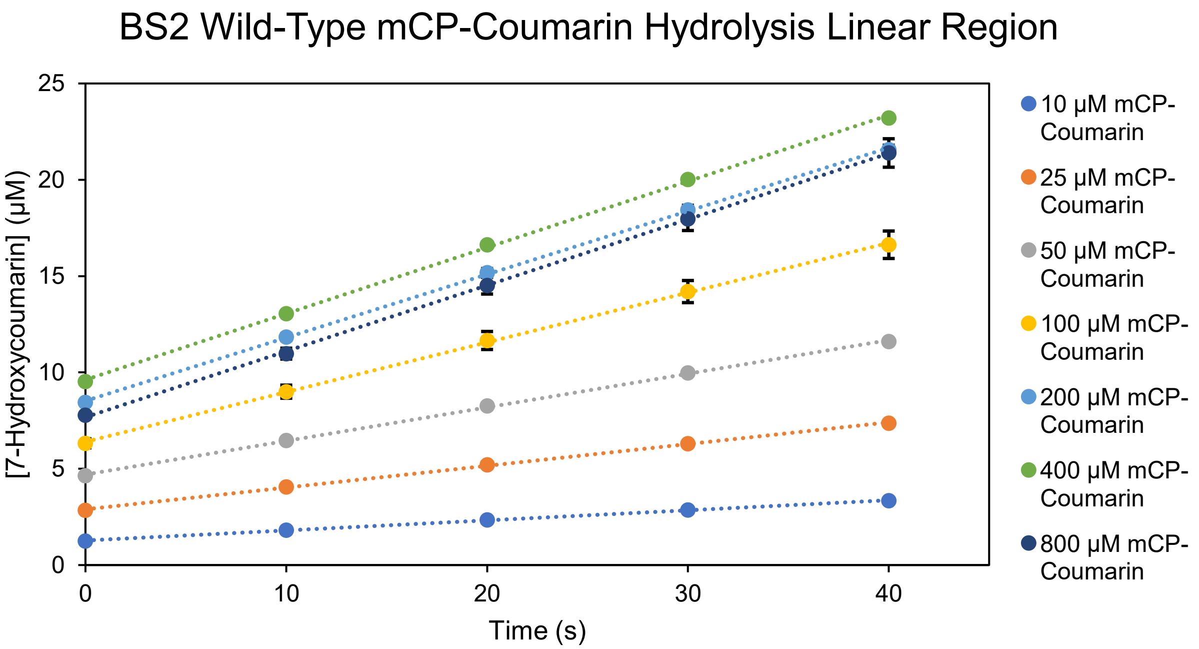

Figure S22. Linear region of mCP-coumarin hydrolysis catalyzed by wild-type BS2 esterase with varying concentrations of mCP-coumarin. Each colored dot represents the average product (7-hydroxycoumarin) concentration obtained from 3 independent *in vitro* reactions at a given concentration of mCP-coumarin. Black error bars demonstrate the standard deviation of the average concentration of 7-hydroxycoumarin from 3 independent *in vitro* reactions. Black error bars are present for all data points, but their visibility may be occluded by the dots. Dashed lines show the calculated best-fit lines for each condition. All the samples exhibited nonzero fluorescence at time = 0 owing to the delay between the start of the reaction and the first measurement made by the platereader.

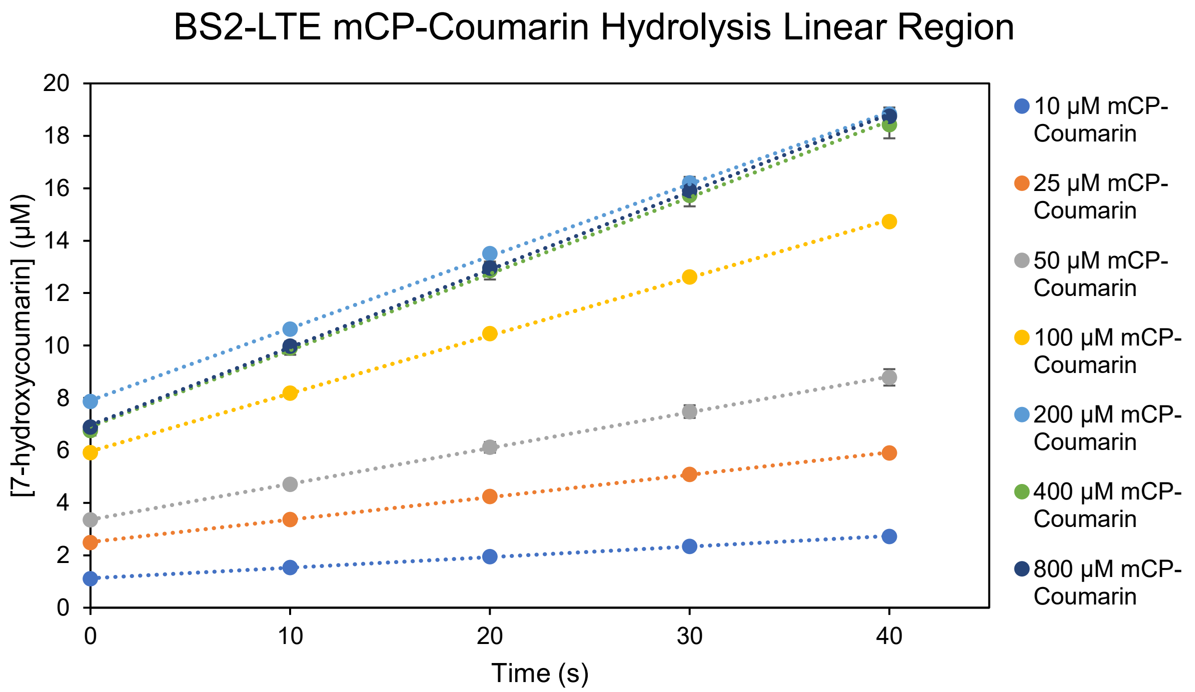

Figure S23. Linear region of mCP-coumarin hydrolysis catalyzed by BS2-LTE with varying concentrations of mCP-coumarin. Each colored dot represents the average product (7-hydroxycoumarin) concentration obtained from 3 independent *in vitro* reactions at a given concentration of mCP-coumarin. Black error bars demonstrate the standard deviation of the average concentration of 7-hydroxycoumarin from 3 independent *in vitro* reactions. Black error bars are present for all data points, but their visibility may be occluded by the dots. Dashed lines show the calculated best-fit lines for each condition. All the samples exhibited nonzero fluorescence at time = 0 owing to the delay between the start of the reaction and the first measurement made by the platereader.

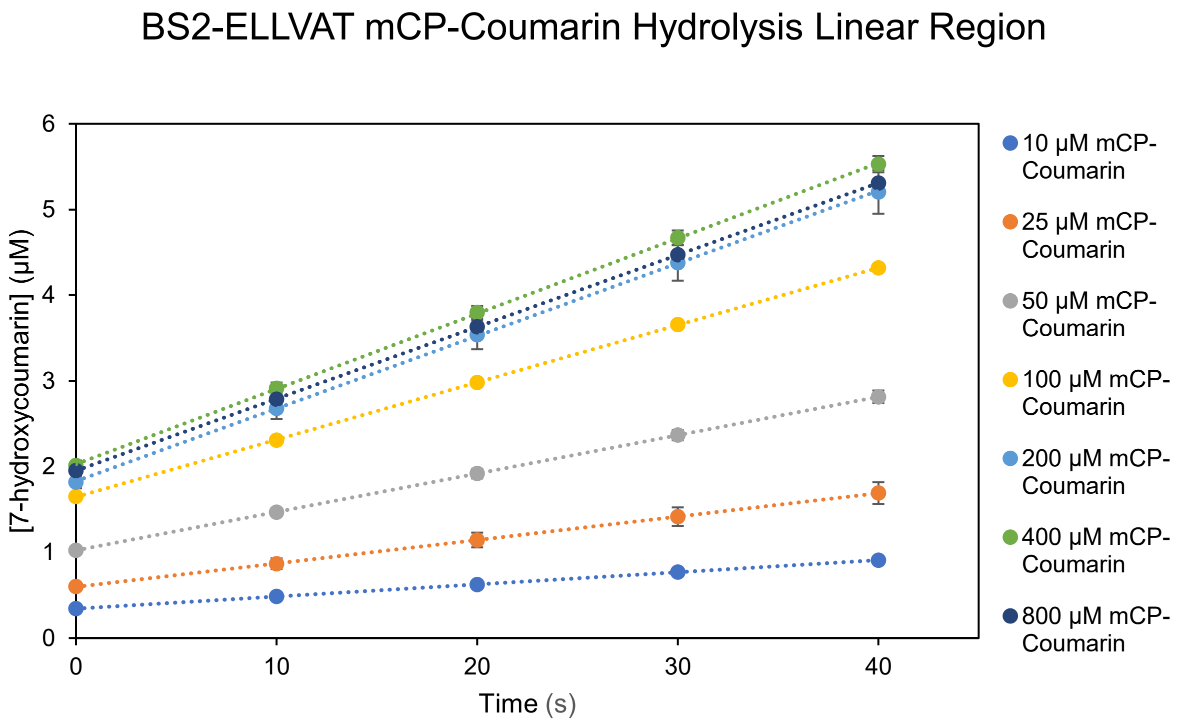

Figure S24. Linear region of mCP-coumarin hydrolysis catalyzed by BS2-ELLVAT with varying concentrations of mCP-coumarin. Each colored dot represents the average product (7-hydroxycoumarin) concentration obtained from 3 independent *in vitro* reactions at a given concentration of mCP-coumarin. Black error bars demonstrate the standard deviation of the average concentration of 7-hydroxycoumarin from 3 independent *in vitro* reactions. Black error bars are present for all data points, but their visibility may be occluded the dots. Dashed lines show the calculated best-fit lines for each condition. All the samples exhibited nonzero fluorescence at time = 0 owing to the delay between the start of the reaction and the first measurement made by the platereader.

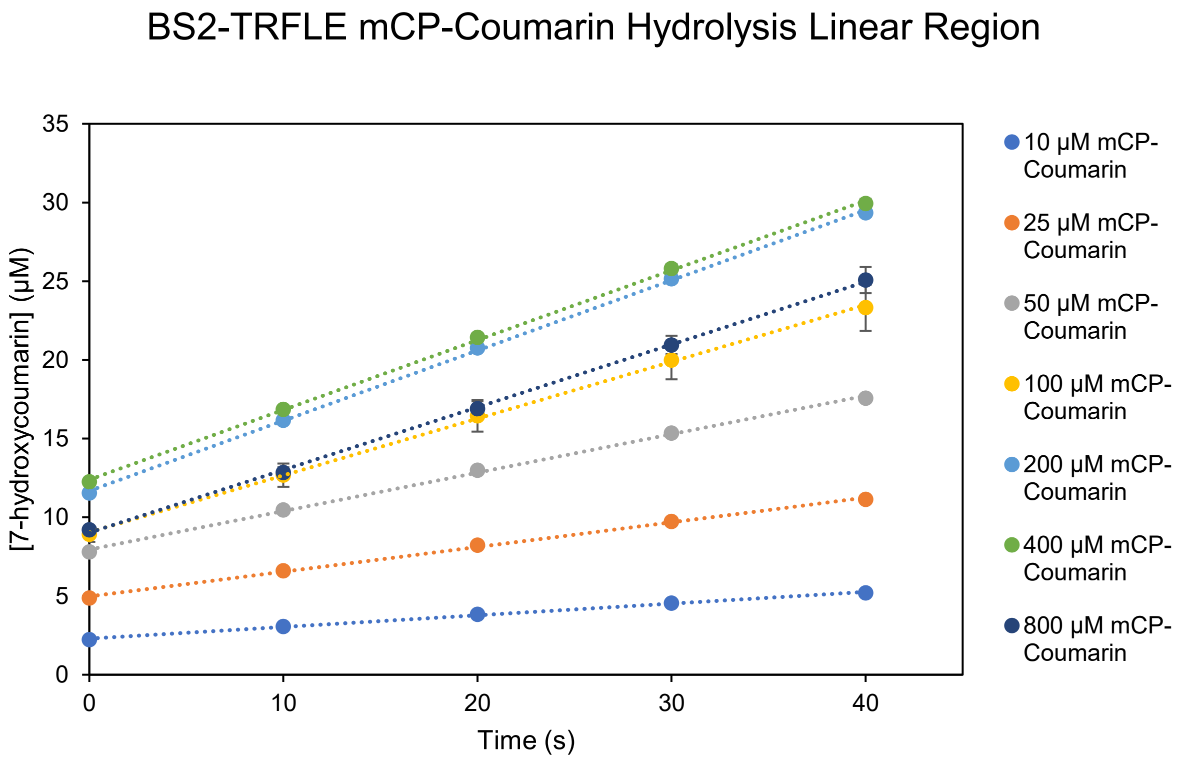

Figure S25. Linear region of mCP-coumarin hydrolysis catalyzed by BS2-TRFLE with varying concentrations of mCP-coumarin. Each colored dot represents the average product (7-hydroxycoumarin) concentration obtained from 3 independent *in vitro* reactions at a given concentration of mCP-coumarin. Black error bars demonstrate the standard deviation of the average concentration of 7-hydroxycoumarin from 3 independent *in vitro* reactions. Black error bars are present for all data points, but their visibility may be occluded by the dots. Dashed lines show the calculated best-fit lines for each condition. All the samples exhibited nonzero fluorescence at time = 0 owing to the delay between the start of the reaction and the first measurement made by the platereader.

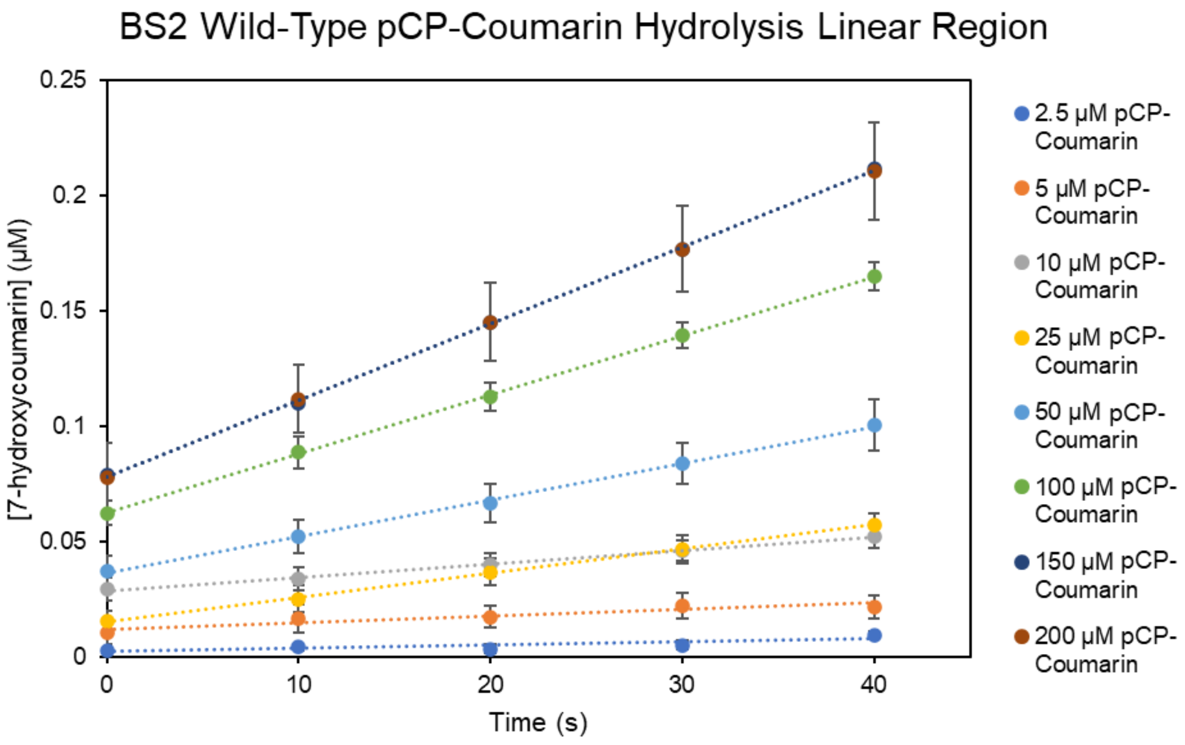

Figure S26. Linear region of pCP-coumarin hydrolysis catalyzed by wild-type BS2 esterase with varying concentrations of pCP-coumarin. Each colored dot represents the average product (7-hydroxycoumarin) concentration obtained from 3 independent *in vitro* reactions at a given concentration of pCP-coumarin. Black error bars demonstrate the standard deviation of the average concentration of 7-hydroxycoumarin from 3 independent *in vitro* reactions. Black error bars are present for all data points, but their visibility may be occluded by the dots. Dashed lines show the calculated best-fit lines for each condition. All the samples exhibited nonzero fluorescence at time = 0 owing to the delay between the start of the reaction and the first measurement made by the platereader.

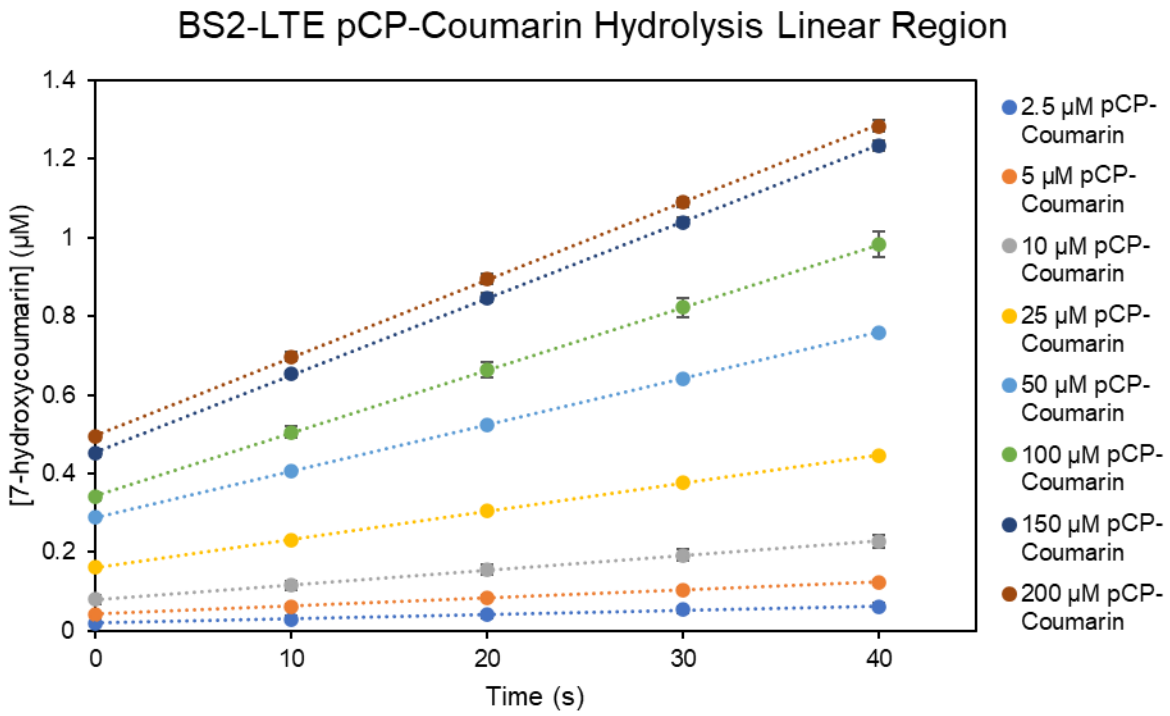

Figure S27. Linear region of pCP-coumarin hydrolysis catalyzed by BS2-LTE with varying concentrations of pCP-coumarin. Each colored dot represents the average product (7-hydroxycoumarin) concentration obtained from 3 independent *in vitro* reactions at a given concentration of pCP-coumarin. Black Error bars demonstrate the standard deviation of the average concentration of 7-hydroxycoumarin from 3 independent *in vitro* reactions. Black error bars are present for all data points, but their visibility may be occluded by the dots. Dashed lines show the calculated best-fit lines for each condition. All the samples exhibited nonzero fluorescence at time = 0 owing to the delay between the start of the reaction and the first measurement made by the platereader.

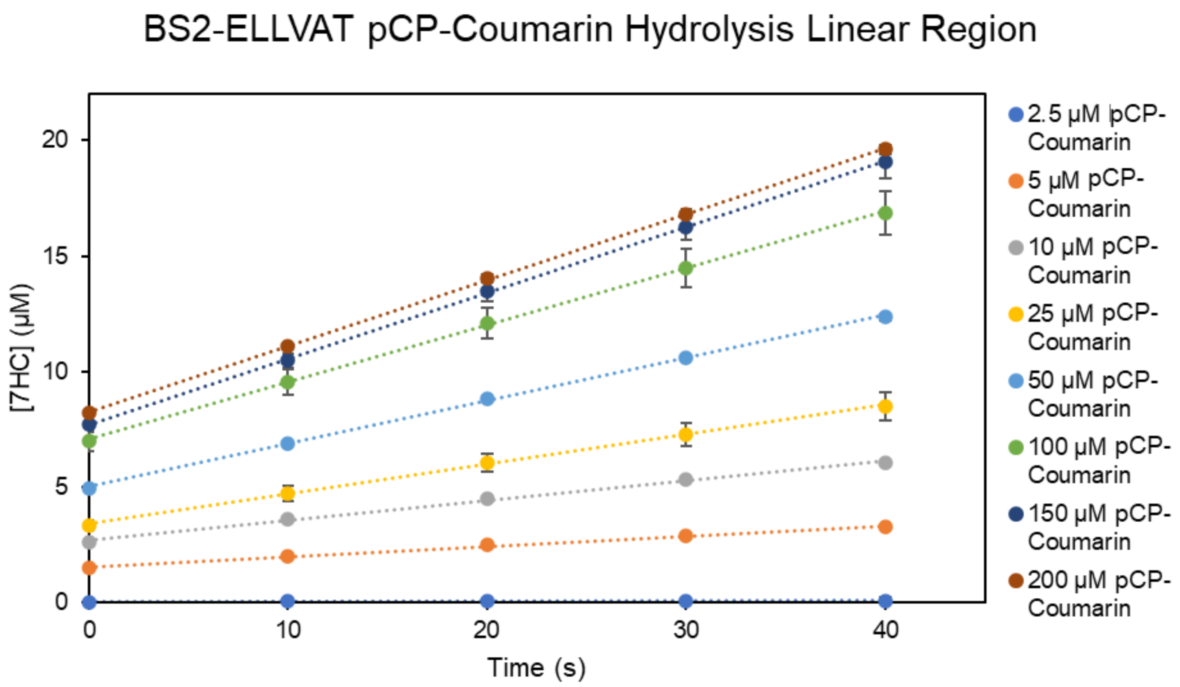

Figure S28. Linear region of pCP-coumarin hydrolysis catalyzed by BS2-ELLVAT with varying concentrations of pCP-coumarin. Each colored dot represents the average product (7-hydroxycoumarin) concentration obtained from 3 independent *in vitro* reactions at a given concentration of pCP-coumarin. Black error bars demonstrate the standard deviation of the average concentration of 7-hydroxycoumarin from 3 independent *in vitro* reactions. Black error bars are present for all data points, but their visibility may be occluded by the dots. Dashed lines show the calculated best-fit lines for each condition. All the samples exhibited nonzero fluorescence at time = 0 owing to the delay between the start of the reaction and the first measurement made by the platereader.

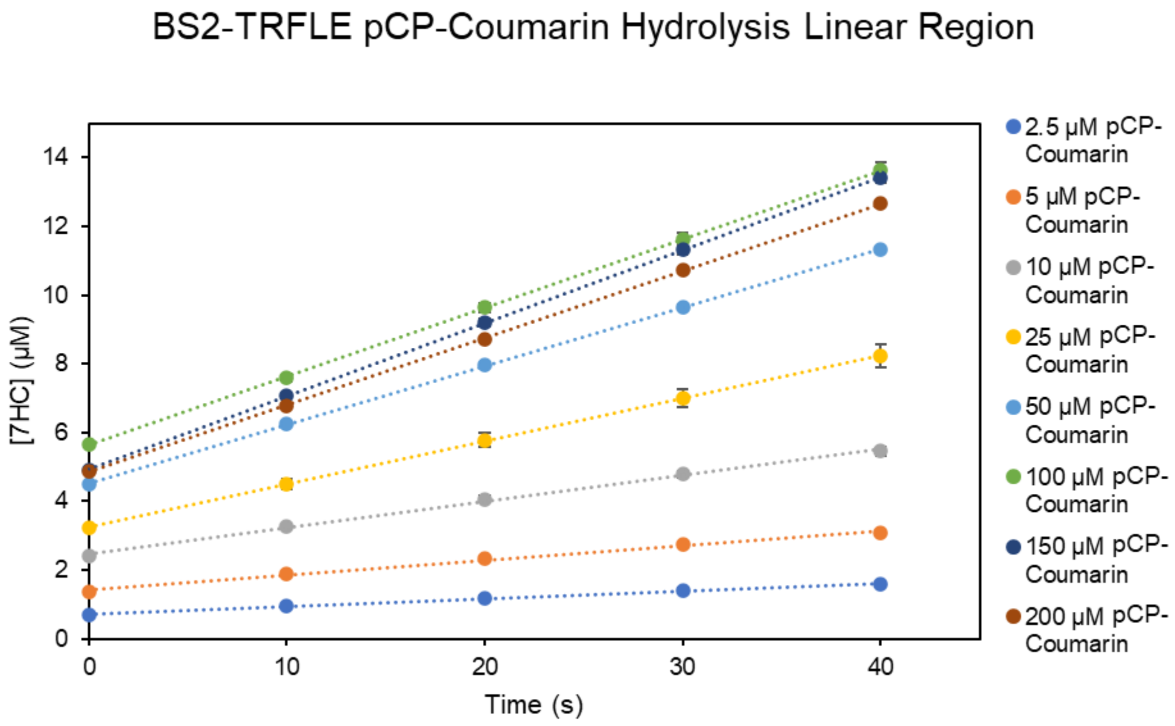

Figure S29. Linear region of pCP-coumarin hydrolysis catalyzed by BS2-TRFLE with varying concentrations of pCP-coumarin. Each colored dot represents the average product (7-hydroxycoumarin) concentration obtained from 3 independent *in vitro* reactions at a given concentration of pCP-coumarin. Black Error bars demonstrate the standard deviation of the average concentration of 7-hydroxycoumarin from 3 independent *in vitro* reactions. Black error bars are present for all data points, but their visibility may be occluded by the dots. Dashed lines show the calculated best-fit lines for each condition. All the samples exhibited nonzero fluorescence at time = 0 owing to the delay between the start of the reaction and the first measurement made by the platereader.

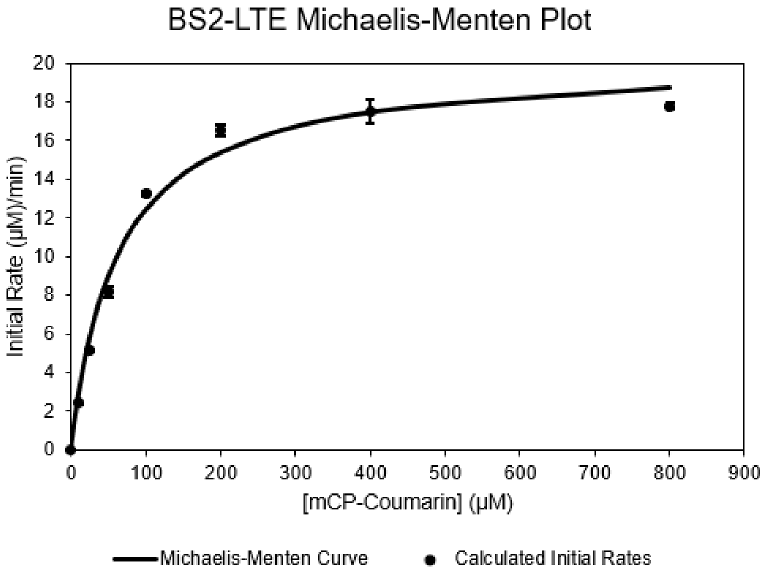

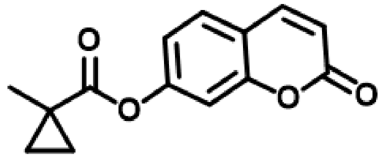

k_{cat}	33.7 s^{-1} (32.1-35.3)
K_m	63 μM (53.4-74.2)

Figure S30. Michaelis-Menten plot of BS2-LTE exhibiting the initial rate (μM/min) of mCP-coumarin hydrolysis as a function of mCP-coumarin concentration (μM). Black dots represent the average calculated initial rates of 3 independent *in vitro* reactions. The black curve is the calculated best-fit Michaelis-Menten curve to the data. Black error bars represent the standard deviation from the average calculated initial rates from 3 independent *in vitro* reactions per substrate concentration. Black error bars are present for all data points, but their visibility is occluded in some cases by the dots. Michaelis-Menten parameters are presented in the table on the right (numbers in parentheses indicate 95% confidence intervals).

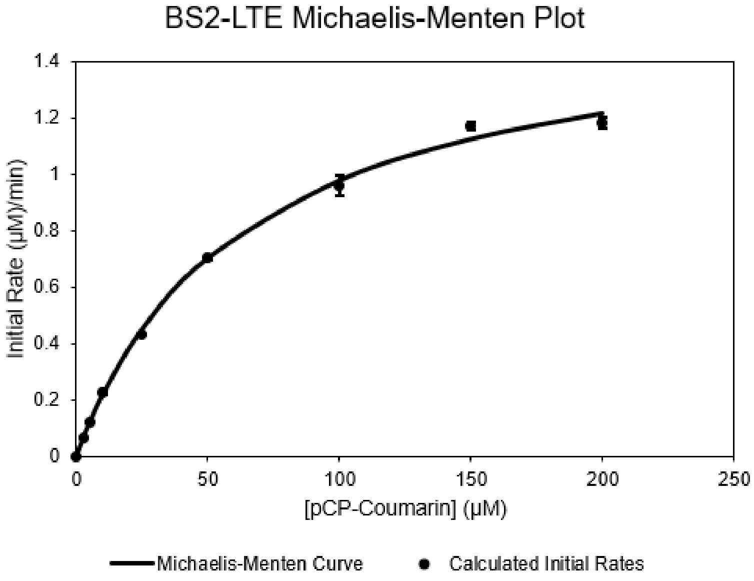

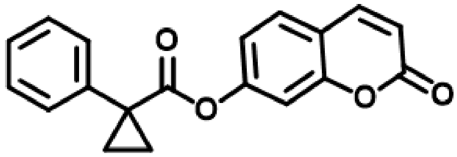

k_{cat}	2.7 s^{-1} (2.6-2.8)
K_m	65 μM (57.6-72.5)

Figure S31. Michaelis-Menten plot of BS2-LTE exhibiting the initial rate (μM/min) of pCP-coumarin hydrolysis as a function of pCP-coumarin concentration (μM). Black dots represent the average calculated initial rates of 3 independent *in vitro* reactions. The black curve is the calculated best-fit Michaelis-Menten curve to the data. Black error bars represent the standard deviation from the average calculated initial rates from 3 independent *in vitro* reactions per substrate concentration. Black error bars are present for all data points, but their visibility is occluded in some cases by the dots. Michaelis-Menten parameters are presented in the table on the right (numbers in parentheses indicate 95% confidence intervals).

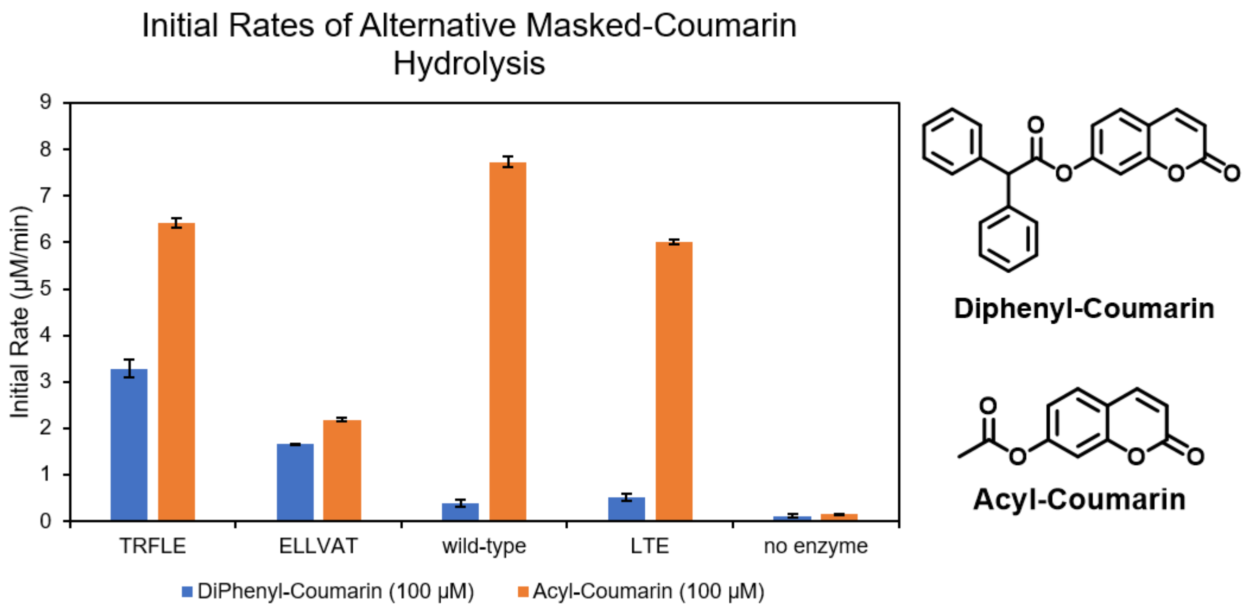

Figure S32. Initial rates of hydrolysis catalyzed by select purified BS2 variants (wild-type, LTE, ELLVAT, and TRFLE) against alternative masked-coumarin substrates (Diphenyl-Coumarin; Acyl-Coumarin). Initial rates were measured by quantifying the rate of fluorescence increase over 50 seconds using 100 µM of each substrate. Colored bars represent the average rate of 7-hydroxycoumarin formation obtained from 3 independent *in vitro* reactions. Black error bars represent the ± standard deviation of the initial rates from 3 independent *in vitro* reactions.

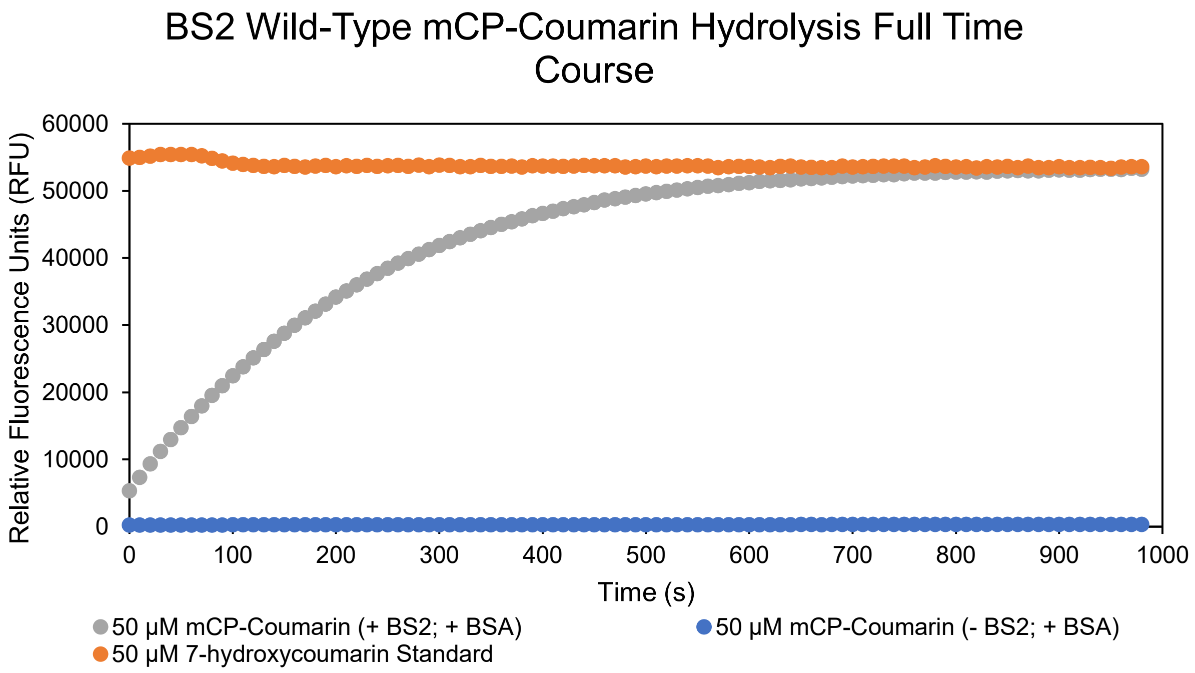

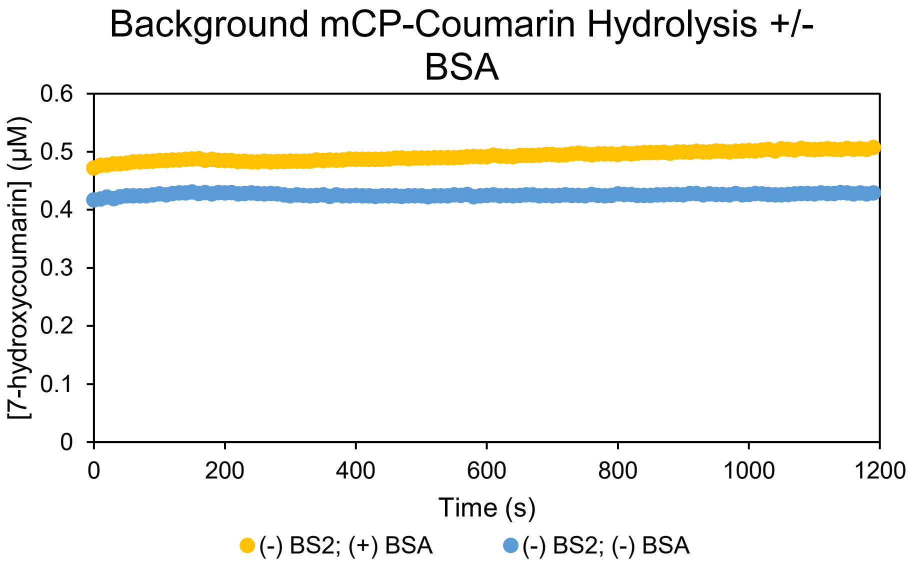

Figure S33. (Top): Plots demonstrating that BS2-catalyzed mCP-coumarin hydrolysis reaches completion (gray; orange), and that background hydrolysis in the absence of enzyme is negligible (blue). (Bottom): Comparison of non-BS2-catalyzed mCP-coumarin (50 µM) hydrolysis in the presence/absence of BSA, demonstrating that BSA itself contributes negligibly to the hydrolysis of mCP-coumarin.

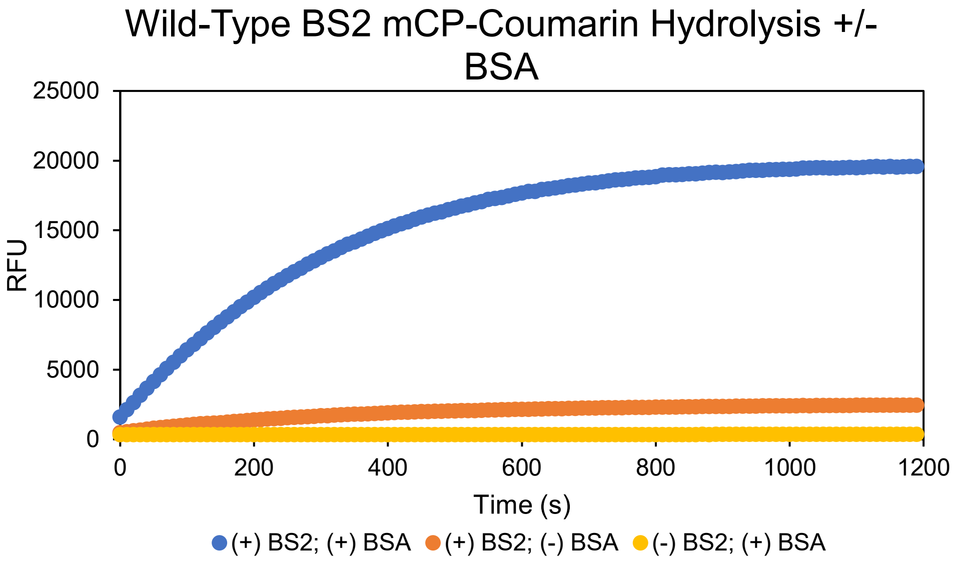

Figure S34. BS2-catalyzed mCP-coumarin (30 μM) hydrolysis in the presence/absence of 0.1 mg/mL BSA.

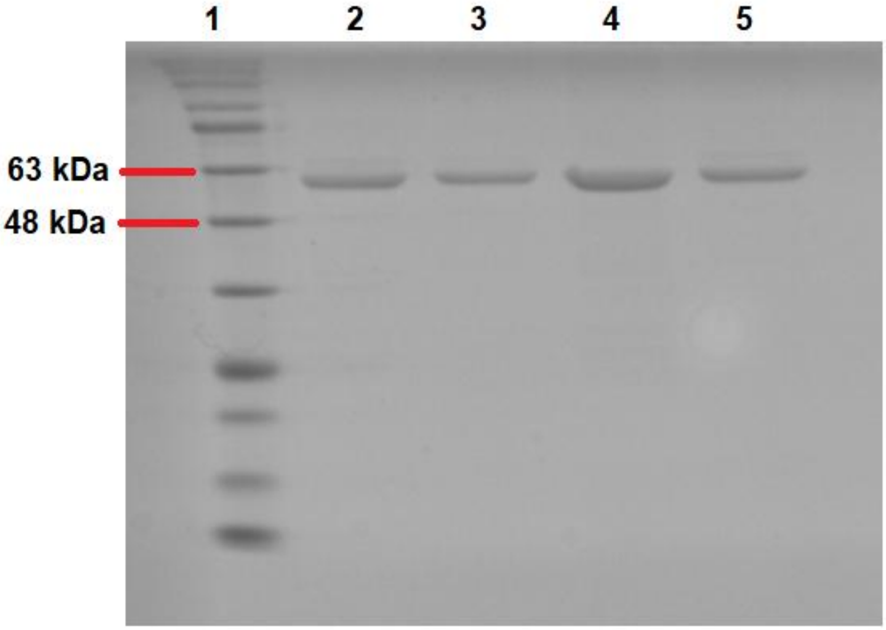

Figure S35. Representative 12% SDS-PAGE gel of BS2 esterase variants overexpressed and purified from *E coli.*. Proteins were purified according to the Methods section and were visualized by staining with Coomassie Brilliant Blue. Lanes: (1) BLUEstain™ Protein ladder (GoldBio), 11–245 kDa, (2) Wild-Type BS2, (3) BS2-LTE, (4) BS2-ELLVAT, (5) BS2-TRFLE. See Table S3 for a list of mutations associated with each variant.

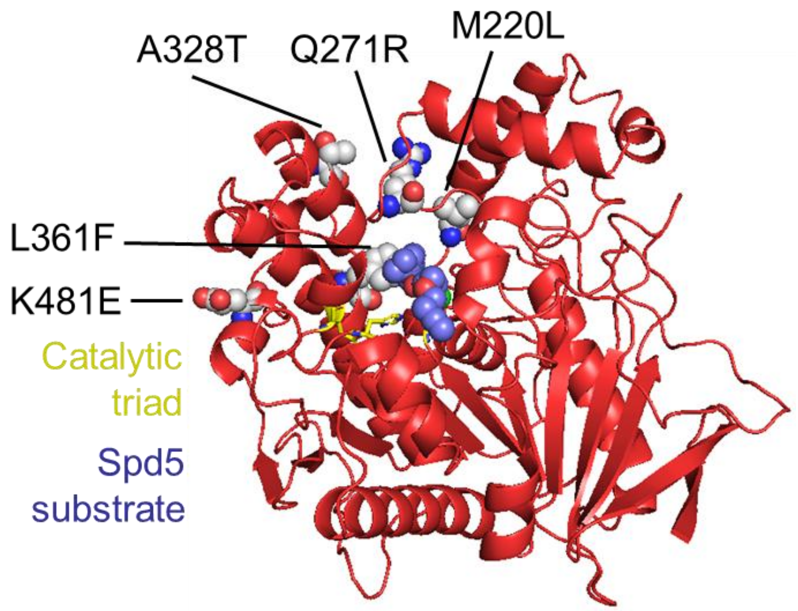

Figure S36. AlphaFold3 structural prediction for BS2-TRFLE variant with docking of the Spd5 substrate in the active site. The mutations in BS2-TRFLE relative to wt BS2 are depicted, along with the catalytic triad residues. This figure is the same as Figure 4e in the main text, except that the perspective is zoomed out. This depiction illustrates that the 5 beneficial mutations in BS2-TRFLE are clustered in the same region of the protein structure; none of the beneficial mutations are in the large β sheet or the surrounding helices in the lower part of the structure.

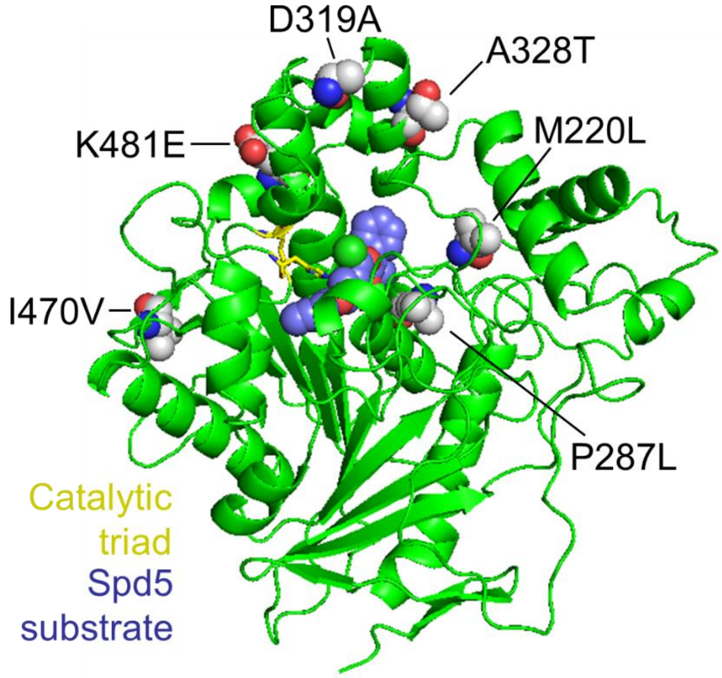

Figure S37. AlphaFold3 structural prediction BS2-ELLVAT with docking of the Spd5 substrate in the active site. The mutations in BS2-ELLVAT relative to wt BS2 are depicted, along with the catalytic triad residues.

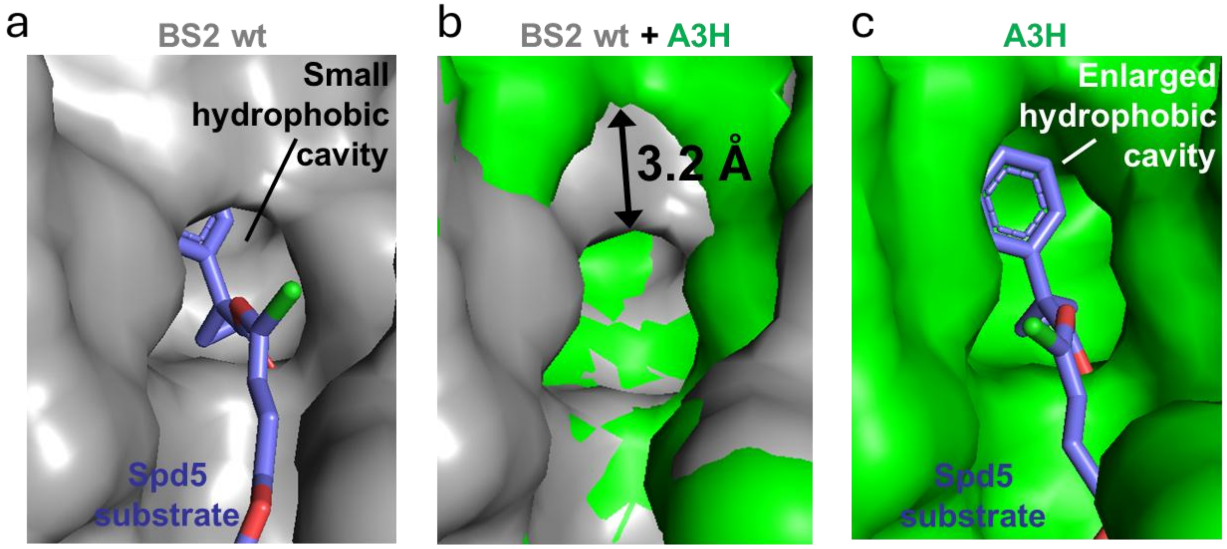

Figure S38. Structural modeling with Spd5 probe docking for BS2-ELLVAT. (a) AlphaFold3 structural prediction for wt BS2 with docking of the Spd5 substrate in the active site. (b) Overlay of AlphaFold3 structure predictions for wt BS2 and the BS2-TRFLE variant, illustrating that the opening to the hydrophobic cavity in the back of the active site has been enlarged by ~3.2 Å in the A3H variant. This appears to be a greater enlargement compared to BS2-TRFLE, although the enlargement is determined based on the predicted conformation of Ile269. The positioning of the backbone atoms for Ile239 is similar in the predicted BS2-ELLVAT and BS2-TRFLE structures. (c) AlphaFold3 structural prediction for BS2-ELLVAT with docking of the Spd5 substrate in the active site.

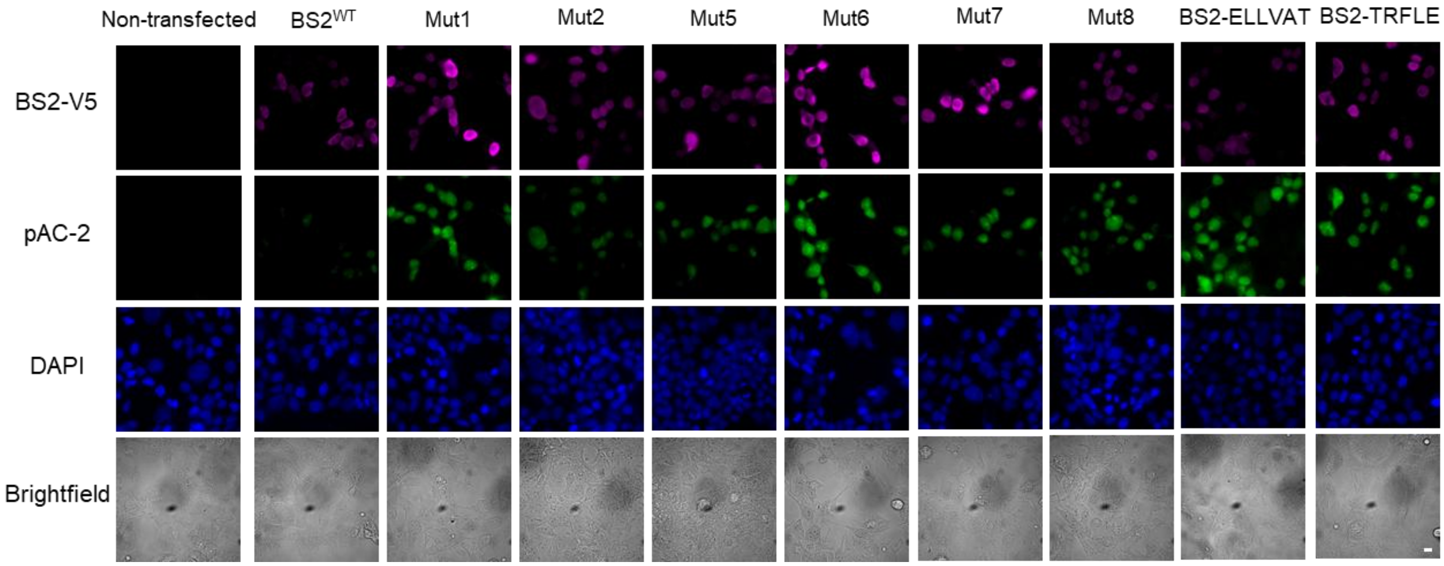

Figure S39. pAC-2 labeling activity in the nucleus of HEK293T cells for the top BS2 variants. (see Table S3 for a list of mutations associated with each variant). a) 24 hr post transfection with BS2 mutant plasmids containing NLS tag, 100 µM of pAC-2 probe was added to the cells for 10 min at 37 °C. Labeling activity was measured by immunofluorescence imaging post click reaction with fluorophore AF488. DAPI is a nuclear marker. Scale bar on the bottom right image represents 10 µm.

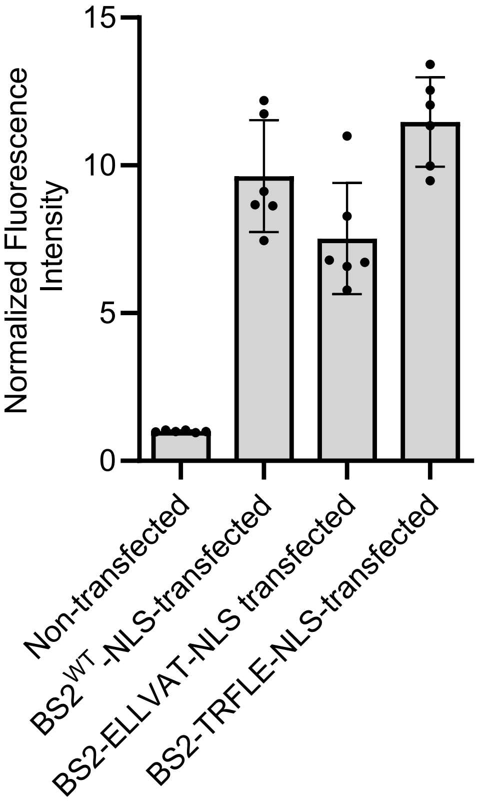

Figure S40. Comparison of expression profile of top BS2 mutants in the nucleus of HEK293T cells. 24 hr post transient transfection, expression of wtBS2 and mutants were assessed by immunofluorescence using V5-antibody staining. Mean fluorescence intensity was measured for all nuclei using DAPI threshold. Normalized fluorescence intensity values of all replicates are plotted, with error bars showing the standard deviation (n=6 different fields of view).

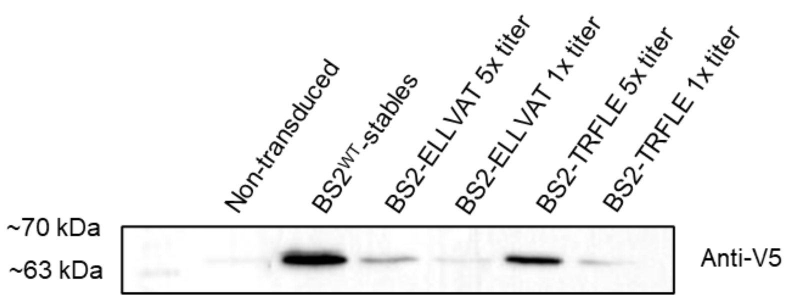

Figure S41. Western blot showing expression of wtBS2 and mutants A3H and C2L in the nucleus of MDA-MB-231 cells via lentiviral transduction (variants A3H and C2L are called BS2-ELLVAT and BS2-TRFLE, respectively, in the main text). wtBS2 stables refers to MDA-MB-231 cells stably expressing wtBS2 in the nucleus. For the mutants, lentivirus of different titers (1x >= 5 MOI) of either A3H or C2L construct was added to MDA-MB-231 cells, and the expression was measured by Western blot 3 days after lentiviral transduction using Anti-V5 antibody.

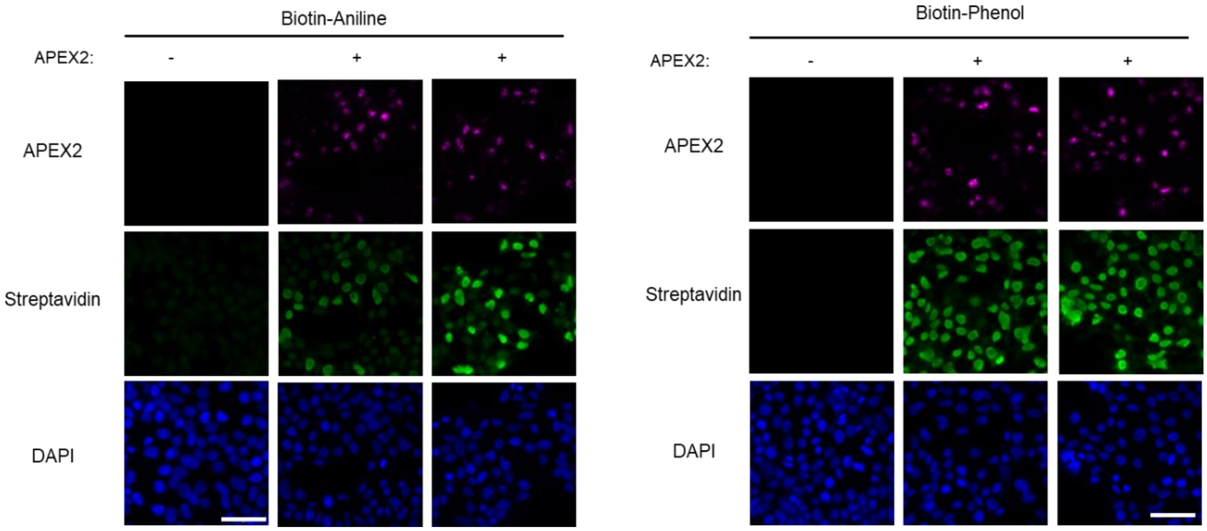

Figure S42. Comparison of nucleolar labeling in HEK293T cells with APEX2 and (a) Biotin-Aniline or (b) Biotin-Phenol. 24 hr post-transfection of APEX2-GFP-Nik3x plasmids, cells were treated with 0.5 mM Biotin-Aniline or 0.5 mM Biotin-Phenol for 30 min, followed by 1 min treatment of 1 mM H_2O_2. Labeling was observed with widefield immunofluorescence imaging of streptavidin-647. DAPI is a nuclear marker. Scale bar on DAPI channel represents 50 µm. Two representative fields of view are shown for the "+ probe" conditions.

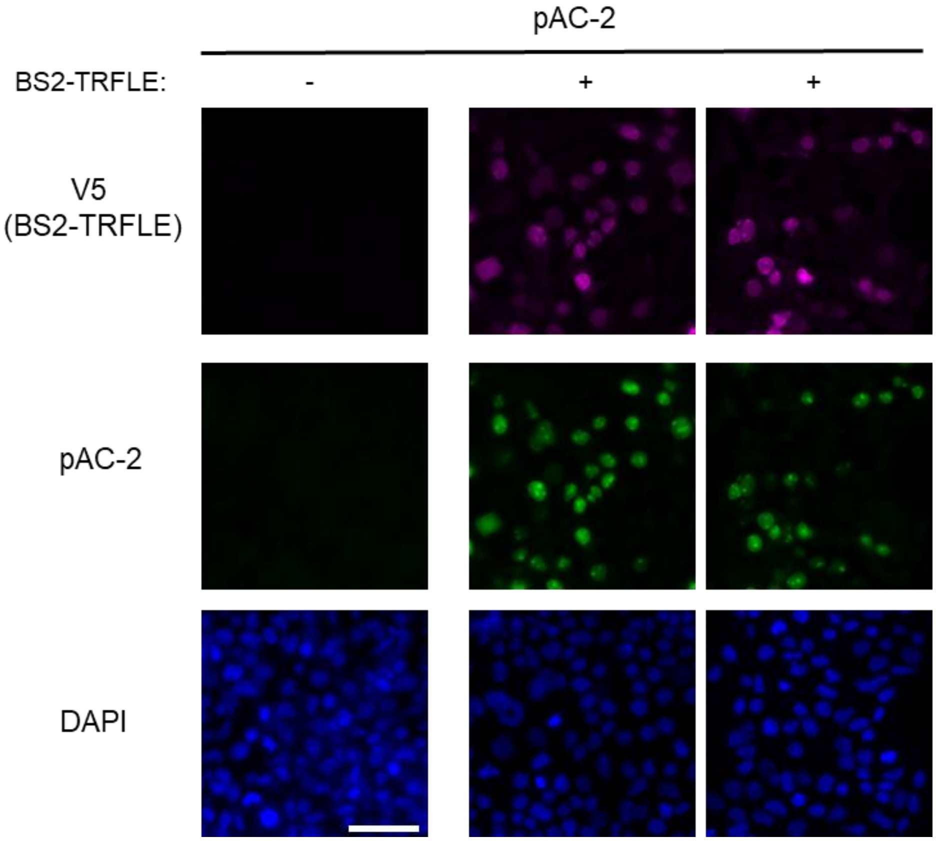

Figure S43. Comparison of nucleolar labelling with BS2-TRFLE and 50 µM pAC-2 in HEK293T cells. 24 hr post-transfection of V5-BS2-TRFLE-Nik3x plasmids, cells were treated with 50 µM pAC-2 for 5 min. Labeling was observed with widefield immunofluorescence imaging post click reaction with fluorophore AF488. BS2-TRFLE expression was visualized with anti-V5 primary and Anti-mouse-555 secondary antibody staining. DAPI is a nuclear marker. Scale bar on DAPI channel represents 50 µm. Two representative fields of view are shown for the "+ probe" condition.

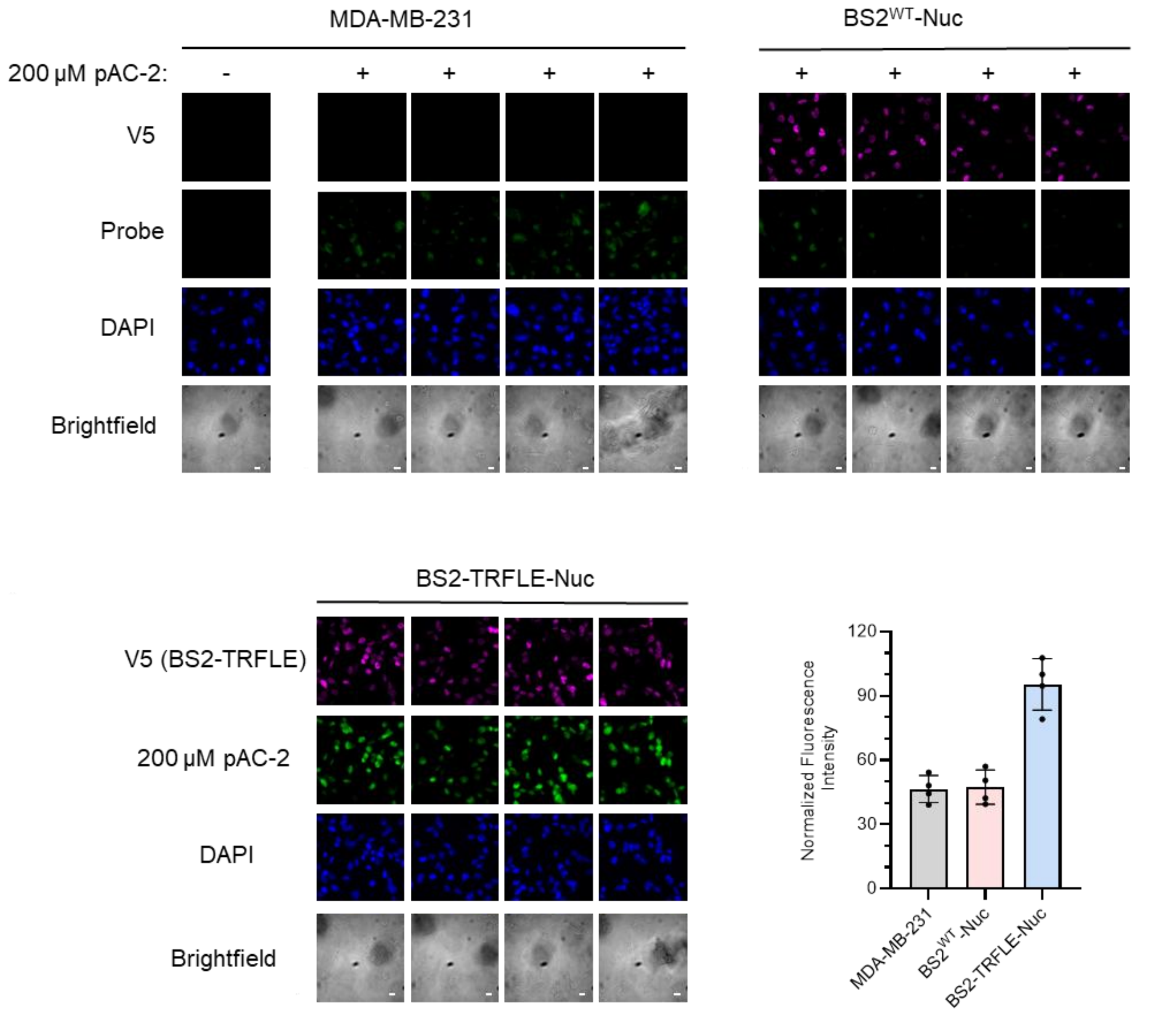

Figure S44. Assessing pAC-2 labeling activity with $BS2^{WT}$ and BS2-TRFLE. MDA-MB-231 cells stably expressing nuclear $BS2^{WT}$ or BS2-TRFLE were treated with 200 µM pAC-2 probe for 5 min. Labeling activity was measured by immunofluorescence imaging post click reaction with fluorophore AF488. DAPI is a nuclear marker. The scale bars on the bottom of the brightfield images represent 10 µm. Normalized fluorescence intensity values of all replicates are plotted, with error bars showing the standard deviation (n=4 different fields of view).

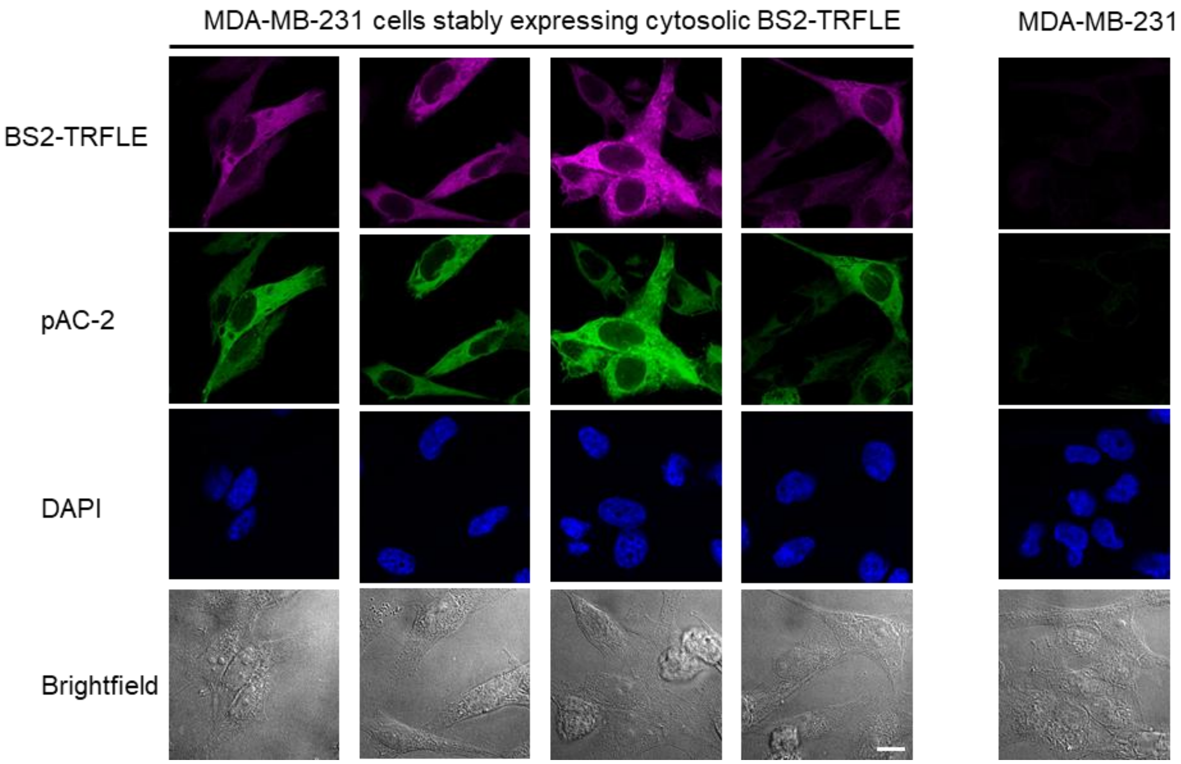

Figure S45. MDA-MB-231 cells stably expressing BS2-TRFLE in the cytosol were treated with 100 µM pAC-2 for 5 min. Cells were fixed, permeabilized, clicked with AF-488 for visualization. BS2-TRFLE was visualized through V5 antibody staining. Scale bar represents 10 µm.

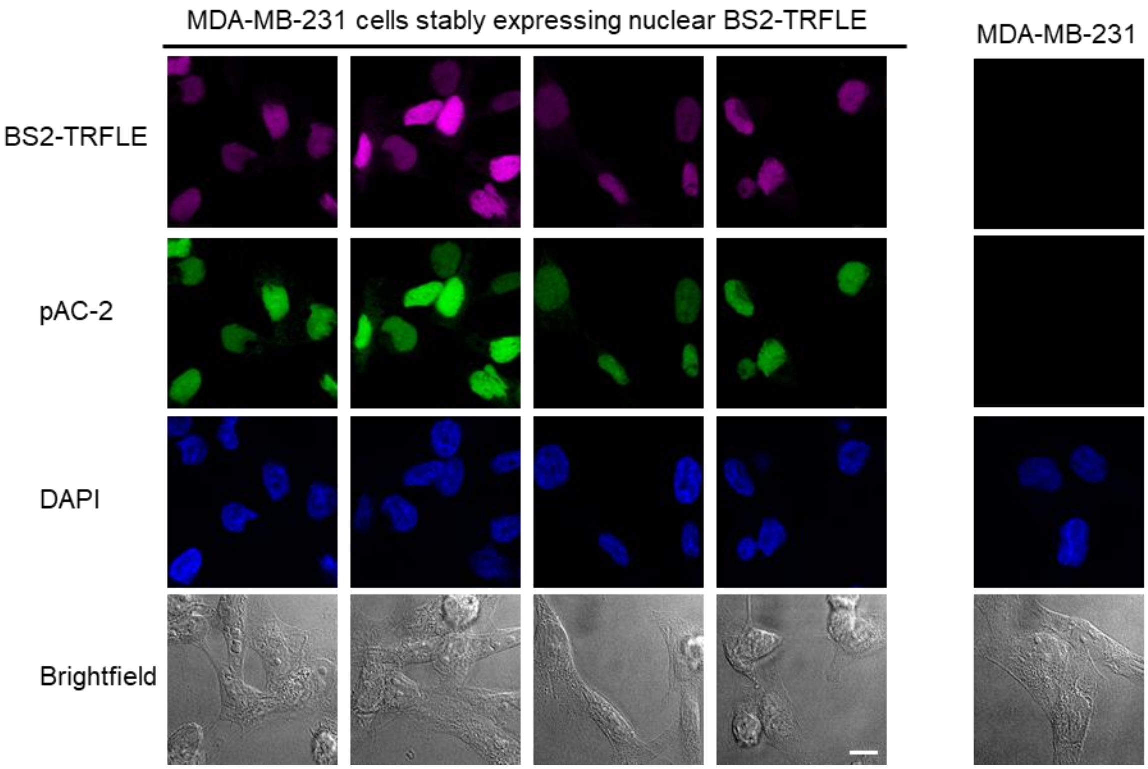

Figure S46. MDA-MB-231 cells stably expressing BS2-TRFLE in the nucleus were treated with 100 µM pAC-2 for 5 min. Cells were fixed, permeabilized, clicked with AF-488 for visualization. BS2-TRFLE was visualized through V5 antibody staining. Scale bar represents 10 µm.

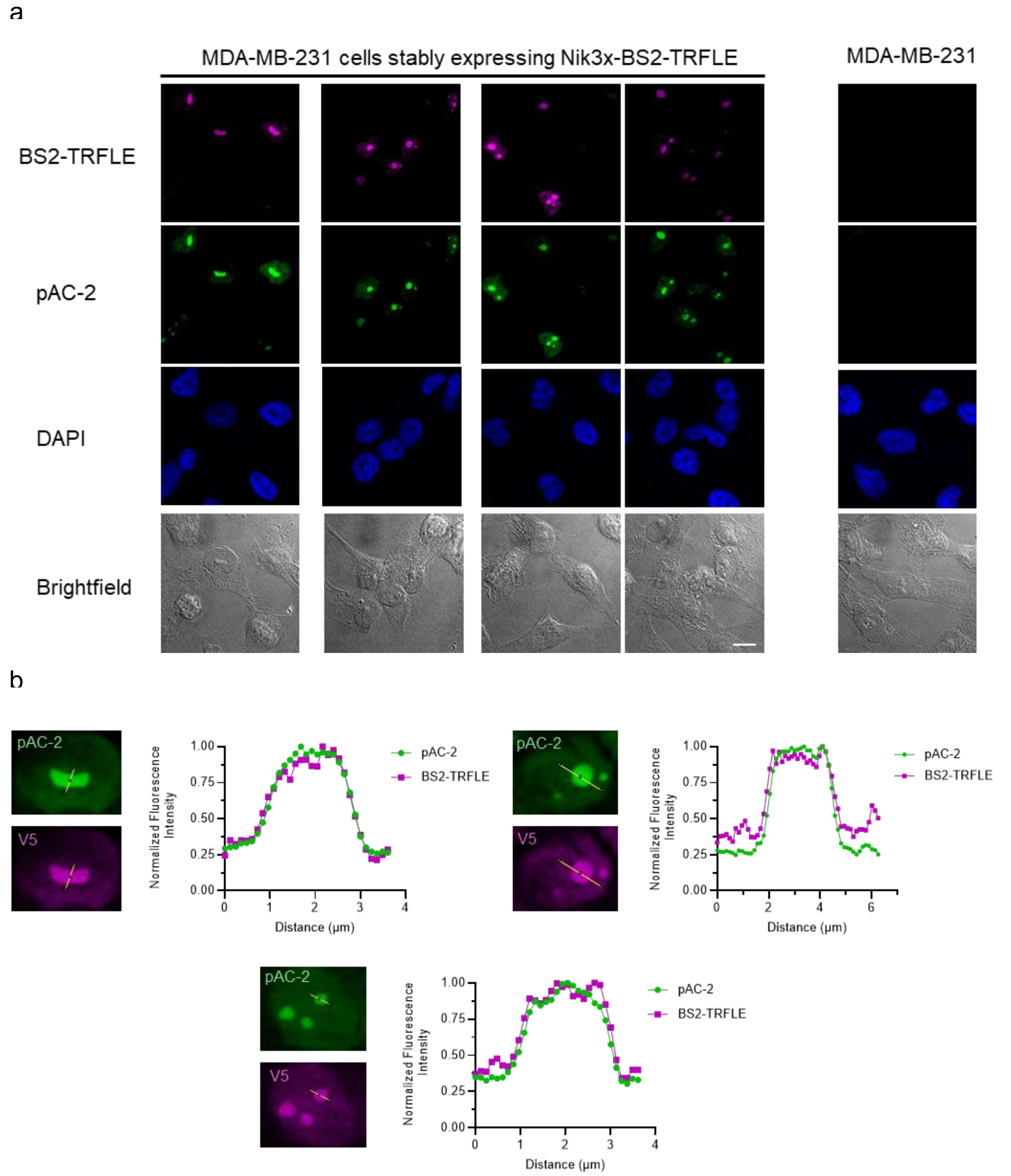

Figure S47. Nucleolar labeling in MDA-MB-231 cells stably expressing BS2-TRFLE in the nucleolus. a) Cells were treated with 100 µM pAC-2 for 5 min. Cells were fixed, permeabilized, clicked with AF-488 for visualization. BS2-TRFLE was visualized through V5 antibody staining. Scale bar represents 10 µm. b) Line plot showing colocalization of nucleolar pAC-2 labeling with BS2-TRFLE-Nik3x expression for three representative regions of interest.

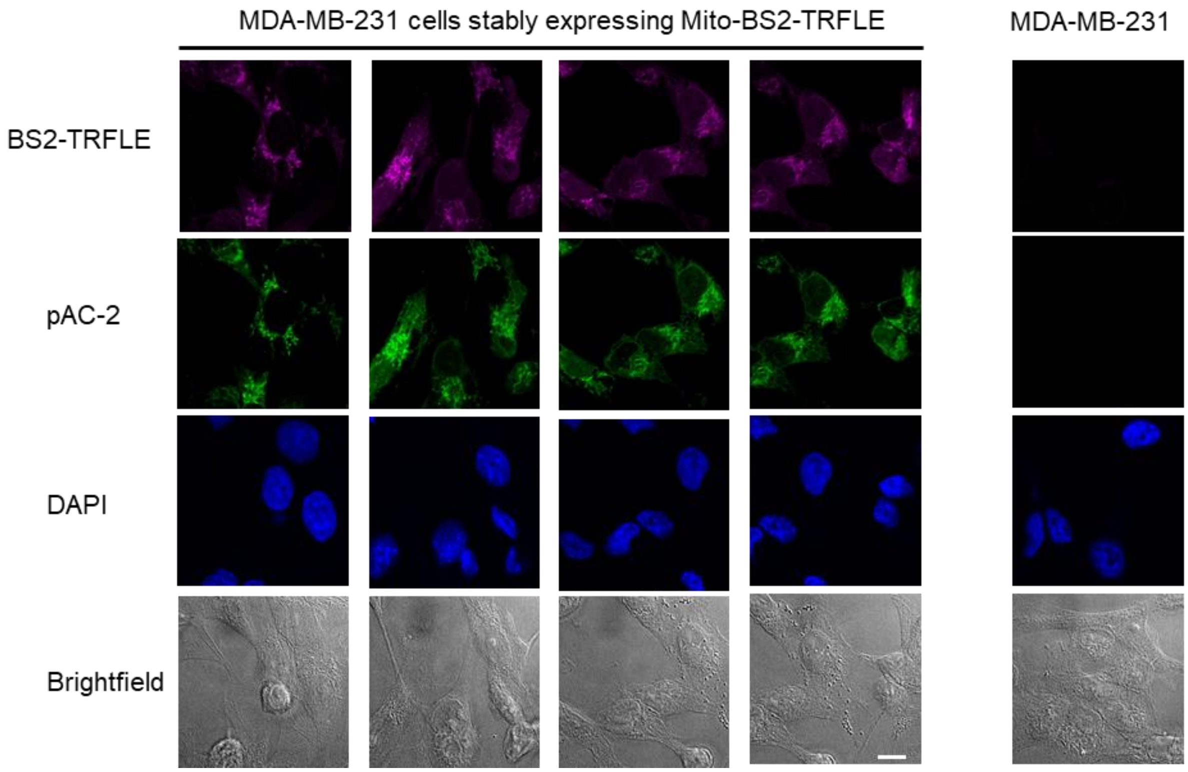

Figure S48. MDA-MB-231 cells stably expressing BS2-TRFLE in the mitochondria were treated with 100 µM pAC-2 for 5 min. Cells were fixed, permeabilized, clicked with AF-488 for visualization. BS2-TRFLE was visualized through V5 antibody staining. Scale bar represents 10 µm.

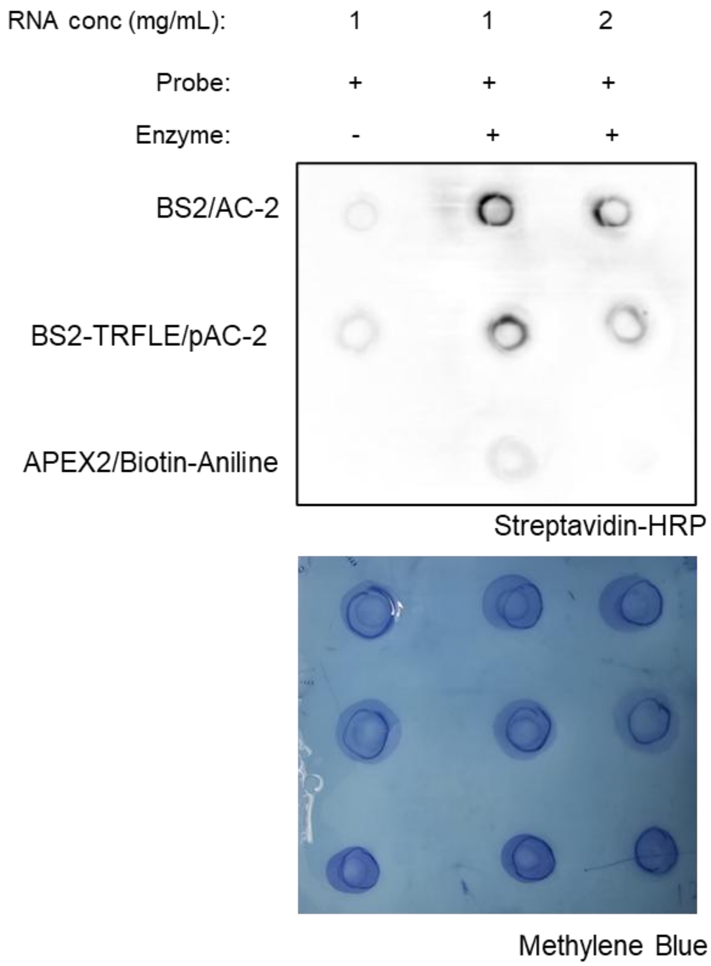

Figure S49. Comparison of *in vitro* RNA labeling efficiency of AC-2/BS2, pAC-2/BS2-TRFLE, and Biotin-Aniline/APEX2 on dot blot. RNA labeling was carried out with 500 nM of the respective enzyme, 0.1 mM of pAC-2, or 0.1 mM of AC-2 or 0.5 mM Biotin-Aniline with 1 mM H_2O_2 at 37 °C for 5 min before quenching. AC-2 and pAC-2-labeled samples were clicked with biotin. 4 µg RNA per sample was then loaded onto PVDF membrane, cross-linked with UV, and visualized with streptavidin-HRP.

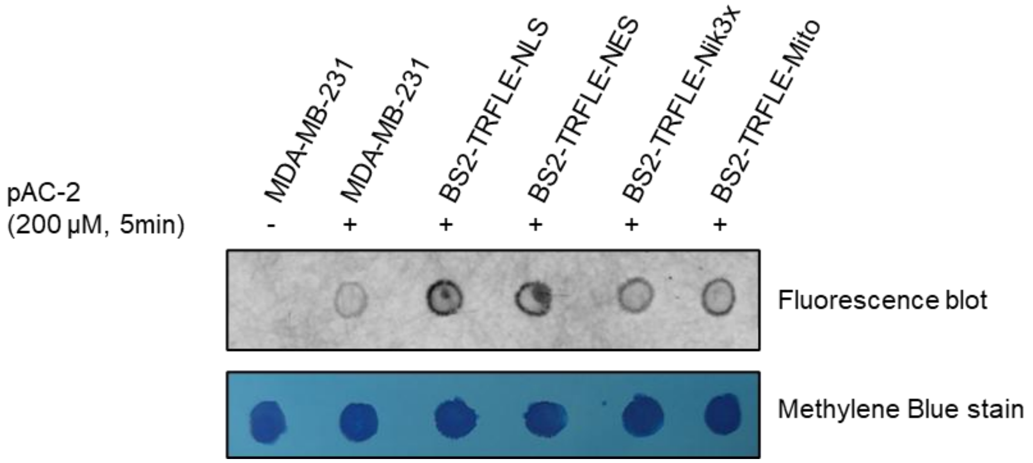

Figure S50. RNA labeling activity of BS2-TRFLE/pAC-2 monitored by dot-blot. MDA-MB-231 cells stably expressing BS2-TRFLE in different compartments were treated with 200 µM pAC-2 probe for 5 min. After lysis and total RNA extraction, click reaction was performed with fluorophore AF488. 5 µg of fluorophore clicked RNA was loaded and crosslinked onto a membrane and subsequently imaged. Methylene blue stain represents total RNA loading for each sample.

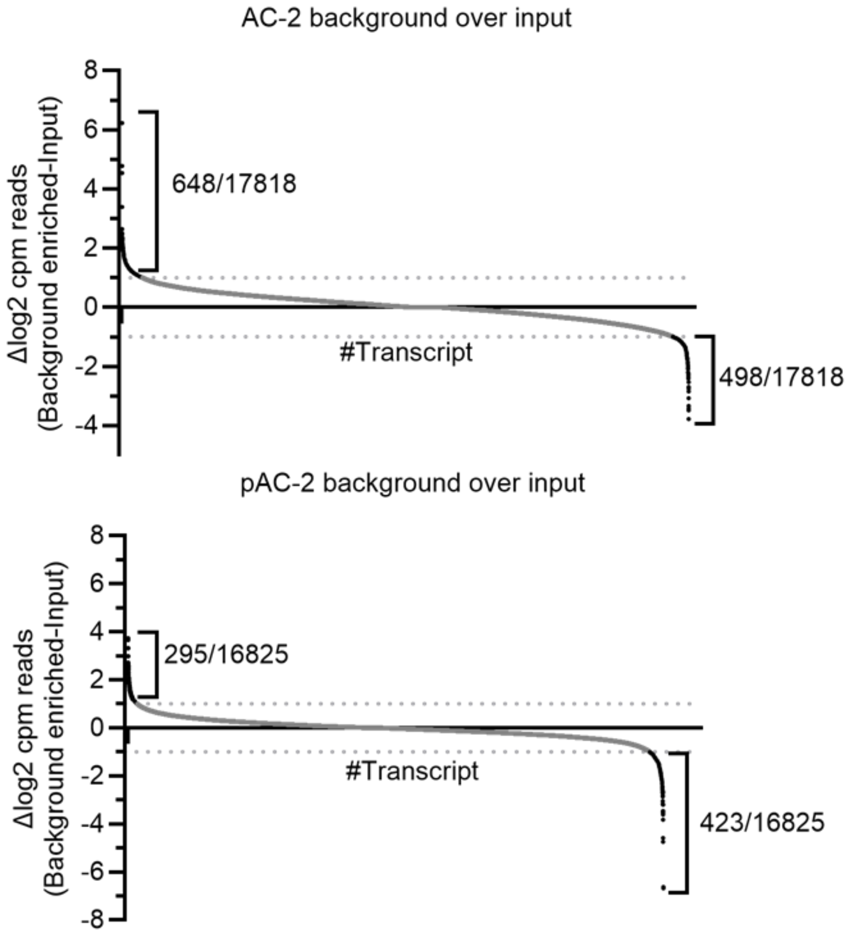

Figure S51. Comparison of enriched transcripts in wildtype MDA-MB-231 cells without BS2 expression. a, b) The difference in Trimmed Mean of M values (TMM)-normalized log_2cpm counts (no BS2-output samples over input samples) of each transcript was plotted. The dotted line represents $\Delta log_2cpm = \pm 1$. The total number of transcripts above the dotted line is presented as a fraction of the total number of transcripts. n=3 for outputs and n=4 for inputs. a) Output from the AC-2 probe. b) Output from pAC-2 probe.

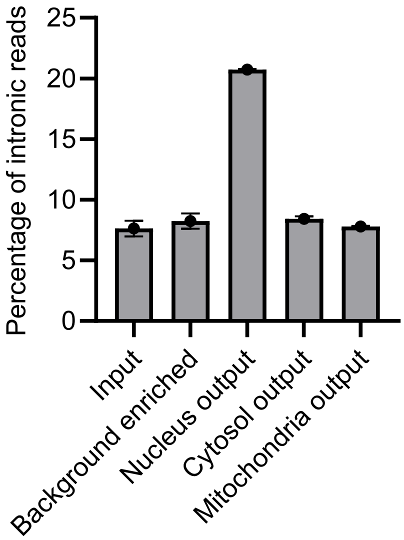

Figure S52. Comparison of intronic reads across different subcellular transcriptomics samples in MDA-MB-231 cells. Numbers are presented as a percentage of total reads that includes exons and intergenic regions. Error bars represent standard deviation, n =4 for input, n=3 for background enriched, n=2 for nucleus, cytosol and mitochondria outputs.

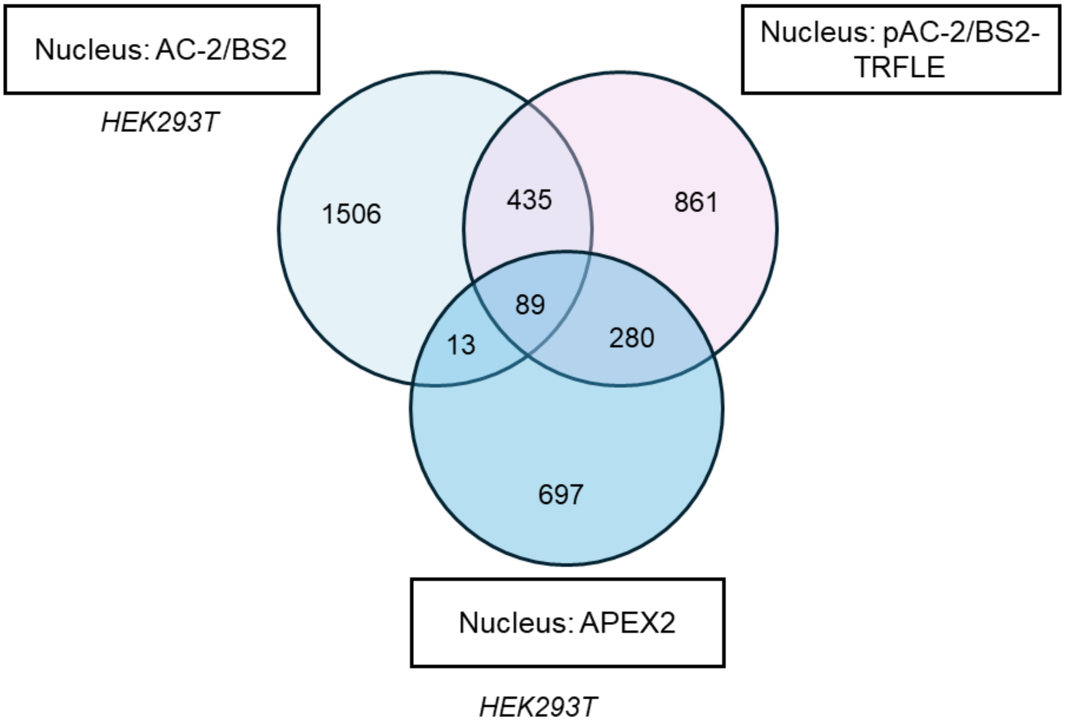

Figure S53. Venn diagram comparing nuclear-enriched datasets of pAC-2/BS2-TRFLE-NLS in MDA-MB-231 cells, AC-2/BS2-NLS labelling in HEK293T cells,[3] and Biotin-Phenol/APEX-NLS in HEK293T cells.[17]

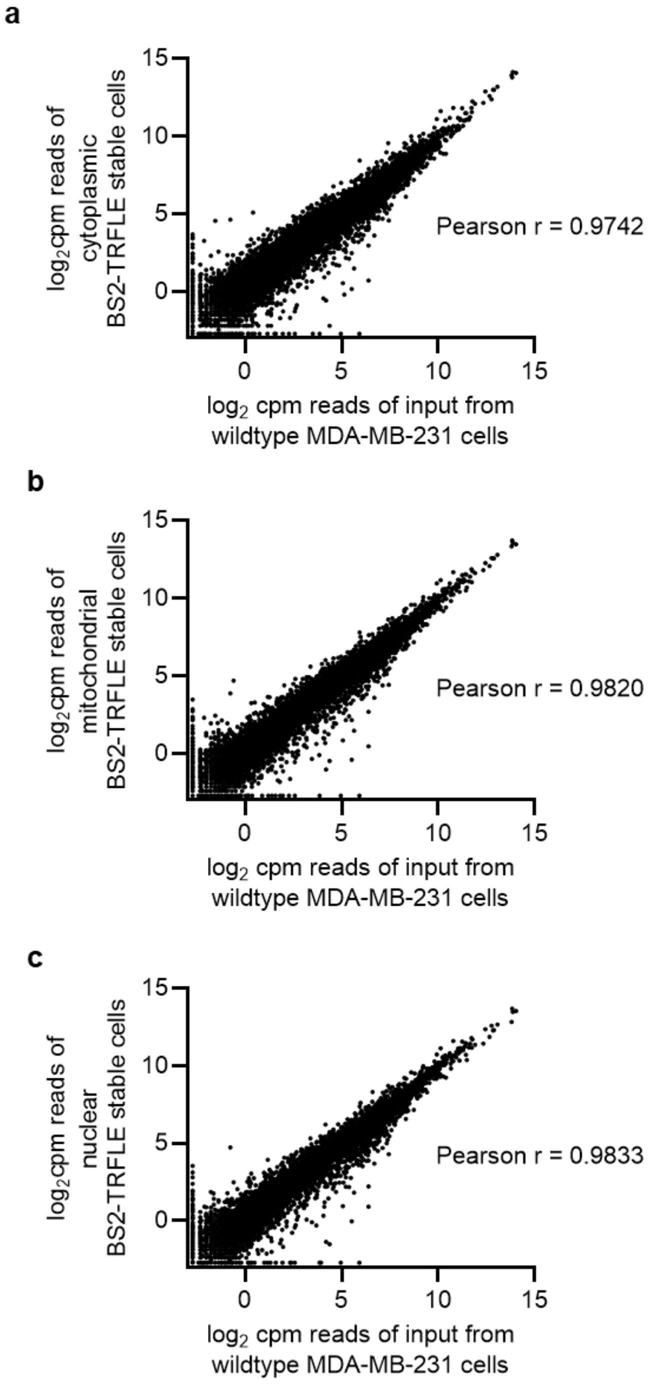

Figure S54. Comparison of cpm (counts per million) reads of different samples post BS2-TRFLE stable cell generation. a, Comparison of filtered and TMM normalized log_2cpm values of inputs from wildtype MDA-MB-231 cells and inputs from cytoplasmic-BS2-TRFLE stable cells. b Comparison of filtered and TMM normalized log_2cpm values of inputs from non-transfected cells and inputs from mitochondrial-BS2-TRFLE stable cells. c, Comparison of filtered and TMM normalized log_2cpm values of inputs from non-transfected cells and inputs from nuclear-BS2-TRFLE stable cells.

SYNTHESIS METHODS AND CHARACTERIZATION

Methods and Materials for Synthesis

For chemical synthesis, reagents and dry solvents were purchased from commercial sources (Sigma Aldrich, Combi-blocks, Thermo Fisher) and used without further purification. Silica gel P60 (SiliCycle, 40–63 µm, 230–400 mesh) was used for column chromatography. Analytical thin-layer chromatography was performed using SiliCycle 60 F254 silica gel (pre-coated sheets, 0.25 mm thick) with detection at 214 nm. Low-resolution-mass spectral analysis and liquid chromatography analysis were carried out on an Advion Expression-L mass spectrometer (Ithaca, NY) with electron spray ionization (ESI) in the positive mode coupled to an Agilent 1220 Infinity LC System with an Agilent Poroshell 120 column (SantaClara, CA). Automated flash column chromatography purification was carried out on a Biotage system Isolera One using SNAP Biotage columns. NMR spectra were recorded on the BRUKER Ascend 400 at the Department of Chemistry NMR Facility, University of Chicago, for ^{1}H-400 MHz and ^{13}C-101 MHz measurements. Chemical shifts are given in parts per million (δ) referenced to TMS (δ = 0.00 ppm ^{1}H-, ^{13}C-NMR). For acyl-coumarin and diphenyl-coumarin, ^{1}H-NMR and ^{13}C{^{1}H}-NMR spectra were recorded on a Bruker Avance III 400 MHz spectrometer. Chemical shifts (δ) are given in parts per million and referenced to TMS. Coupling constants are given in Hertz. High-resolution mass spectra measurements were performed on an Agilent 6224 TOF using a combination of atmospheric pressure chemical ionization and electrospray ionization at the Department of Chemistry Mass Spectrometry Facility, University of Chicago. For acyl-coumarin and diphenyl-coumarin, high-resolution mass spectrometry measurements were obtained on a Thermo Q Exactive™ Plus by the mass spectrometry facility at the University of Wisconsin−Madison.

Synthesis of pCP-coumarin:

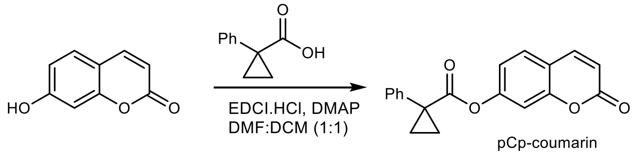

7-hydroxycoumarin (0.5 g, 3.08 mmol, 1.0 eq) was dissolved in the mixture of 10 mL of dry DMF and 10 mL of dry DCM. 1-phenyl-1-cyclopropanecarboxylic acid (0.75 g, 4.62 mmol, 1.5 eq) was added to the reaction mixture, followed by 1-Ethyl-3-(3-dimethylaminopropyl)carbodiimide hydrochloride (EDCI.HCl) (0.855 g, 4.62 mmol, 1.5 eq) and 4-Dimethylaminopyridine (DMAP) (0.564 g, 4.62 mmol, 1.5 eq). The reaction mixture was stirred overnight at room temperature under an N_2 atmosphere. The initial red color of the reaction faded as the reaction progressed. After complete consumption of the starting material, as monitored by TLC, the reaction mixture was diluted with DCM and washed with saturated $NaHCO_3$, followed by washing with brine. The combined organic layers were dried over Na_2SO_4, filtered, concentrated, and purified by column chromatography (Silica; 0-30% EtOAc: hexane) to yield 0.5 g (53%) of pure pCP-coumarin. R_f: 0.65 (Silica; 20% EtOAc: hexane).

^{1}H NMR (400 MHz, $CDCl_3$) δ 7.65 (d, *J* = 9.6 Hz, 1H), 7.49 – 7.27 (m, 6H), 7.05 (d, *J* = 2.2 Hz, 1H), 6.98 (dd, *J* = 8.5, 2.2 Hz, 1H), 6.37 (d, *J* = 9.5 Hz, 1H), 1.81 (q, *J* = 4.1 Hz, 2H), 1.41 (q, *J* = 4.1 Hz, 2H).

^{13}C NMR (101 MHz, $CDCl_3$) δ 172.68, 160.37, 154.62, 153.56, 142.83, 138.47, 130.57, 128.41, 128.35, 127.65, 118.36, 116.53, 116.01, 110.37, 29.26, 17.72.
HRMS: Calculated for $C_{19}H_{14}O_4$ [M+] 306.0892, found 306.0895.

Synthesis of Acyl-Coumarin

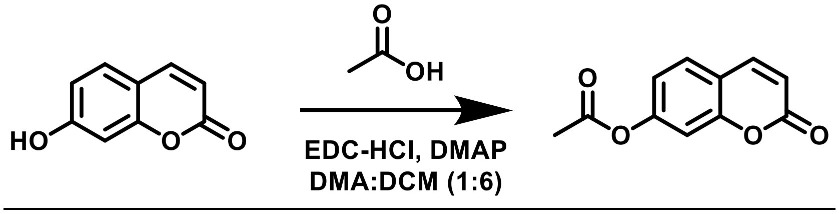

7-hydroxycoumarin (1.23 mmol, 1.0 eq) was dissolved in a mixture of 2.5 mL *N,N*-dimethylacetamide and 15 mL CH_2Cl_2. The coupling reagents 1-ethyl-3-(3-dimethylaminopropyl)carbodiimide hydrochloride (EDC-HCl) (3.69 mmol, 3.0 eq) and 4-dimethylaminopyridine (DMAP) (3.69 mmol, 3.0 eq) were added to the reaction mixture, followed by the dropwise addition of glacial acetic acid (1.85 mmol, 1.5 eq). The reaction mixture was stirred overnight at room temperature under an N_2 atmosphere. The initial brown/red color of the reaction mixture faded to tan as the reaction progressed. The reaction mixture was diluted with CH_2Cl_2 and the organic mixture was washed in a separatory funnel with HCl (1 x 10 mL), washed with saturated $NaHCO_3$ (3 x 10 mL), washed with DI water (1 x 10 mL), and washed with brine (1 x 10 mL) to remove excess water. The organic layer was dried over $MgSO_4$, and the organic solvent was removed under reduced pressure to yield a tan powder. A portion of the tan powder was washed with DI water and was dried overnight under reduced pressure to yield the final off-white powdered product (48 mg, 19% yield).

^1H NMR (400 MHz, DMSO-d6) δ 8.07 (d, J = 9.60 Hz, 1H), 7.77 (d, J = 8.45 Hz, 1H), 7.28 (d, J = 2.19 Hz, 1H), 7.16 (dd, J = 8.45 Hz, 2.19 Hz, 1H), 6.48 (d, J = 9.59 Hz, 1H), 2.31 (s, 3H)

^{13}C NMR (101 MHz, DMSO-d6) δ 169.29, 160.19, 154.56, 153.36, 144.31, 129.82, 119.16, 117.14, 116.03, 110.61, 21.35
HRMS: Calculated for $C_{11}H_8O_4$ [M+H]+ 205.0495, found 205.0494.

Synthesis of Diphenyl-Coumarin

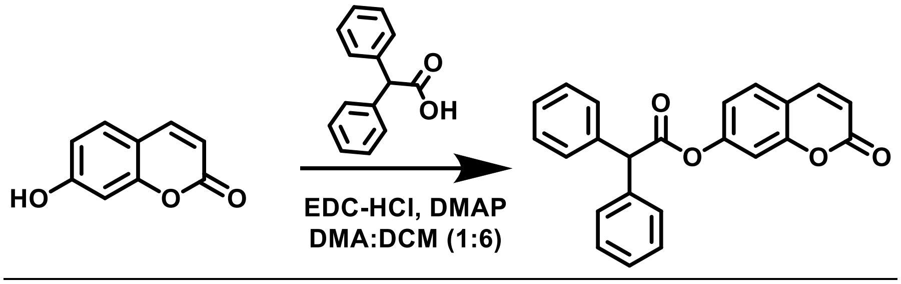

7-hydroxycoumarin (1.56 mmol, 1.0 eq) was dissolved in a mixture of 2.5 mL *N,N*-dimethylacetamide and 15 mL CH_2Cl_2. The coupling reagents 1-ethyl-3-(3-dimethylaminopropyl)carbodiimide hydrochloride (EDC-HCl) (4.68 mmol, 3.0 eq) and 4-dimethylaminopyridine (DMAP) (4.68 mmol, 3.0 eq) were added to the reaction mixture, followed by the addition of diphenylacetic acid (1.72 mmol, 1.1 eq). The reaction mixture was stirred overnight at room temperature under an N_2 atmosphere. The initial brown/red color of the reaction mixture faded to tan as the reaction progressed. The reaction mixture was diluted with CH_2Cl_2 and the organic mixture was washed in a separatory funnel with HCl (1 x 10 mL), washed with saturated $NaHCO_3$ (3 x 10 mL), washed with DI water (1 x 10 mL), and washed with brine (1 x 10 mL) to remove excess water. The organic solvent was removed under reduced pressure to yield the final off-white solid product (324 mg, 59% yield).

^{1}H NMR (400 MHz, DMSO-d6) δ 8.07 (d, J = 9.57 Hz, 1H), 7.77 (d, J = 8.44 Hz, 1H), 7.43 (m, 8H), 7.31 (m, 3H), 7.12 (dd, J = 8.40 Hz, 2.23 Hz, 1H), 6.48 (d, J = 9.59 Hz, 1H), 5.58 (s, 1H).

^{13}C NMR (101 MHz, DMSO-d6) δ 171.02, 160.11, 154.61, 153.13, 144.24, 138.87, 130.02, 129.21, 129.08, 127.82, 118.78, 117.40, 116.20, 110.36, 56.04.
HRMS: Calculated for $C_{23}H_{16}O_4$ [M+Na]+ 379.0941, found 379.0934.

Synthesis of Fluorescein-AM esters:

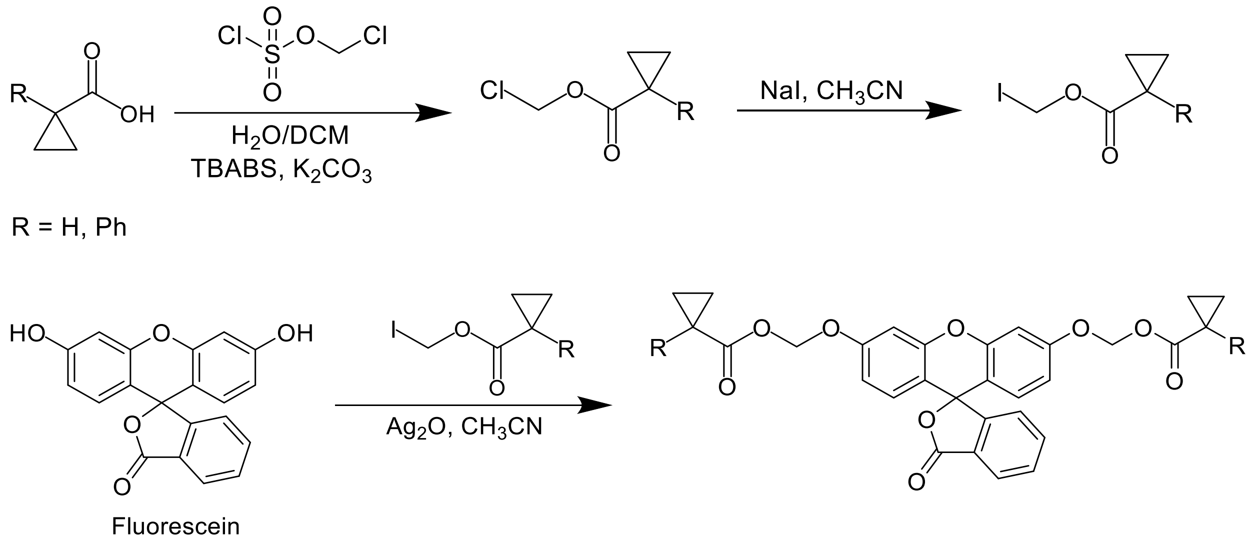

All fluorescein-AM ester probes were synthesized following published protocols[25] as shown in the scheme above.

mCP-FL: ((3-oxo-3H-spiro[isobenzofuran-1,9'-xanthene]-3',6'-diyl)bis(oxy))bis(methylene) bis(1-methylcyclopropane-1-carboxylate). Yield: 93.7 mg (56%).

^{1}H NMR (400 MHz, CDCl3) δ 7.96 (dt, J = 7.4, 1.0 Hz, 1H), 7.66 – 7.52 (m, 2H), 7.10 (dt, J = 7.6, 1.0 Hz, 1H), 6.87 (dd, J = 1.9, 0.9 Hz, 2H), 6.72 – 6.61 (m, 4H), 5.69 (d, J = 0.9 Hz, 4H), 1.26 – 1.14 (m, 11H), 0.71 – 0.63 (m, 4H).

^{13}C NMR (101 MHz, CDCl3) δ 174.82, 169.23, 158.54, 152.90, 152.27, 135.13, 129.89, 129.38, 126.70, 125.16, 123.93, 113.22, 112.64, 103.55, 85.09, 82.52, 19.17, 18.69, 17.40.

pCP-FL: ((3-oxo-3H-spiro[isobenzofuran-1,9'-xanthene]-3',6'-diyl)bis(oxy))bis(methylene) bis(2-ethylbutanoate). Yield: 145.1 mg (71%).

^{1}H NMR (400 MHz, CDCl3) δ 8.02 (dt, J = 7.4, 1.0 Hz, 1H), 7.71 – 7.58 (m, 2H), 7.37 – 7.17 (m, 10H), 7.14 (dt, J = 7.6, 1.0 Hz, 1H), 6.75 (d, J = 2.5 Hz, 2H), 6.64 (d, J = 8.8 Hz, 2H), 6.56 (dd, J = 8.8, 2.5 Hz, 2H), 5.67 (s, 4H), 1.71 – 1.57 (m, 4H), 1.24 (p, J = 3.3 Hz, 4H).

^{13}C NMR (101 MHz, CDCl3) δ 173.44, 169.30, 158.44, 153.02, 152.15, 138.60, 135.12, 130.53, 129.92, 129.23, 128.27, 127.47, 126.66, 125.20, 123.89, 113.25, 112.82, 103.96, 85.36, 82.53, 29.17, 17.24.

FBM108-((3-oxo-3H-spiro[isobenzofuran-1,9'-xanthene]-3',6'-diyl)bis(oxy))bis(methylene)bis(2,2-dimethylbutanoate): Yield (pure fraction): 3.8 mg (3.8%), MW-588.65

^{1}H NMR (400 MHz, CDCl3) δ 8.03 (dd, 1H), 7.72 – 7.61 (m, 2H), 7.17 (d, 1H), 6.96 (t, 2H), 6.73 (d, 4H), 5.79 (d, 4H), 1.18 (s, 18H).
HRMS: Calculated for $C_{34}H_{36}O_9$ [M+] 588.2359, found 588.2312

FBM112: ((3-oxo-3H-spiro[isobenzofuran-1,9'-xanthene]-3',6'-diyl)bis(oxy))bis(methylene) bis(1-ethylcyclopropane-1-carboxylate). Yield (Pure fraction): 11.2 mg (15%), MW: 584.62,

^{1}H NMR (400 MHz, CDCl3) δ 8.07 – 8.00 (m, 1H), 7.73 – 7.59 (m, 2H), 7.18 (dt, 1H), 6.94 (dd, 2H), 6.73 (t, 4H), 5.81 – 5.71 (m, 4H), 1.28 – 1.22 (m, 8H), 0.98 (t, 6H), 0.79 – 0.72 (m, 4H).

FBM114: ((3-oxo-3H-spiro[isobenzofuran-1,9'-xanthene]-3',6'-diyl)bis(oxy))bis(methylene) bis(2-ethylbutanoate). Yield (pure fraction): 4.5 mg (2.3%). MW: 588.65

^{1}H NMR (400 MHz, CDCl3) δ 8.03 (dd, 1H), 7.73 – 7.58 (m, 2H), 7.19 – 7.12 (m, 1H), 6.96 (dd, 2H), 6.80 – 6.66 (m, 4H), 5.80 (s, 4H), 2.28 (tt, 2H), 1.72 – 1.56 (m, 4H), 0.86 (t, 12H)

Synthesis of SP-d5:

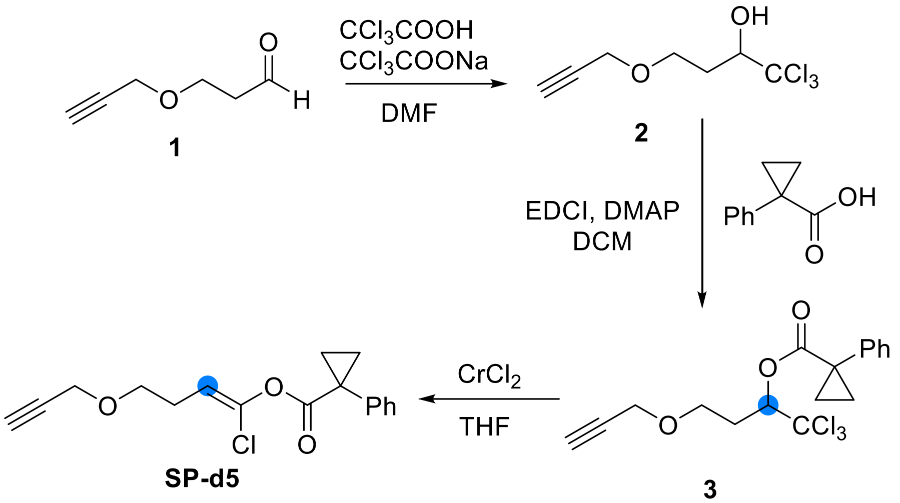

Synthesis of phenyl ester **3: 1,1,1-trichloro-4-(prop-2-yn-1-yloxy)butan-2-yl 1-phenylcyclopropane-1-carboxylate. 3** was synthesized using published protocols[3] as shown in the scheme above, without column purification of **2.** Yield: 300 mg (38%).

^{1}H NMR (400 MHz, $CDCl_3$) δ 7.43 – 7.24 (m, 5H), 5.64 (dd, *J* = 10.1, 2.1 Hz, 1H), 4.23 – 4.07 (m, 2H), 3.64 (ddd, *J* = 9.4, 5.8, 4.9 Hz, 1H), 3.46 (td, *J* = 9.1, 4.9 Hz, 1H), 2.50 – 2.38 (m, 2H), 1.95 (ddt, *J* = 14.9, 9.9, 4.9 Hz, 1H), 1.82 – 1.69 (m, 2H), 1.39 – 1.25 (m, 2H).

^{13}C NMR (101 MHz, $CDCl_3$) δ 172.90, 138.68, 130.60, 128.14, 127.37, 99.90, 79.42, 78.80, 74.66, 65.58, 58.30, 30.81, 29.09, 16.99, 16.50.

HRMS: Calculated for C17H17Cl3O3 [M+] 374.024; found 374.0255.

SP-d5: (Z)-1-chloro-4-(prop-2-yn-1-yloxy)but-1-en-1-yl 1-phenylcyclopropane-1-carboxylate

^{1}H NMR (400 MHz, $CDCl_3$) δ 7.35 – 7.17 (m, 5H), 5.34 (t, *J* = 7.2 Hz, 1H), 4.05 (d, *J* = 2.4 Hz, 2H), 3.48 (t, *J* = 6.5 Hz, 2H), 2.44 – 2.26 (m, 3H), 1.65 (q, *J* = 4.1 Hz, 2H), 1.26 (q, *J* = 3.9 Hz, 2H).

^{13}C NMR (101 MHz, $CDCl_3$) δ 171.73, 138.16, 135.63, 130.50, 128.35, 127.60, 114.69, 79.57, 74.48, 68.09, 58.08, 28.88, 27.92, 17.55.

HRMS: Calculated for $C_{17}H_{17}ClO_3$ [M+] 304.0866, found 304.0858

Synthesis of pAC-2:

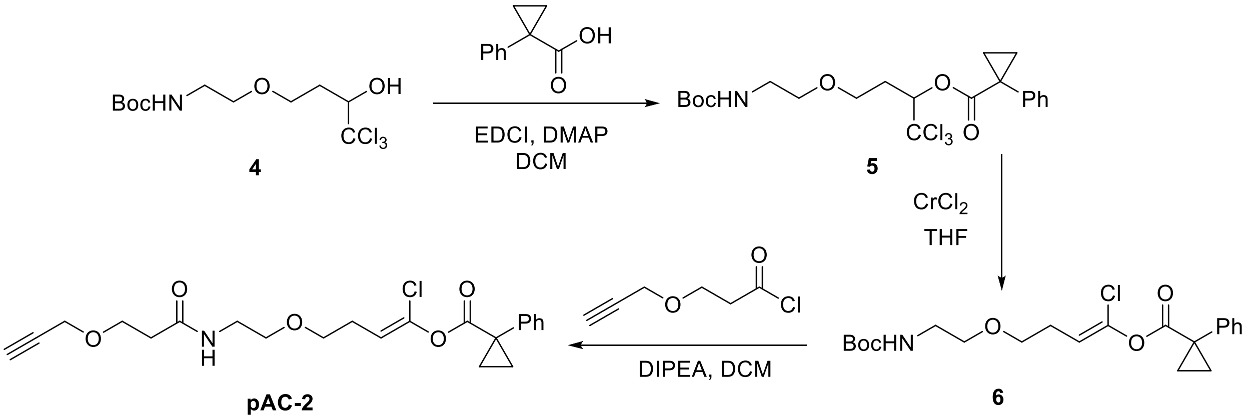

pAC-2 was synthesized following a procedure analogous to the AC-2 synthesis[3] by replacing 1-methylcyclopropyl acid with 1-phenylcycloproyl acid, as shown in the scheme above.

Compound 5 4-(2-((tert-butoxycarbonyl)amino)ethoxy)-1,1,1-trichlorobutan-2-yl 1-phenylcyclopropane-1-carboxylate. Yield: 380 mg (70.56%)

^{1}H NMR (400 MHz, $CDCl_3$) δ 7.34 – 7.14 (m, 5H), 5.59 (dd, J = 10.1, 2.1 Hz, 1H), 5.07 (s, 1H), 3.52 – 3.37 (m, 2H), 3.36 – 3.12 (m, 4H), 2.42 – 2.21 (m, 1H), 1.84 – 1.57 (m, 3H), 1.38 (s, 9H), 1.30 – 1.15 (m, 2H).

^{13}C NMR (101 MHz, $CDCl_3$) δ 173.20, 156.11, 138.55, 130.60, 128.14, 127.38, 100.01, 79.16, 78.67, 70.39, 66.28, 30.68, 29.13, 28.45, 17.32, 16.47.

Compound 6 (Z)-4-(2-((tert-butoxycarbonyl)amino)ethoxy)-1-chlorobut-1-en-1-yl 1-phenylcyclopropane-1-carboxylate. Yield: 511 mg (57%)

^{1}H NMR (400 MHz, $CDCl_3$) δ 7.35 – 7.16 (m, 5H), 5.31 (t, J = 7.3 Hz, 1H), 4.84 (s, 1H), 3.45 – 3.35 (m, 4H), 3.21 (q, J = 5.2 Hz, 2H), 2.31 (q, J = 6.7 Hz, 2H), 1.65 (t, J = 3.5 Hz, 2H), 1.37 (s, 9H), 1.26 (q, J = 4.1 Hz, 2H).

^{13}C NMR (101 MHz, $CDCl_3$) δ 171.77, 155.99, 138.14, 135.60, 130.49, 128.35, 127.60, 114.84, 79.25, 69.80, 69.05, 53.44, 28.88, 28.43, 28.07, 17.59.

pAC-2 ((Z)-1-chloro-4-(2-(3-(prop-2-yn-1-yloxy)propanamido)ethoxy)but-1-en-1-yl 1-phenylcyclopropane-1-carboxylate): Yield: 77 mg (15%).

^{1}H NMR (400 MHz, $CDCl_3$) δ 7.36 – 7.15 (m, 5H), 6.39 (s, 1H), 5.31 (t, J = 7.4 Hz, 1H), 4.06 (d, J = 2.4 Hz, 2H), 3.69 (q, J = 5.9 Hz, 2H), 3.45 – 3.38 (m, 4H), 3.35 (q, J = 4.7 Hz, 2H), 2.47 – 2.27 (m, 5H), 1.66 (m, 2H), 1.27 (m, 2H).

^{13}C NMR (101 MHz, $CDCl_3$) δ 171.97, 171.12, 138.09, 135.62, 130.51, 128.36, 127.64, 115.18, 79.45, 74.73, 69.45, 69.32, 68.94, 66.09, 58.28, 39.24, 36.61, 28.91, 28.07, 17.57.
HRMS: Calculated for $C_{22}H_{26}ClNO_5$ [M+] 419.15, found 419.1537

pCP-Coumarin

^{1}H NMR

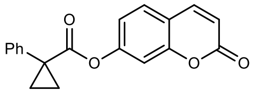

pCp-coumarin

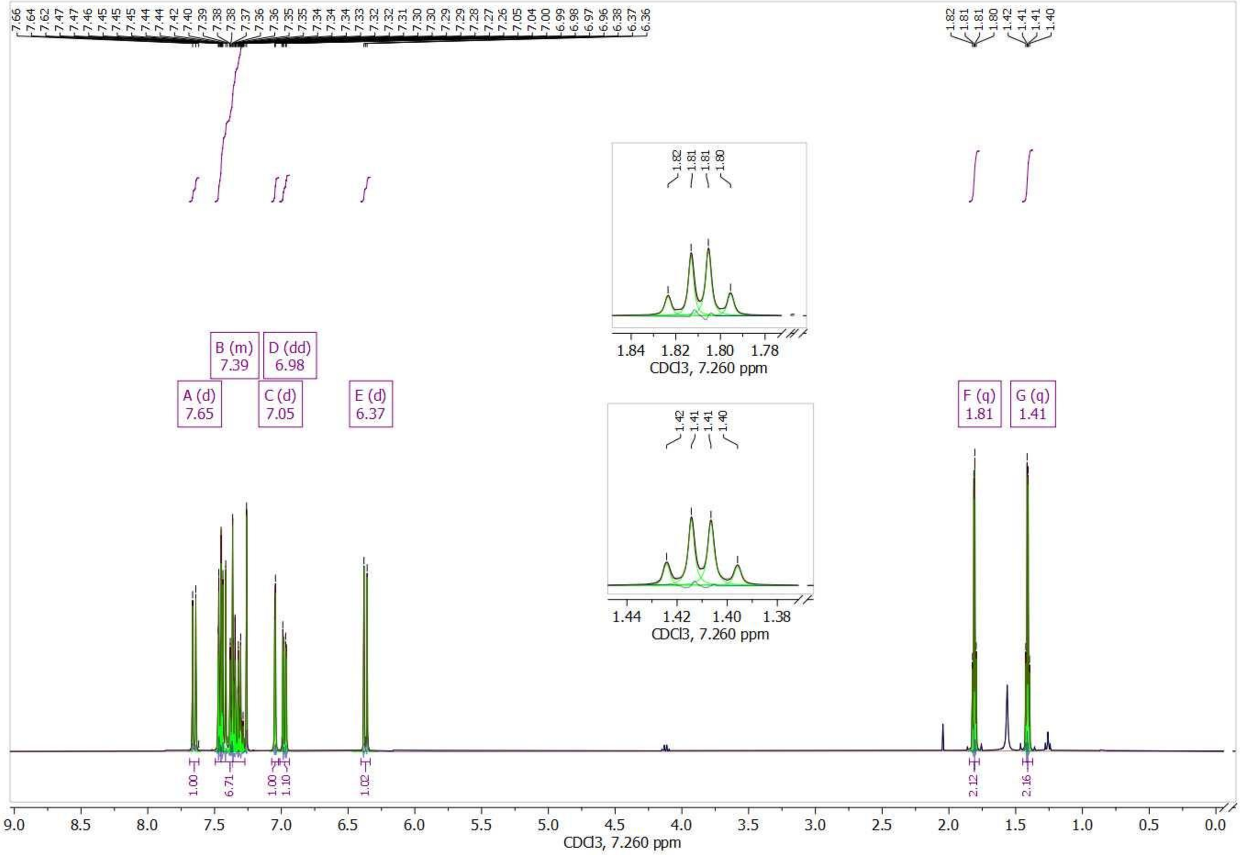

pCP-Coumarin

^{13}C NMR

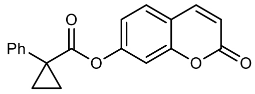

pCp-coumarin

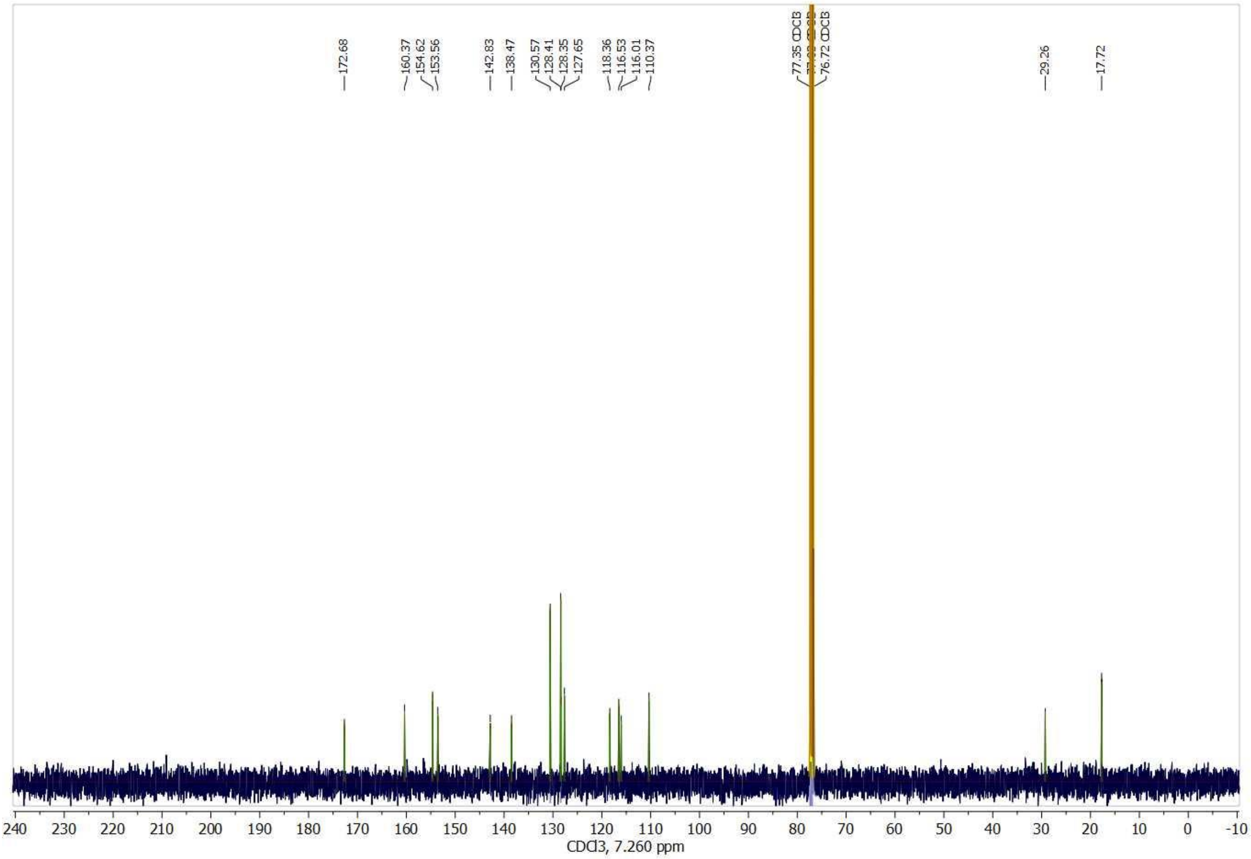

Acyl-Coumarin

^{1}H NMR

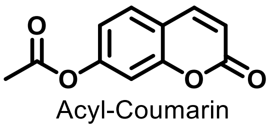

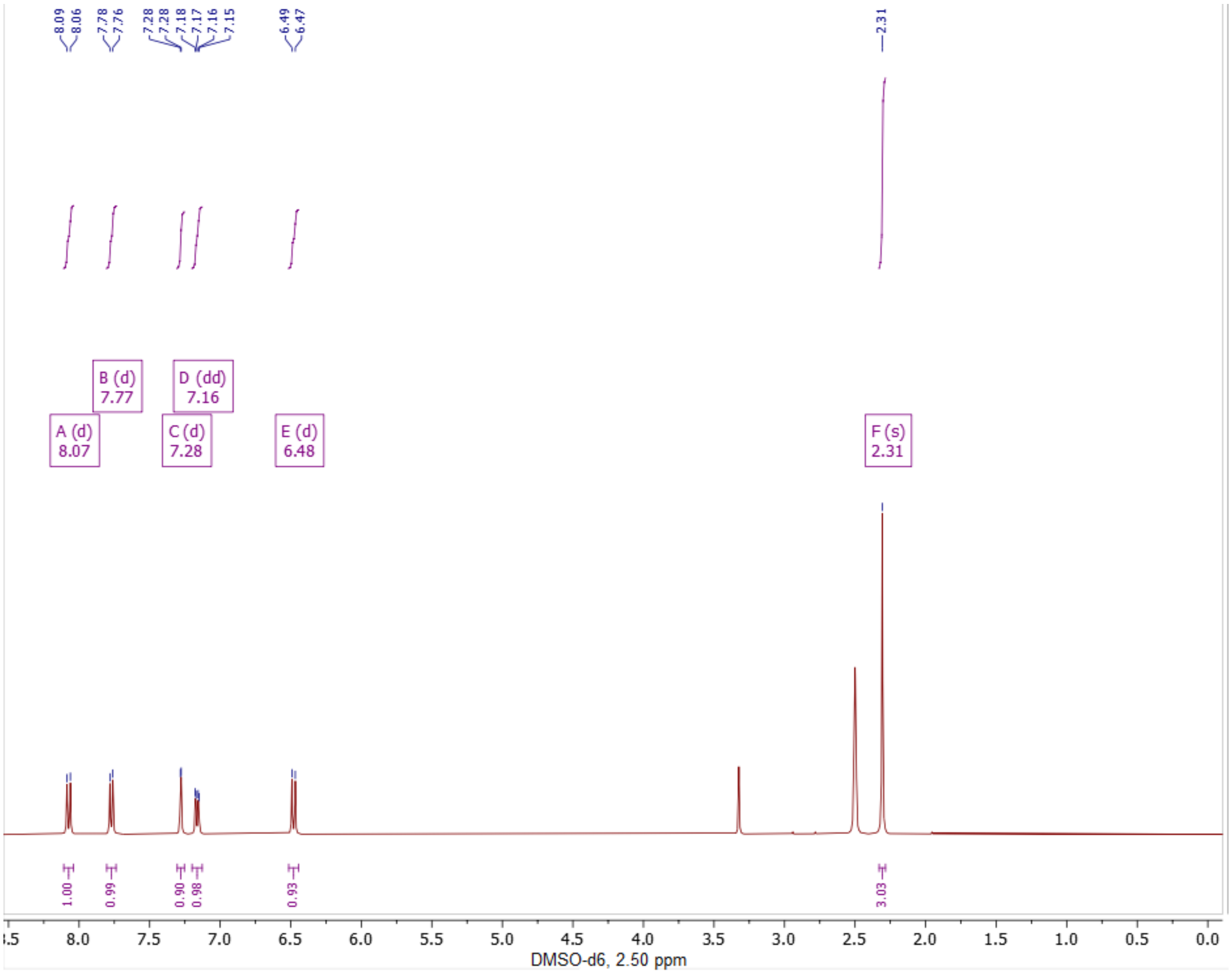

Acyl-Coumarin

^{13}C NMR

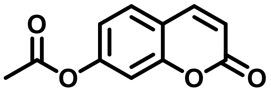

Acyl-Coumarin

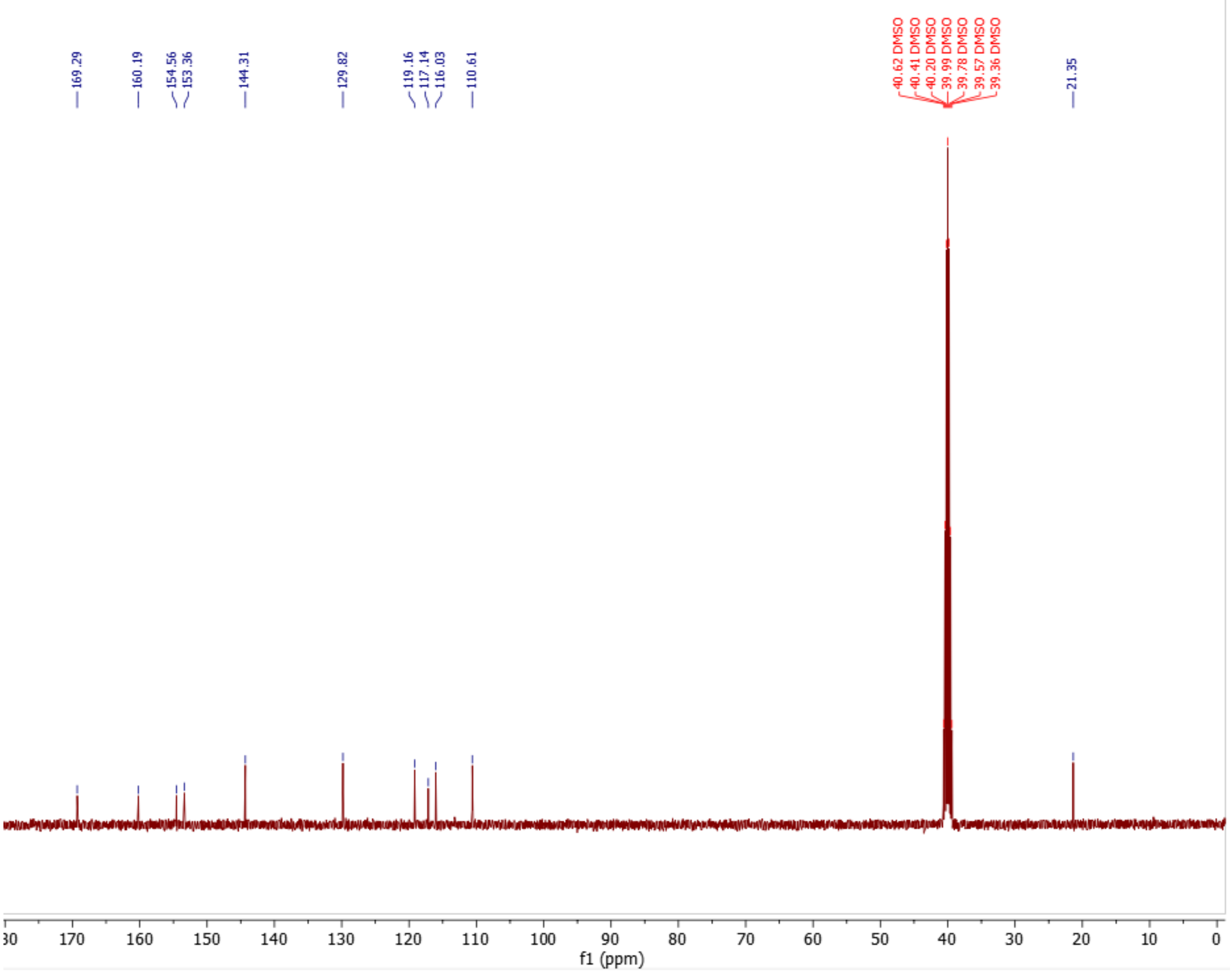

Diphenyl-Coumarin

^{1}H NMR

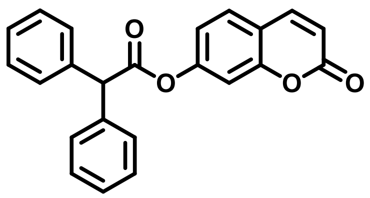

Diphenyl-Coumarin

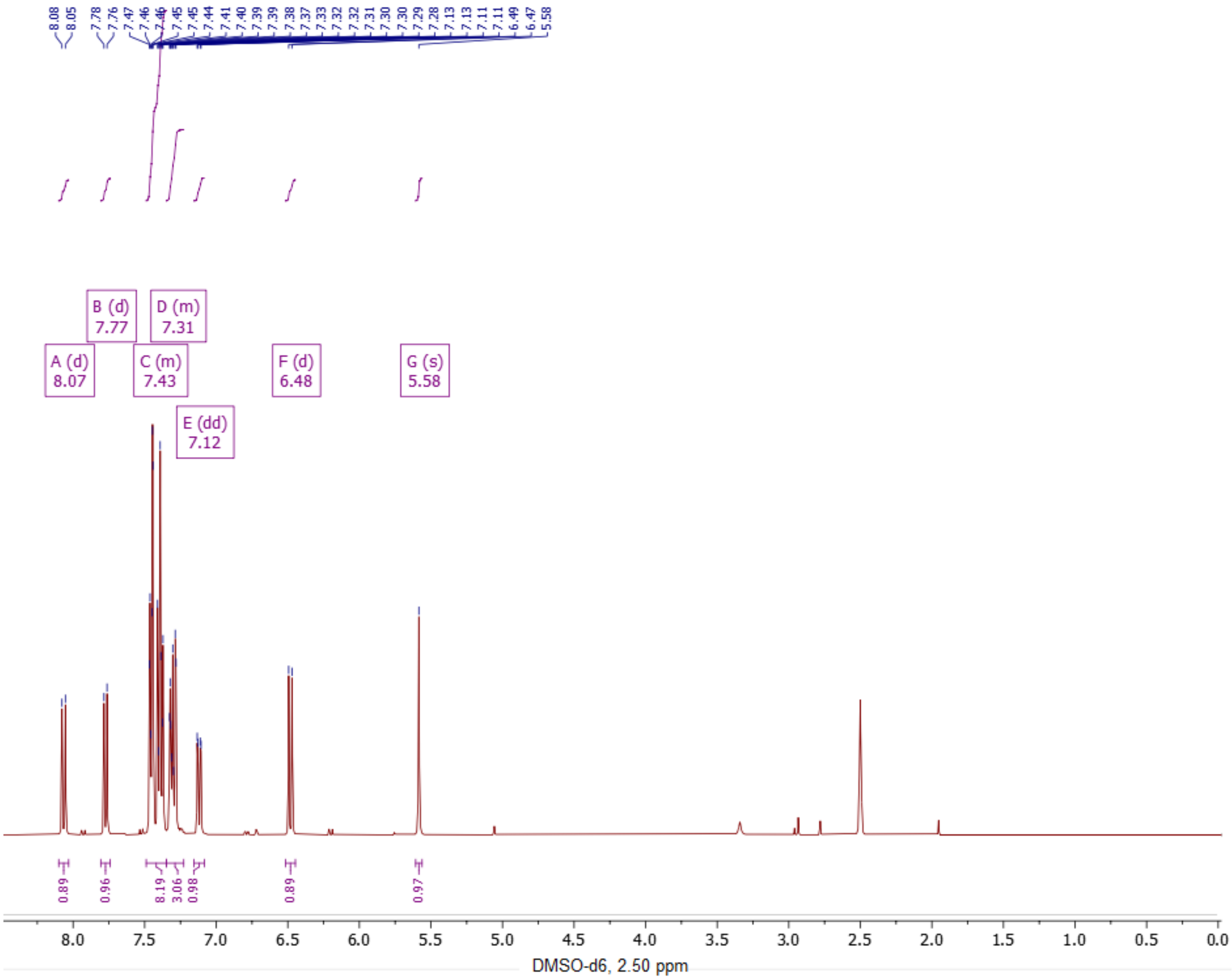

Impurity peak at 3.34 ppm corresponds to residual water. Impurity peaks at 2.93, 2.78, and 1.95 correspond to residual reaction solvent *N,N*-dimethylacetamide. Impurity peaks in the aromatic region correspond to 7-hydroxycoumarin—integration indicates <4% abundance.

Diphenyl-Coumarin

^{13}C NMR

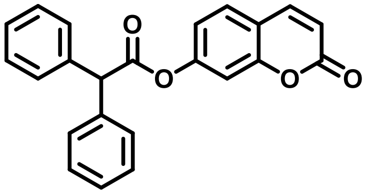

Diphenyl-Coumarin

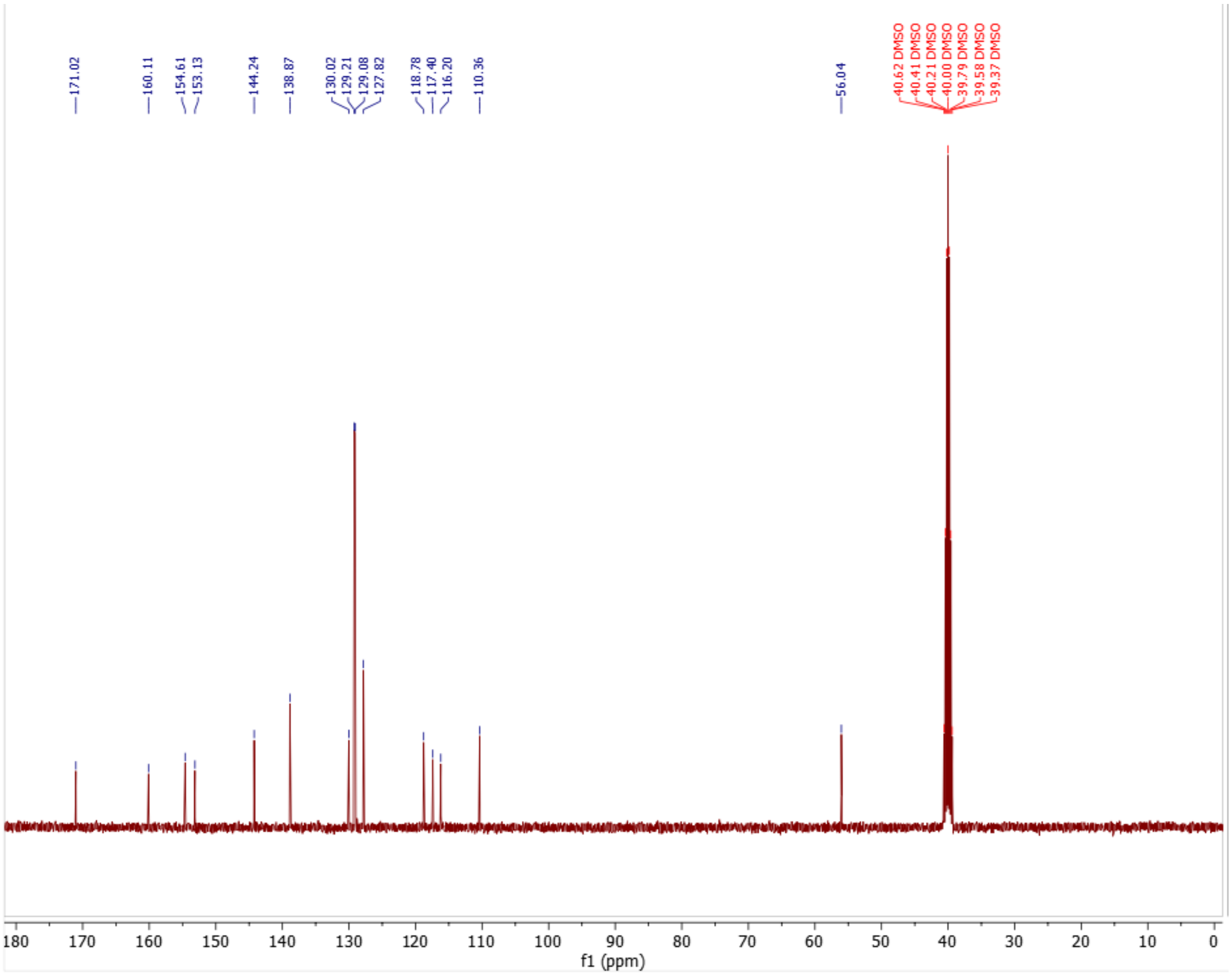

Compound 1: mCP-Fluorescein

^{1}H NMR spectrum

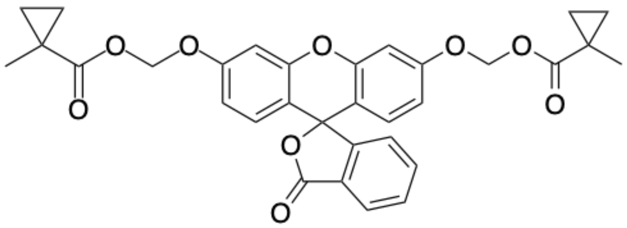

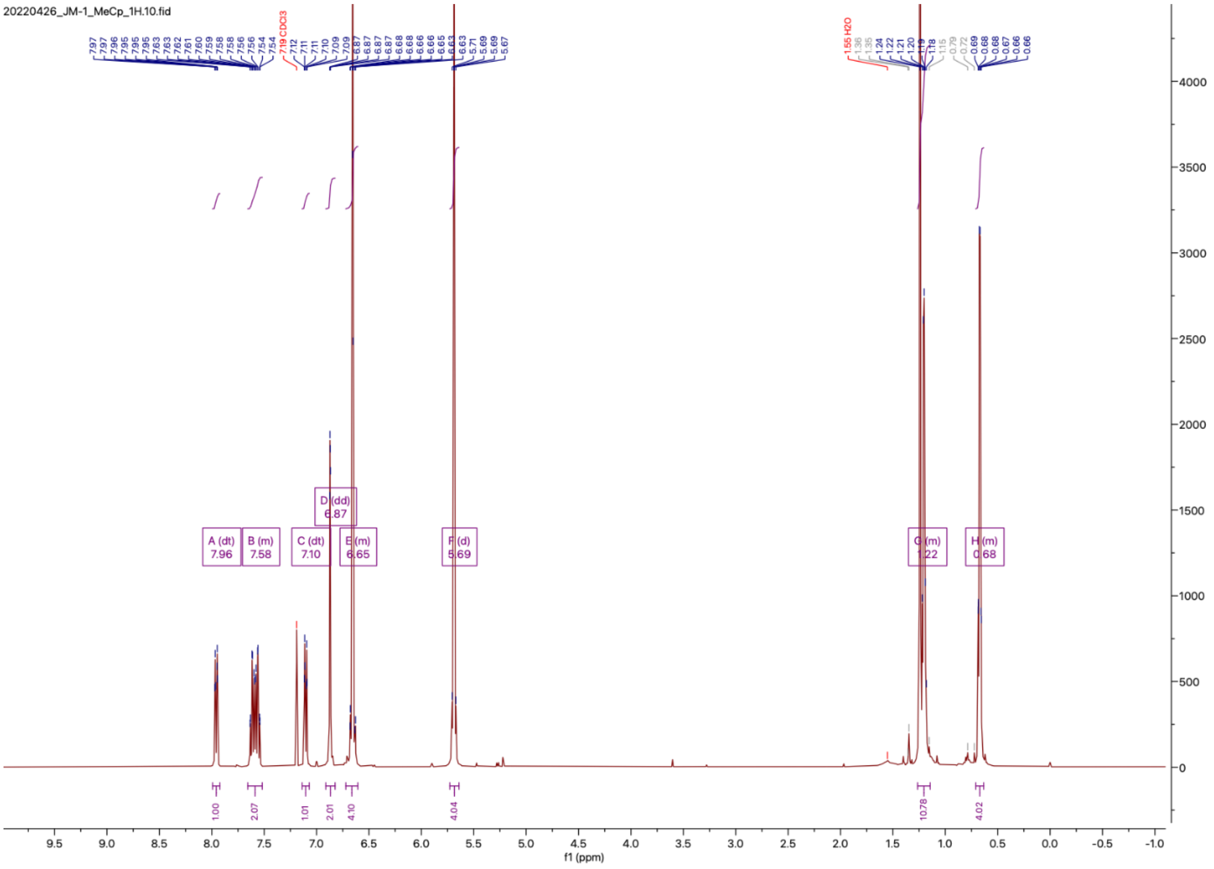

Compound 1: mCP-Fluorescein

^{13}C NMR spectrum

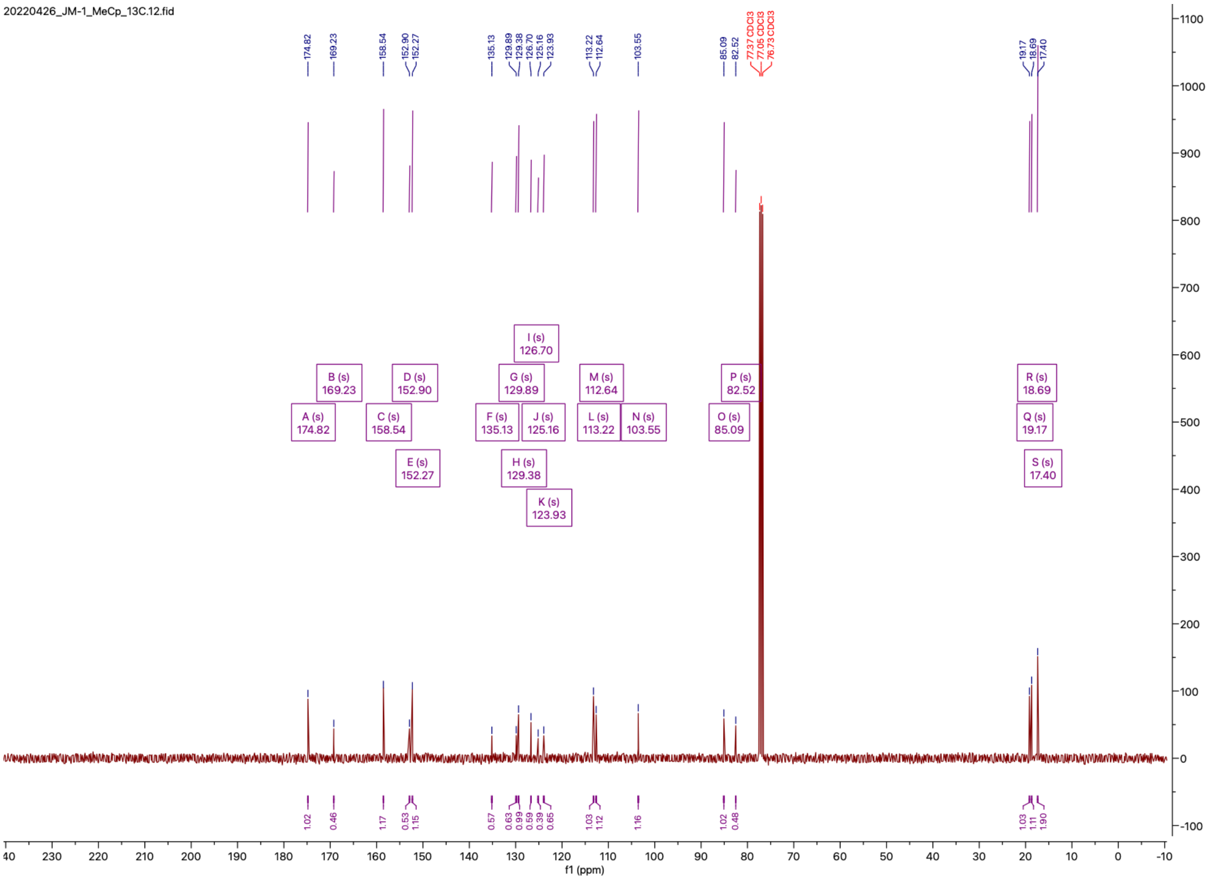

Compound 2: pCP-Fluorescein

^{1}H NMR spectrum

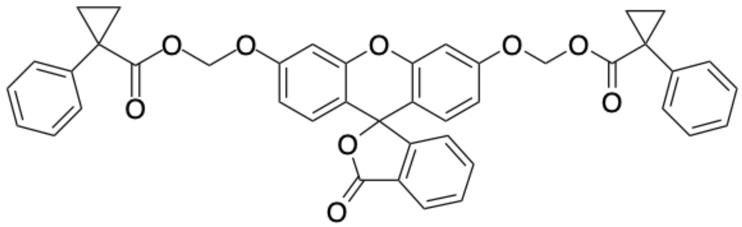

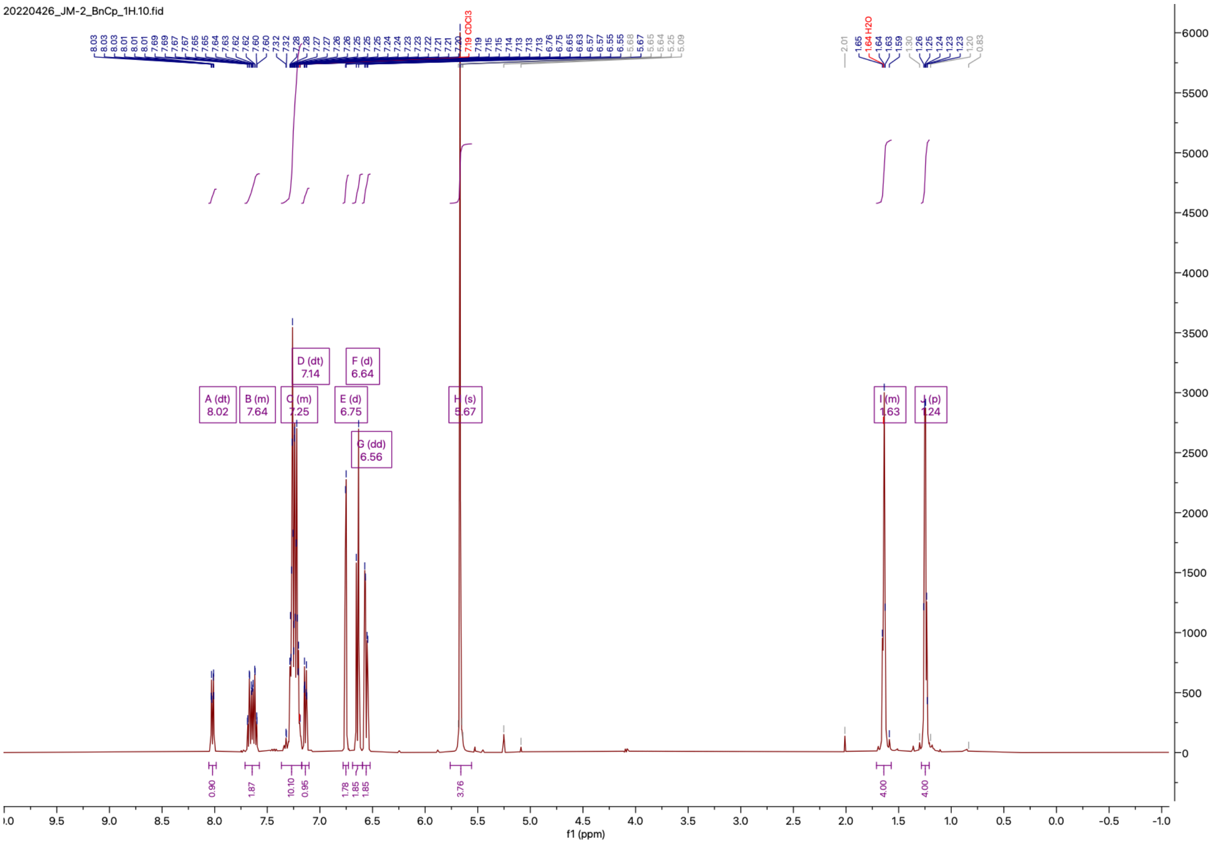

Compound 2: pCP-Fluorescein

^{13}C NMR spectrum

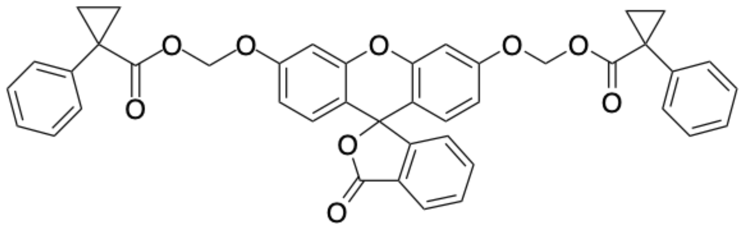

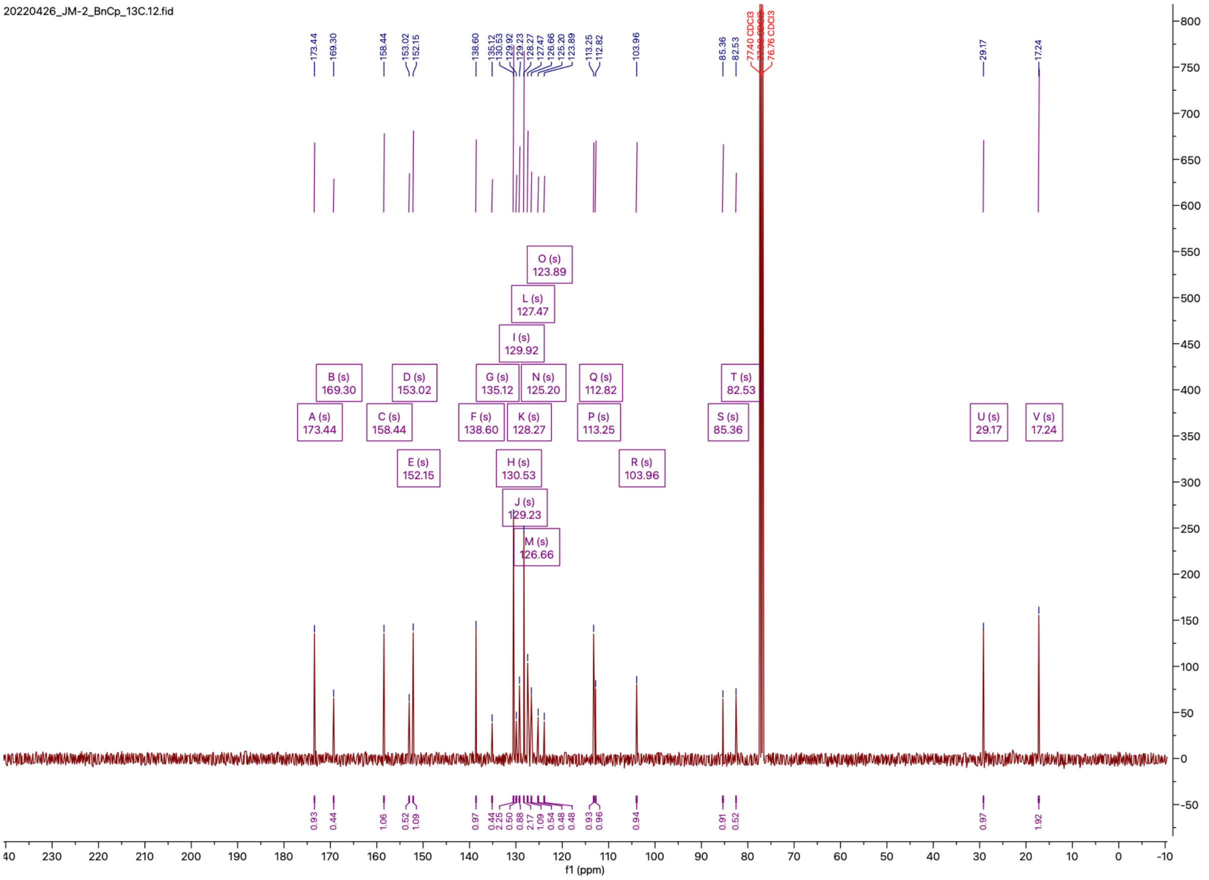

Compound 3: FBM108-Fluorescein

^{1}H NMR spectrum

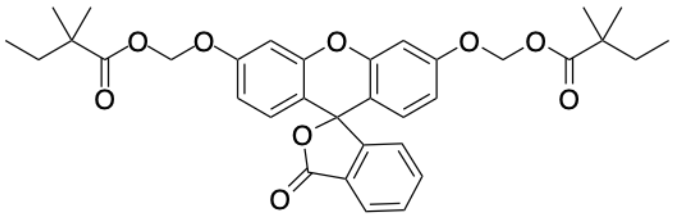

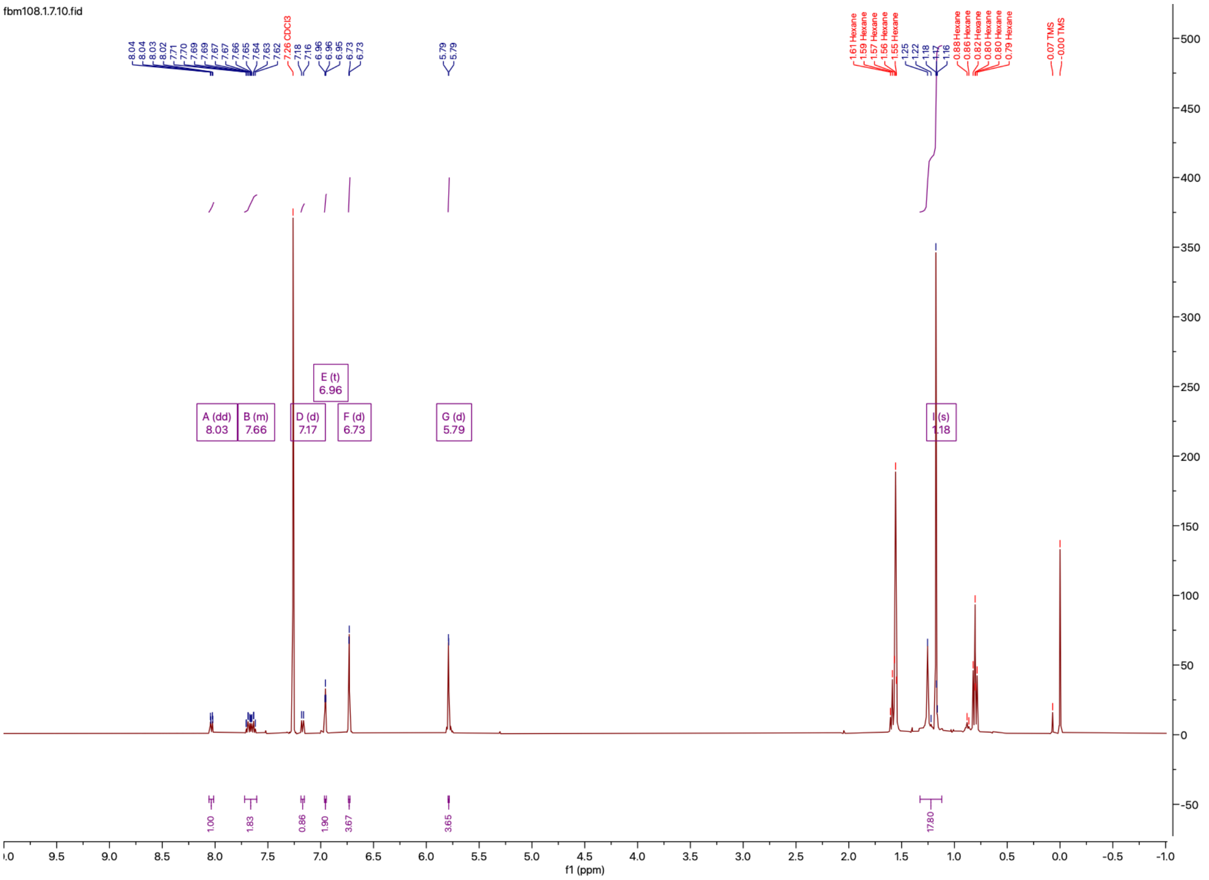

Compound 4: FBM112-Fluorescein

^{1}H NMR spectrum

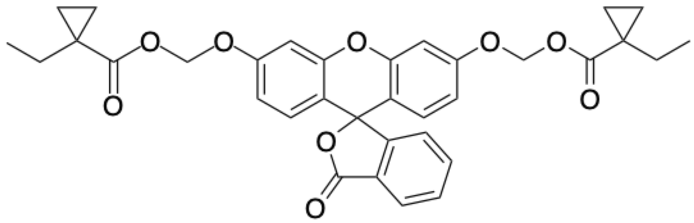

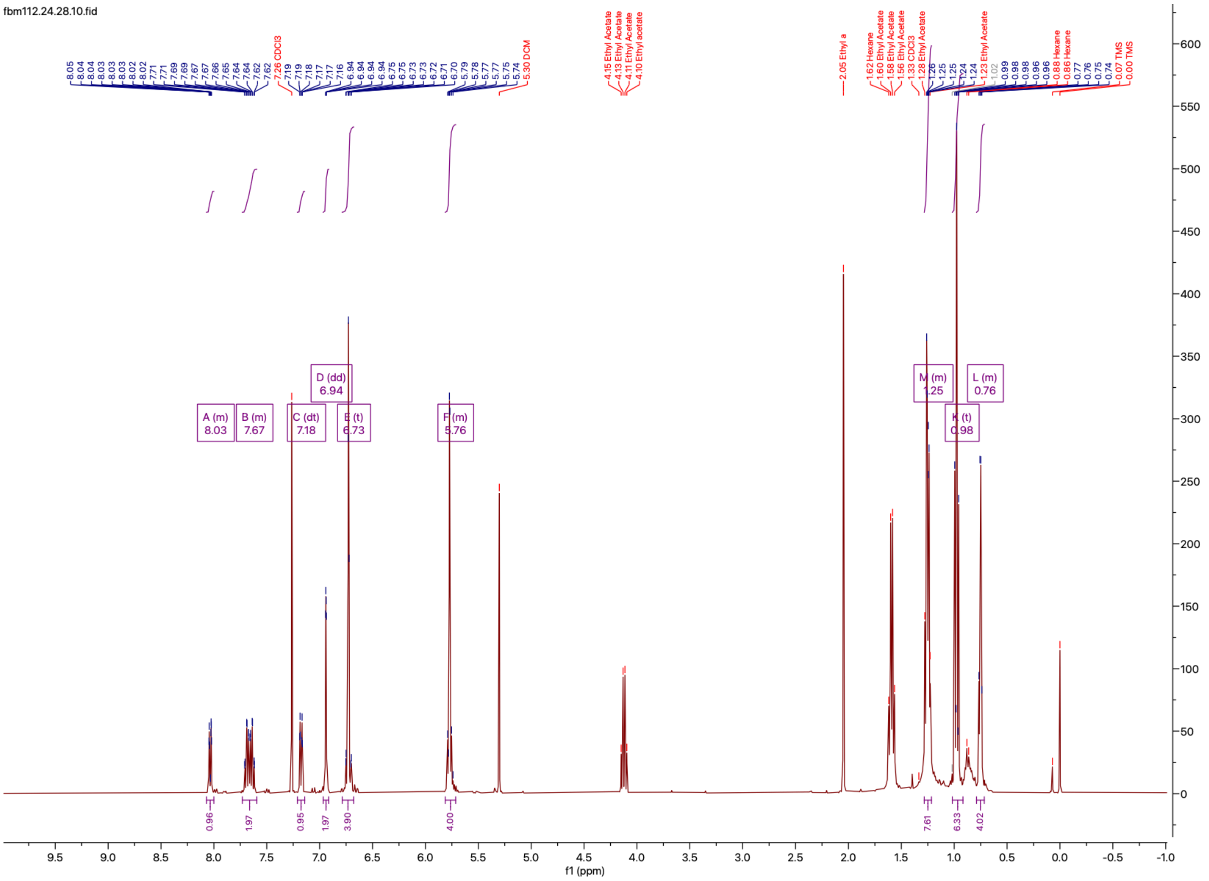

Compound 5: FBM114-Fluorescein

^{1}H NMR spectrum

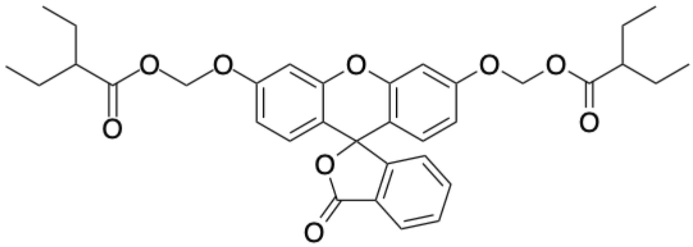

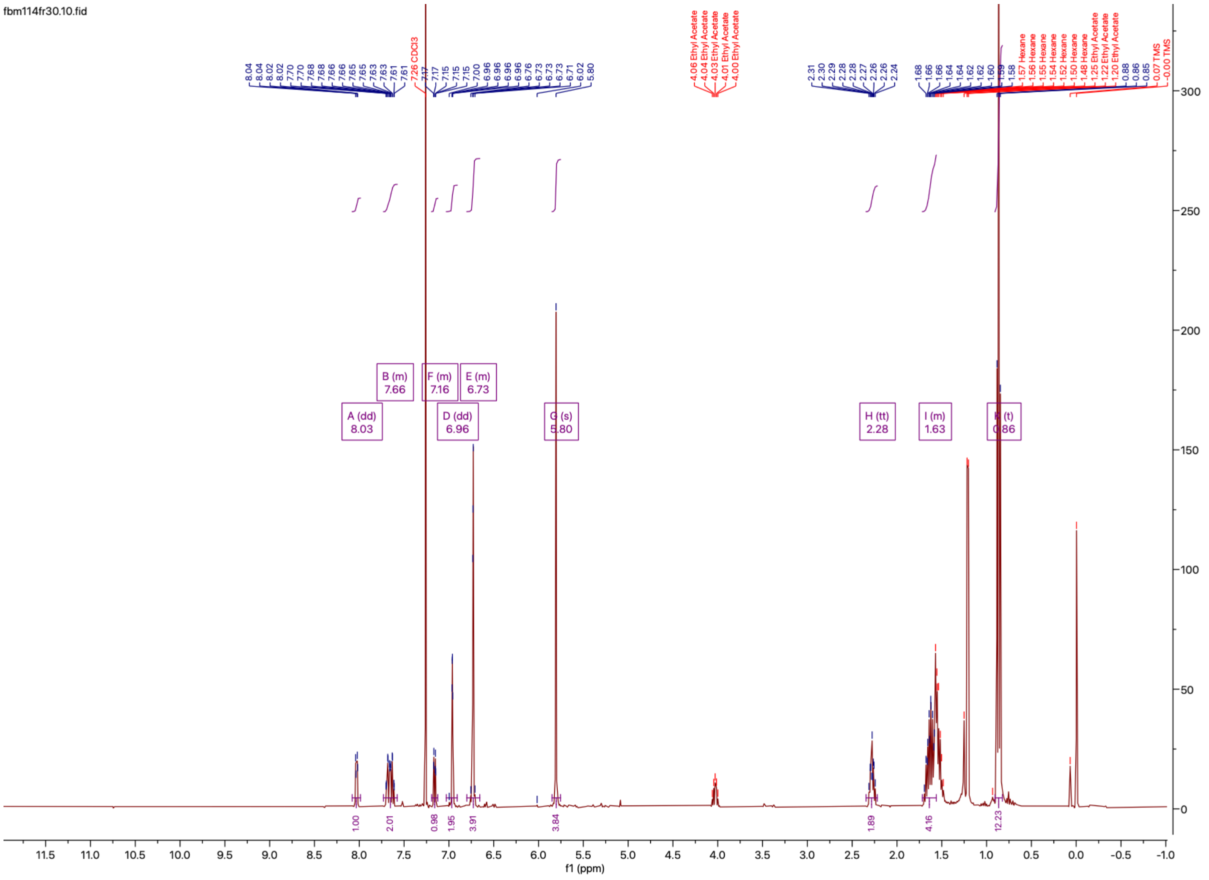

Compound 3:

^{1}H NMR Spectrum

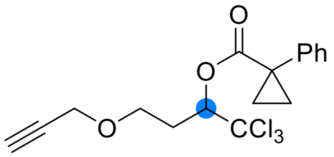

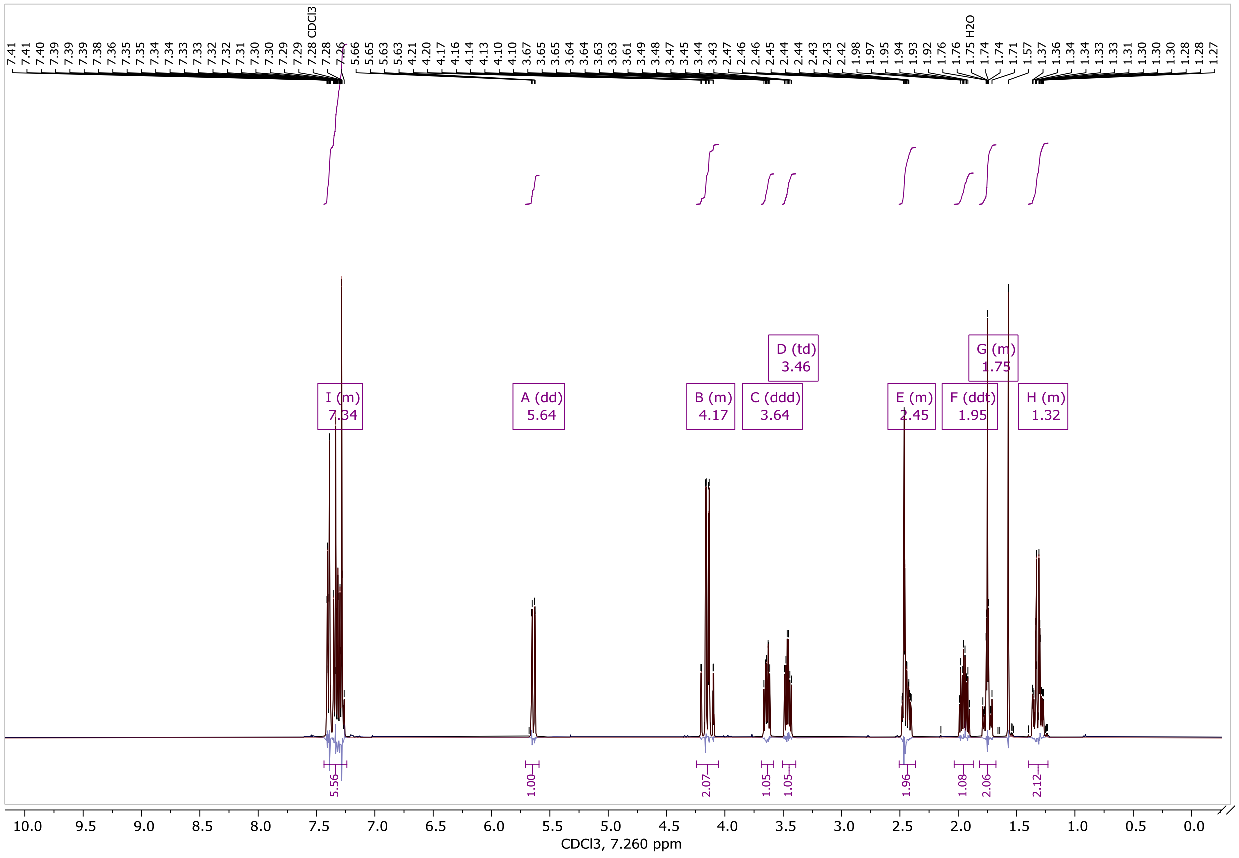

Compound 3:

^{13}C NMR Spectrum

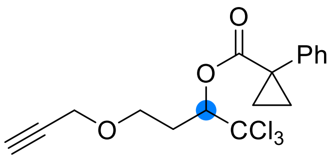

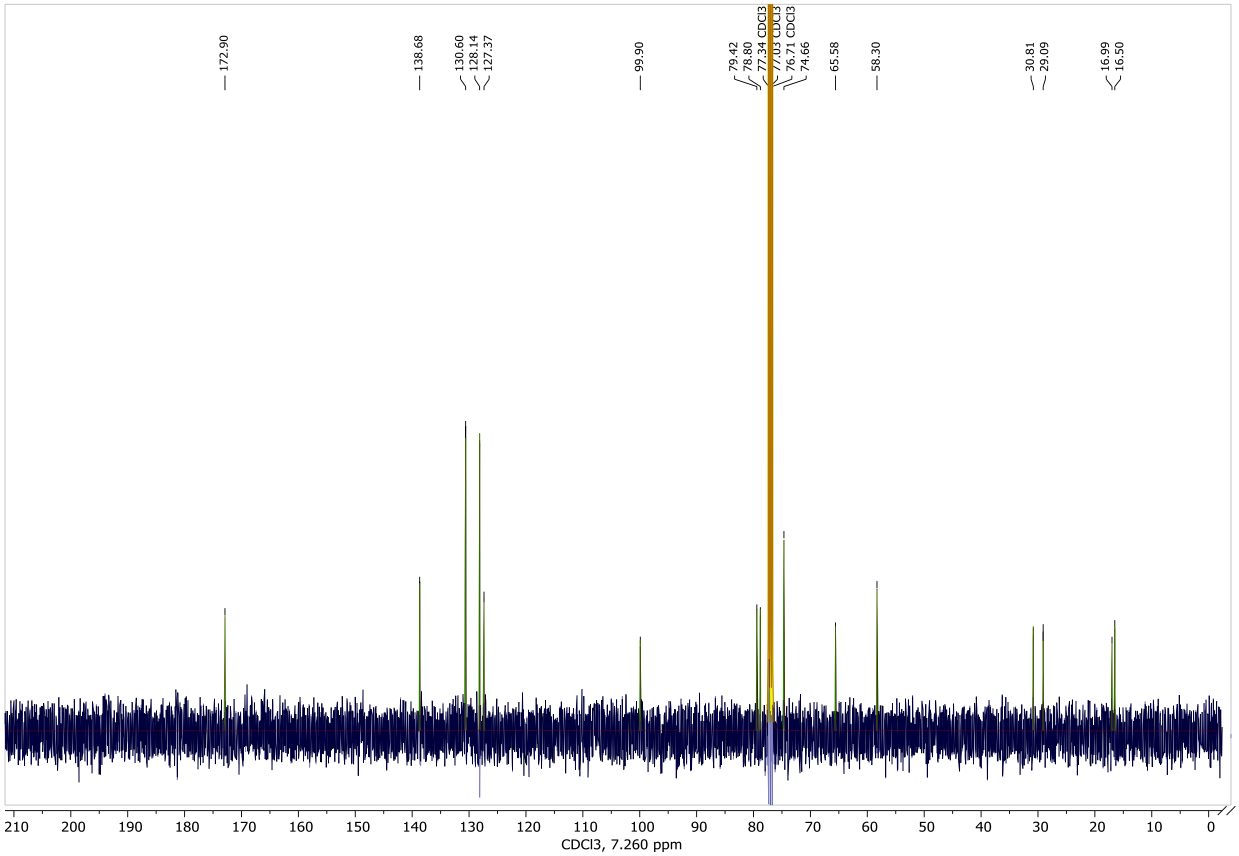

Compound SP-d5:

^{1}H NMR Spectrum

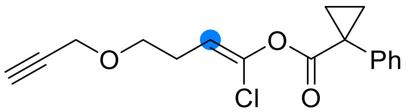

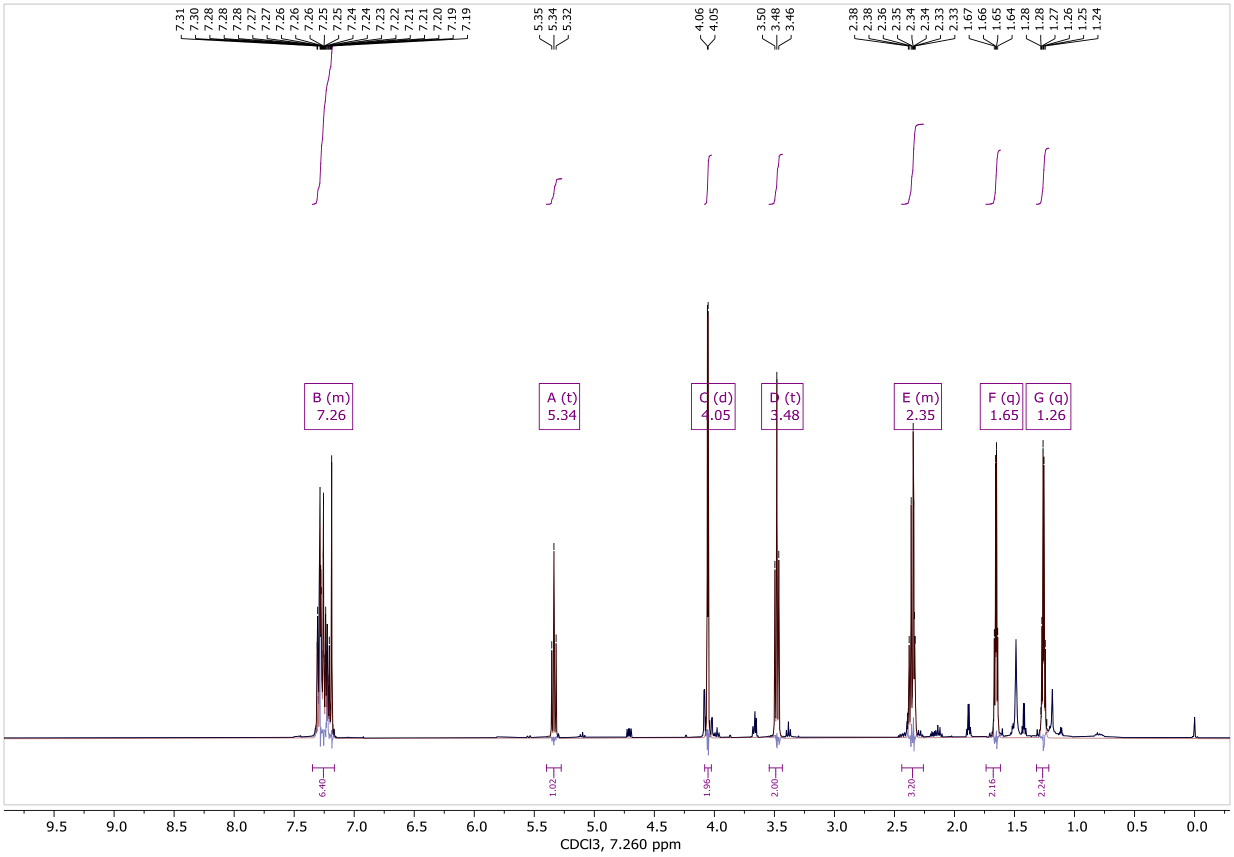

Compound SP-d5:

^{13}C NMR Spectrum

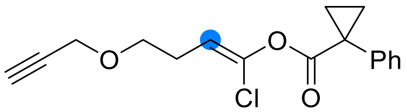

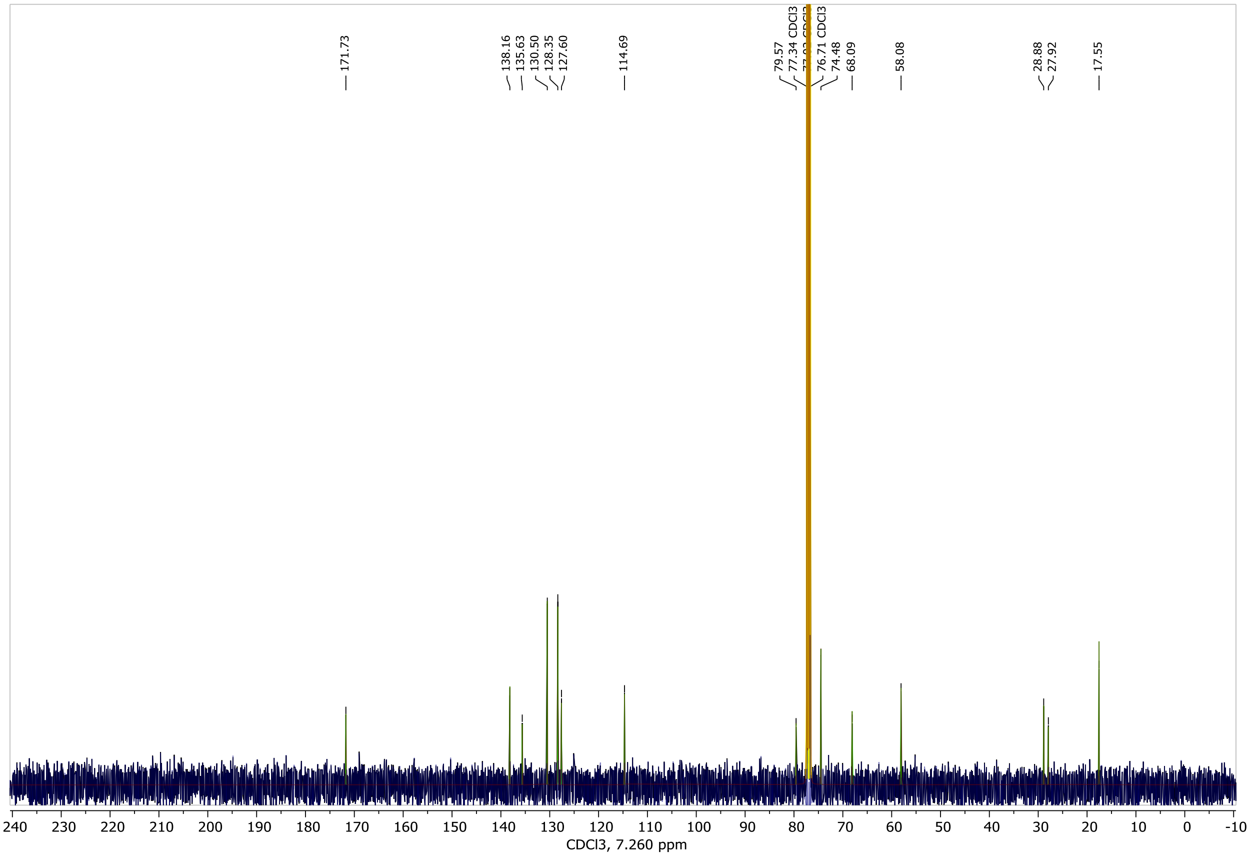

Compound 5:

^{1}H NMR Spectrum

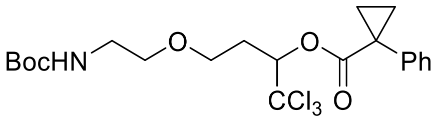

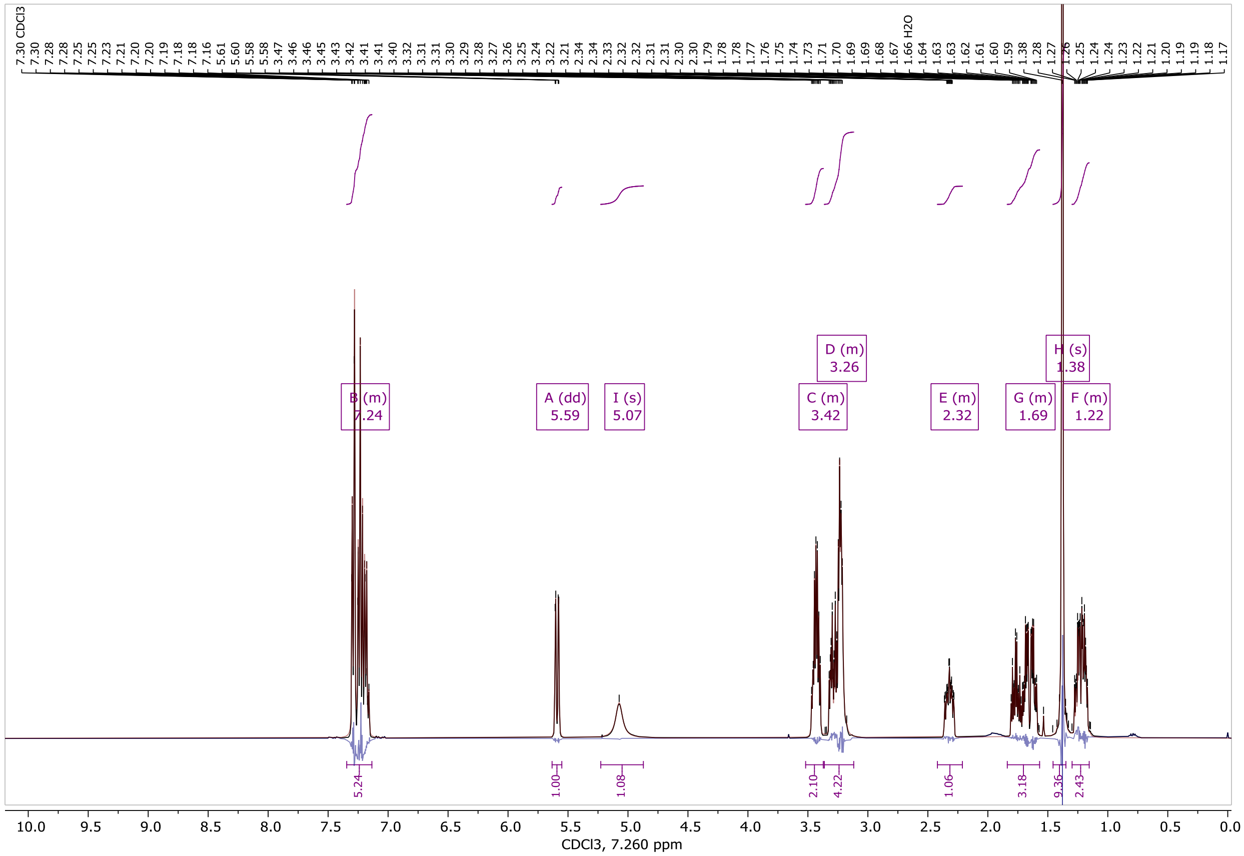

Compound 5:

^{13}C NMR Spectrum

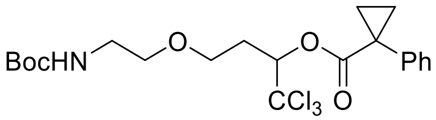

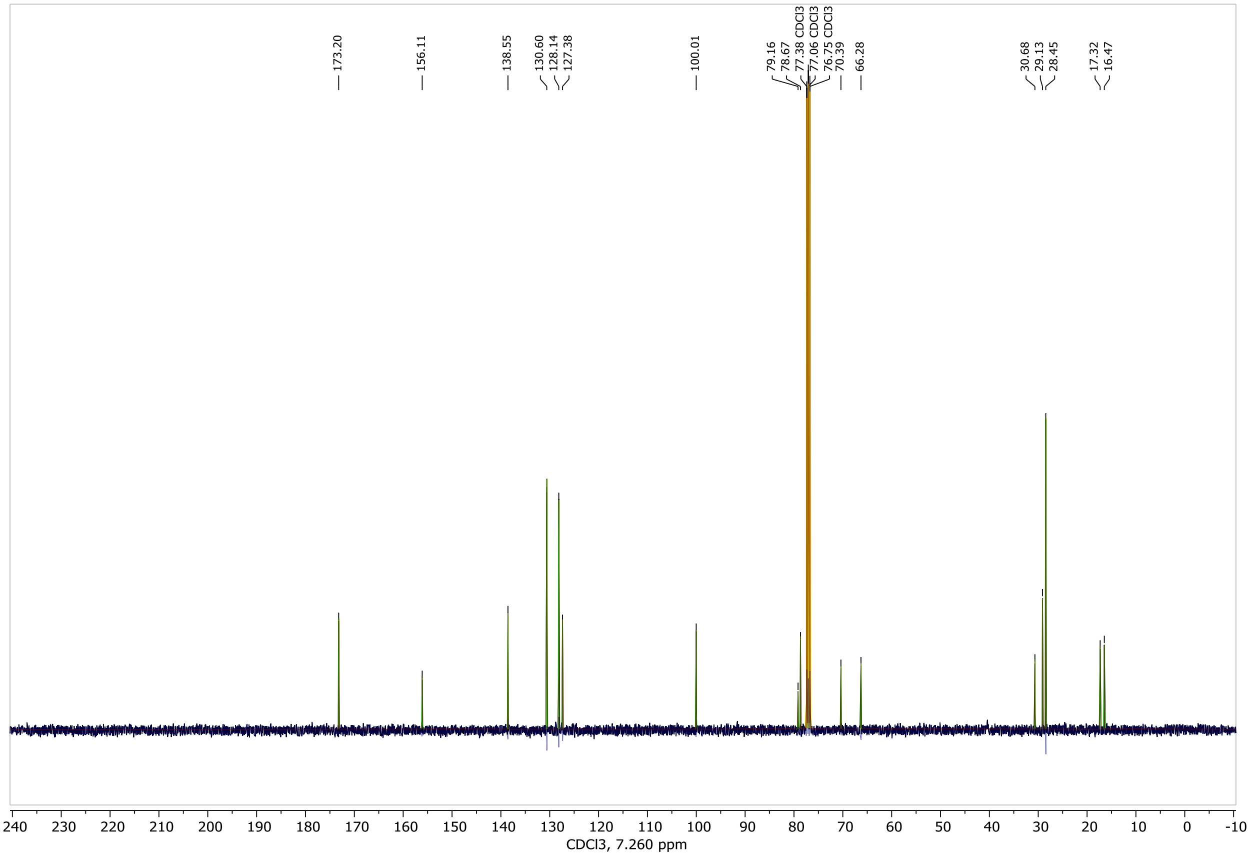

Compound 6:

^{1}H NMR Spectrum

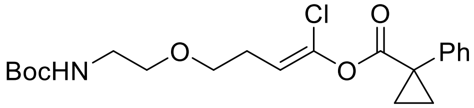

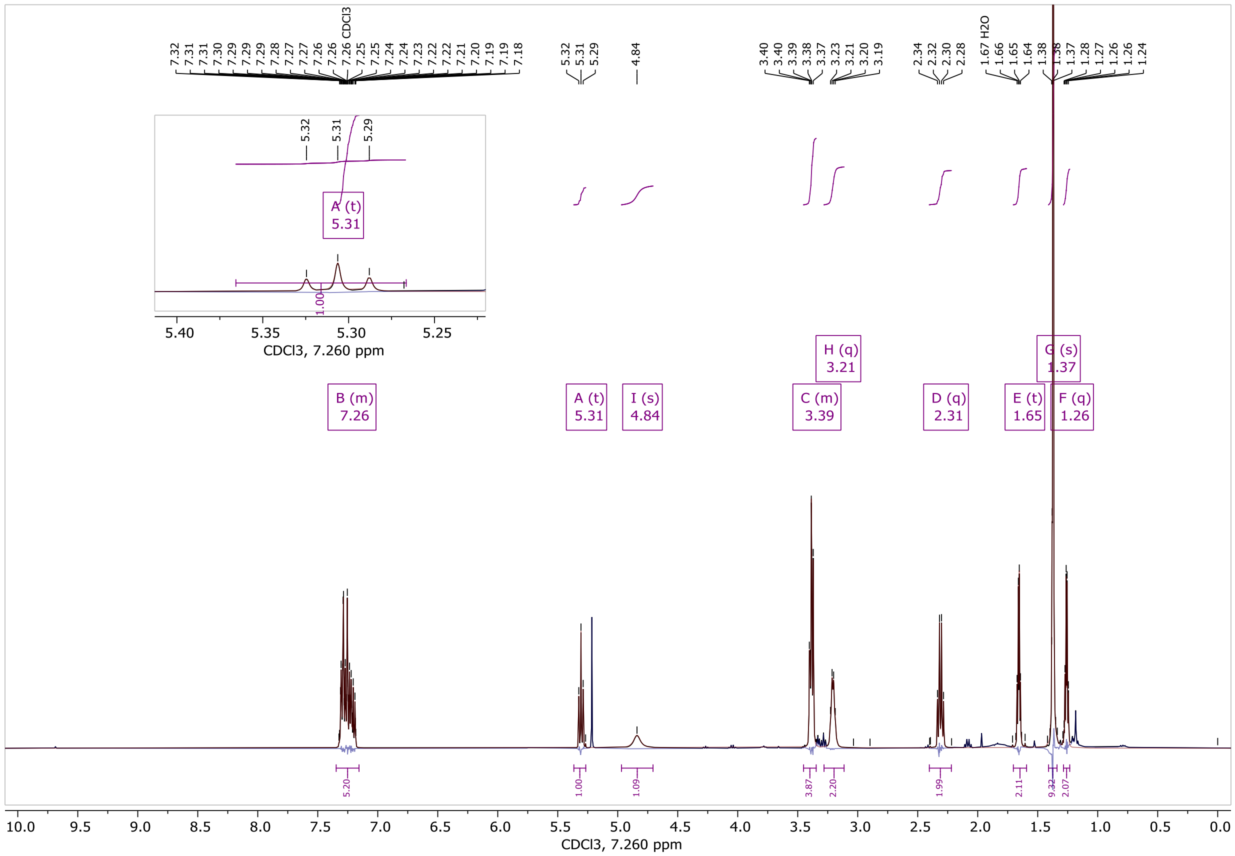

Compound 6:

^{13}C NMR Spectrum

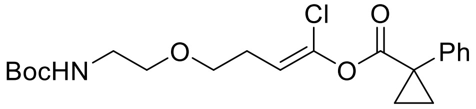

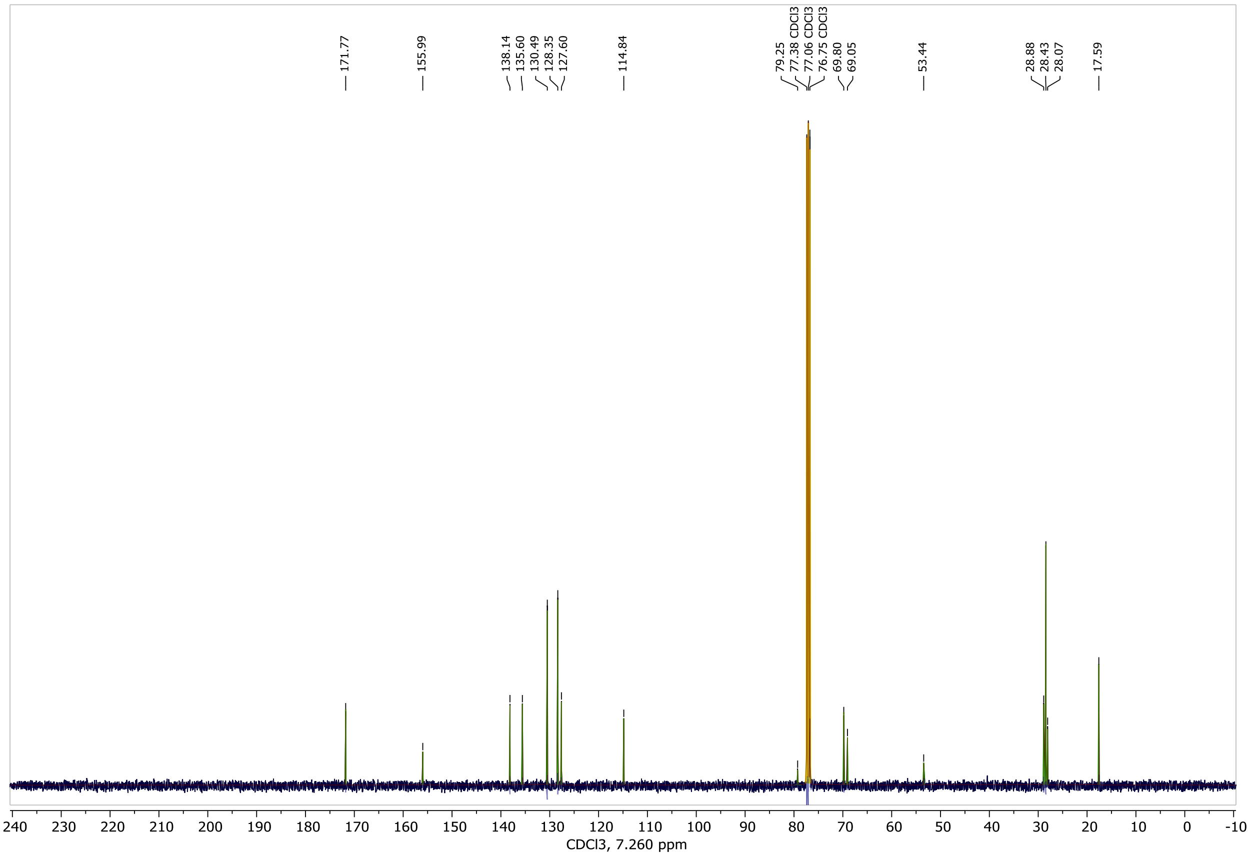

Compound pAC-2:

^{1}H NMR Spectrum

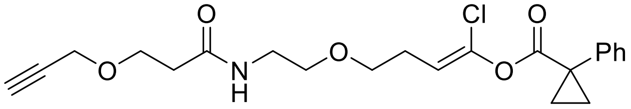

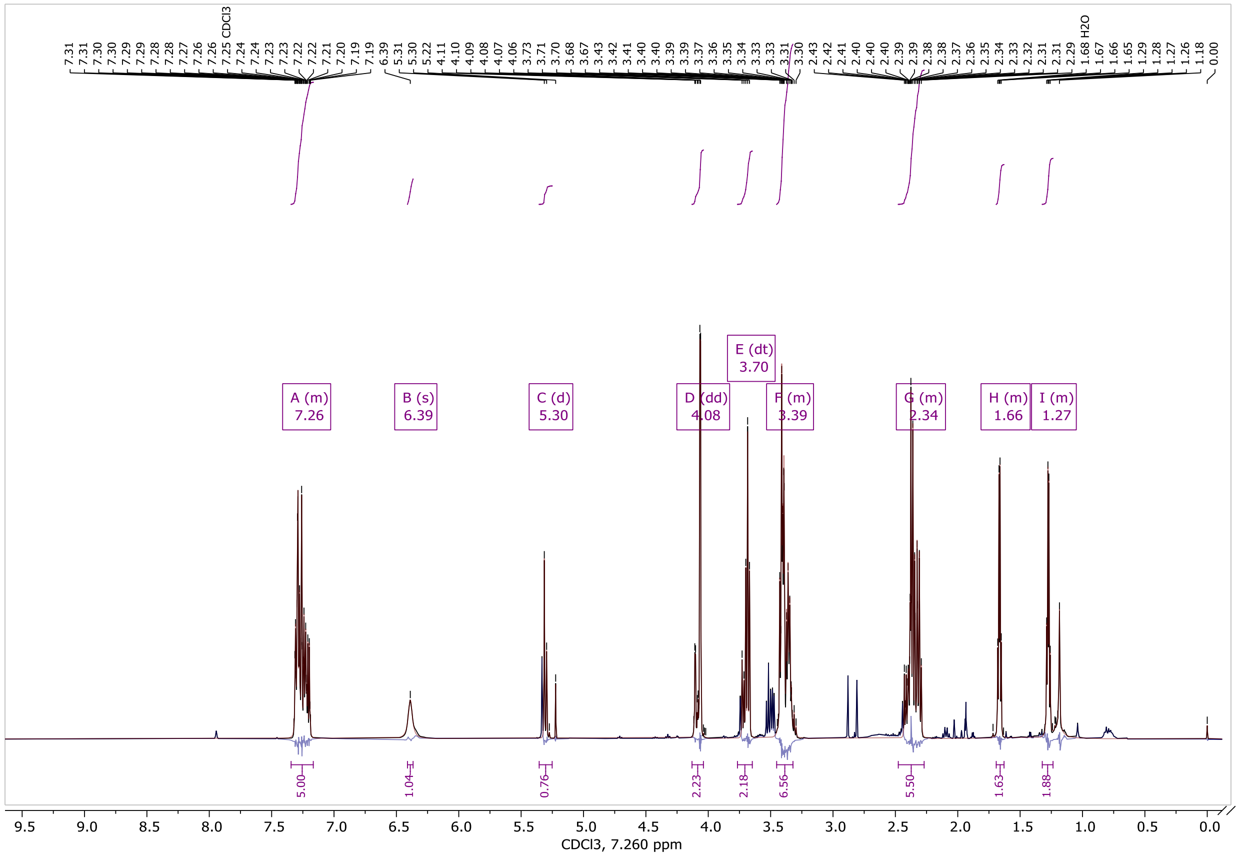

Compound pAC-2:

^{13}C NMR Spectrum

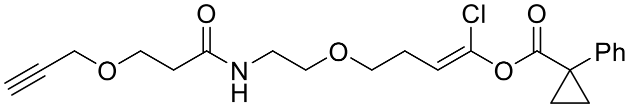

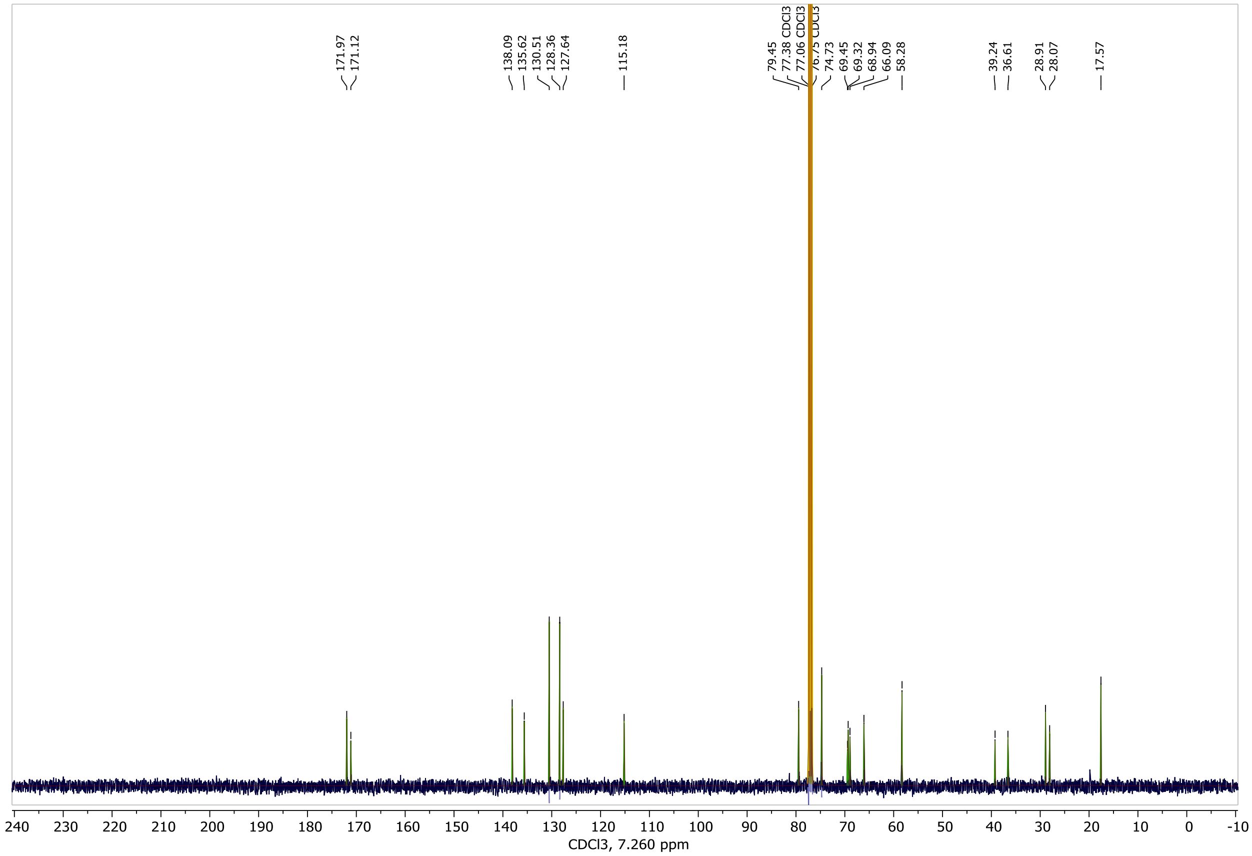

References

(1) Gibson, D. G.; Young, L.; Chuang, R.-Y.; Venter, J. C.; Hutchison, C. A.; Smith, H. O. Enzymatic Assembly of DNA Molecules up to Several Hundred Kilobases. *Nat. Methods* **2009**, *6* (5), 343–345. https://doi.org/10.1038/nmeth.1318.

(2) Martell, J. D.; Yamagata, M.; Deerinck, T. J.; Phan, S.; Kwa, C. G.; Ellisman, M. H.; Sanes, J. R.; Ting, A. Y. A Split Horseradish Peroxidase for the Detection of Intercellular Protein–Protein Interactions and Sensitive Visualization of Synapses. *Nat. Biotechnol.* **2016**, *34* (7), 774–780. https://doi.org/10.1038/nbt.3563.

(3) Pani, S.; Qiu, T.; Kentala, K.; Azizi, S.-A.; Dickinson, B. C. Bioorthogonal Masked Acylating Agents for Proximity-Dependent RNA Labelling. *Nat. Chem.* **2024**, *16* (5), 717–726. https://doi.org/10.1038/s41557-024-01493-1.

(4) Chao, G.; Lau, W. L.; Hackel, B. J.; Sazinsky, S. L.; Lippow, S. M.; Wittrup, K. D. Isolating and Engineering Human Antibodies Using Yeast Surface Display. *Nat. Protoc.* **2006**, *1* (2), 755–768. https://doi.org/10.1038/nprot.2006.94.

(5) Chen, I.; Dorr, B. M.; Liu, D. R. A General Strategy for the Evolution of Bond-Forming Enzymes Using Yeast Display. *Proc. Natl. Acad. Sci.* **2011**, *108* (28), 11399–11404. https://doi.org/10.1073/pnas.1101046108.

(6) Boder, E. T.; Wittrup, K. D. Yeast Surface Display for Screening Combinatorial Polypeptide Libraries. *Nat. Biotechnol.* **1997**, *15* (6), 553–557. https://doi.org/10.1038/nbt0697-553.

(7) Cribari, M. A.; Unger, M. J.; Unarta, I. C.; Ogorek, A. N.; Huang, X.; Martell, J. D. Ultrahigh-Throughput Directed Evolution of Polymer-Degrading Enzymes Using Yeast Display. *J. Am. Chem. Soc.* **2023**, *145* (50), 27380–27389. https://doi.org/10.1021/jacs.3c08291.

(8) Lam, S. S.; Martell, J. D.; Kamer, K. J.; Deerinck, T. J.; Ellisman, M. H.; Mootha, V. K.; Ting, A. Y. Directed Evolution of APEX2 for Electron Microscopy and Proximity Labeling. *Nat. Methods* **2015**, *12* (1), 51–54. https://doi.org/10.1038/nmeth.3179.

(9) Presolski, S. I.; Hong, V. P.; Finn, M. G. Copper-Catalyzed Azide-Alkyne Click Chemistry for Bioconjugation. *Curr. Protoc. Chem. Biol.* **2011**, *3* (4), 153–162. https://doi.org/10.1002/9780470559277.ch110148.

(10) Hong, V.; Presolski, S. I.; Ma, C.; Finn, M. G. Analysis and Optimization of Copper-Catalyzed Azide–Alkyne Cycloaddition for Bioconjugation. *Angew. Chem. Int. Ed Engl.* **2009**, *48* (52), 9879–9883. https://doi.org/10.1002/anie.200905087.

(11) Mok, B. Y.; Kotrys, A. V.; Raguram, A.; Huang, T. P.; Mootha, V. K.; Liu, D. R. CRISPR-Free Base Editors with Enhanced Activity and Expanded Targeting Scope in Mitochondrial and Nuclear DNA. *Nat. Biotechnol.* **2022**, *40* (9), 1378–1387. https://doi.org/10.1038/s41587-022-01256-8.

(12) Walker, J. M. The Bicinchoninic Acid (BCA) Assay for Protein Quantitation. In *The Protein Protocols Handbook*; Walker, J. M., Ed.; Humana Press: Totowa, NJ, 2002; pp 11–14. https://doi.org/10.1385/1-59259-169-8:11.

(13) Baldwin, M. R.; O'Brien, P. J. Human AP Endonuclease 1 Stimulates Multiple-Turnover Base Excision by Alkyladenine DNA Glycosylase. *Biochemistry* **2009**, *48* (25), 6022–6033. https://doi.org/10.1021/bi900517y.

(14) Dixit, C. K.; Vashist, S. K.; MacCraith, B. D.; O'Kennedy, R. Evaluation of Apparent Non-Specific Protein Loss Due to Adsorption on Sample Tube Surfaces and/or Altered Immunogenicity. *Analyst* **2011**, *136* (7), 1406–1411. https://doi.org/10.1039/C0AN00689K.

(15) Abramson, J.; Adler, J.; Dunger, J.; Evans, R.; Green, T.; Pritzel, A.; Ronneberger, O.; Willmore, L.; Ballard, A. J.; Bambrick, J.; Bodenstein, S. W.; Evans, D. A.; Hung, C.-C.; O'Neill, M.; Reiman, D.; Tunyasuvunakool, K.; Wu, Z.; Žemgulytė, A.; Arvaniti, E.; Beattie, C.; Bertolli, O.; Bridgland, A.; Cherepanov, A.; Congreve, M.; Cowen-Rivers, A. I.; Cowie, A.; Figurnov, M.; Fuchs, F. B.; Gladman, H.; Jain, R.; Khan, Y. A.; Low, C. M. R.; Perlin, K.; Potapenko, A.; Savy, P.; Singh, S.; Stecula, A.; Thillaisundaram, A.; Tong, C.; Yakneen, S.; Zhong, E. D.; Zielinski, M.; Žídek, A.; Bapst, V.; Kohli, P.; Jaderberg, M.; Hassabis, D.; Jumper, J. M. Accurate Structure Prediction of Biomolecular Interactions with AlphaFold 3. *Nature* **2024**, *630* (8016), 493–500. https://doi.org/10.1038/s41586-024-07487-w.

(16) Corso, G.; Stärk, H.; Jing, B.; Barzilay, R.; Jaakkola, T. Diffdock: Diffusion Steps, Twists, and Turns for Molecular Docking. *ArXiv Prepr. ArXiv221001776* **2022**.

(17) Fazal, F. M.; Han, S.; Parker, K. R.; Kaewsapsak, P.; Xu, J.; Boettiger, A. N.; Chang, H. Y.; Ting, A. Y. Atlas of Subcellular RNA Localization Revealed by APEX-Seq. *Cell* **2019**, *178* (2), 473-490.e26. https://doi.org/10.1016/j.cell.2019.05.027.

(18) Zhou, Y.; Wang, G.; Wang, P.; Li, Z.; Yue, T.; Wang, J.; Zou, P. Expanding APEX2 Substrates for Proximity-Dependent Labeling of Nucleic Acids and Proteins in Living Cells. *Angew. Chem. Int. Ed.* **2019**, *58* (34), 11763–11767. https://doi.org/10.1002/anie.201905949.

(19) Fang, S.; Acevedo, L. D.; Solivais, A. J.; Zhou, X.; Patil, S. S.; Delfosse, E. S.; Yin, H.; Frey, B. L.; Chapman, E. R.; Smith, L. M.; Martell, J. D. Directed Evolution of APOX for Proximity Labeling Using Phenols with High Redox Potentials. *ChemRxiv* **2025**.

(20) Dobin, A.; Davis, C. A.; Schlesinger, F.; Drenkow, J.; Zaleski, C.; Jha, S.; Batut, P.; Chaisson, M.; Gingeras, T. R. STAR: Ultrafast Universal RNA-Seq Aligner. *Bioinformatics*, 2012, *29*, 15–21.

(21) Patro, R.; Duggal, G.; Love, M. I.; Irizarry, R. A.; Kingsford, C. Salmon Provides Fast and Bias-Aware Quantification of Transcript Expression. *Nat. Methods* **2017**, *14* (4), 417–419.

(22) Soneson, C.; Love, M. I.; Robinson, M. D. Differential Analyses for RNA-Seq: Transcript-Level Estimates Improve Gene-Level Inferences. *F1000Research* **2016**, *4*, 1521.

(23) Law, C. W.; Chen, Y.; Shi, W.; Smyth, G. K. Voom: Precision Weights Unlock Linear Model Analysis Tools for RNA-Seq Read Counts. *Genome Biol.* **2014**, *15* (2), R29.

(24) Ritchie, M. E.; Phipson, B.; Wu, D. I.; Hu, Y.; Law, C. W.; Shi, W.; Smyth, G. K. Limma Powers Differential Expression Analyses for RNA-Sequencing and Microarray Studies. *Nucleic Acids Res.* **2015**, *43* (7), e47–e47.

(25) Tian, L.; Yang, Y.; Wysocki, L. M.; Arnold, A. C.; Hu, A.; Ravichandran, B.; Sternson, S. M.; Looger, L. L.; Lavis, L. D. Selective Esterase–Ester Pair for Targeting Small Molecules with Cellular Specificity. *Proc. Natl. Acad. Sci.* **2012**, *109* (13), 4756–4761. https://doi.org/10.1073/pnas.1111943109.
